# A comprehensive evaluation of polygenic score and genotype imputation performances of human SNP arrays in diverse populations

**DOI:** 10.1038/s41598-022-22215-y

**Published:** 2022-10-20

**Authors:** Dat Thanh Nguyen, Trang T. H. Tran, Mai Hoang Tran, Khai Tran, Duy Pham, Nguyen Thuy Duong, Quan Nguyen, Nam S. Vo

**Affiliations:** 1 Center for Biomedical Informatics, Vingroup Big Data Institute, Hanoi, Vietnam; 2 GeneStory JSC, Hanoi, Vietnam; 3 Institute for Molecular Bioscience, University of Queensland, Brisbane, Australia; 4 Institute of Genome Research, Vietnam Academy of Science and Technology, Hanoi, Vietnam; 5 Faculty of Biosciences, Norwegian University of Life Sciences, Ås, Norway

**Keywords:** Computational biology and bioinformatics, Population genetics

## Abstract

Regardless of the overwhelming use of next-generation sequencing technologies, microarray-based genotyping combined with the imputation of untyped variants remains a cost-effective means to interrogate genetic variations across the human genome. This technology is widely used in genome-wide association studies (GWAS) at bio-bank scales, and more recently, in polygenic score (PGS) analysis to predict and stratify disease risk. Over the last decade, human genotyping arrays have undergone a tremendous growth in both number and content making a comprehensive evaluation of their performances became more important. Here, we performed a comprehensive performance assessment for 23 available human genotyping arrays in 6 ancestry groups using diverse public and in-house datasets. The analyses focus on performance estimation of derived imputation (in terms of accuracy and coverage) and PGS (in terms of concordance to PGS estimated from whole-genome sequencing data) in three different traits and diseases. We found that the arrays with a higher number of SNPs are not necessarily the ones with higher imputation performance, but the arrays that are well-optimized for the targeted population could provide very good imputation performance. In addition, PGS estimated by imputed SNP array data is highly correlated to PGS estimated by whole-genome sequencing data in most cases. When optimal arrays are used, the correlations of PGS between two types of data are higher than 0.97, but interestingly, arrays with high density can result in lower PGS performance. Our results suggest the importance of properly selecting a suitable genotyping array for PGS applications. Finally, we developed a web tool that provides interactive analyses of tag SNP contents and imputation performance based on population and genomic regions of interest. This study would act as a practical guide for researchers to design their genotyping arrays-based studies. The tool is available at: https://genome.vinbigdata.org/tools/saa/.

## Introduction

Over the last decade, low-cost, robust genotyping platforms and large-scale genome variation projects such as the 1000 Genomes Project^1^ have facilitated genome-wide association studies (GWAS) on numerous human phenotypes, ranging from height to diseases^2^. To date, thousands of DNA loci that are significantly associated with complex traits and diseases have been discovered^3^. Among numerous possible applications of GWAS results, disease risk prediction is rapidly gaining broad interest recently^4–6^. A polygenic score (PGS) or polygenic risk score (PRS) is an estimate of an individual’s genetic liability to a trait or disease, calculated based on their genotype profile and relevant GWAS data^7^. In its most common form, a PGS is computed as the sum of allele count of risk alleles (0, 1, or 2) that are weighted by its effect size (i.e. log odd ratio or beta coefficient) of hundreds to thousands of associated SNPs. The outcome is a single score that aggregates each individual’s genetic loading proportional to the risk of a given disease or a quantitative trait^6^. Although the clinical utility of PGS has yet to be established, recent works have suggested that PGS may be used for disease risk stratification that potentially facilitates early disease detection, assists in diagnosis, or informs treatment choices^4,5^. For example, PGS of coronary artery disease, type 2 diabetes, and breast cancer at the top 8, 3.5, and 1.5% are risks equivalent to a monogenic mutation risk that confers an odds ratio of 3^8^.

Similar to GWAS analysis, PGS can be derived from various types of genotyping data such as those obtained by single-nucleotide polymorphism (SNP) microarrays or whole-genome sequencing (WGS). While WGS is attractive of the ability to interrogate variations across the entire human genome, SNP arrays are the dominant assays to obtain genetic data for PGS calculation. They come up with several advantages such as cost-effectiveness and light computational requirement which are preferable for population-scale screening, where PGS would be most useful^9^. Because the coverage of SNP arrays is typically limited to lower than a million SNPs, a procedure involving haplotype phasing and genotype imputing of missing sites is usually employed to add more genotyping information that can increase the power of these genetic studies^7,10,11^. The imputation performance is affected by three main factors, including algorithms of choice^12^, imputation reference panels^13,14^, and the SNP array designs^15^.

In principle, genotyping SNP arrays are designed by selecting a set of SNPs, commonly referred to as “tag SNPs”, which maximize coverage of ungenotyped DNA variants through associations between these alleles in the population (known as linkage disequilibrium, LD)^16,17^. Based on the target population, human genotyping SNP arrays can be classified into three categories optimized for global, super population, or specific to targeted populations. In the early phase of development, genotyping SNP arrays were focused on common genetic variations of the whole world population (minor allele frequency, MAF, of 0.10 or greater) based on the HapMap catalog^18^. The second generation of SNP arrays was designed to cover variants with MAF as low as 0.01 by providing SNP arrays specifically for European, East Asian, African American, and Latino race/ethnicity populations based on the 1000 Genomes Project (1KGP) catalog^19,20^. However, the fact that the majority of human genetic variants are rare and population-specific demands customizing SNP arrays to improve over those designed for global or super populations^21,22^. Indeed, population-specific genotyping arrays such as the UK Biobank Axiom Array^2^, the Axiom-NL Array^23^, the Japonica and Japonica NEO Arrays^24,25^, and the Axiom KoreanChip^26^ have been developed on top of the many existing commercial arrays. These arrays are not only optimized for genomic coverage based on their unique variant catalogs but also include a large number of functional variants. For example, the Axiom KoreanChip contains more than 200,000 nonsynonymous loci and the new Japonica NEO Arrays were designed with abundant disease risk variants^25,26^.

The development of customized arrays accompanied by commercial arrays provided by genotyping platform producers results in a large number of genotyping arrays. Each of these arrays has specific properties and contents, and thus, there is an urgent demand for a systematic guideline to determine which array best suits specific research questions and populations. Although there are SNP array comparative studies, they are either not updated with the many recent arrays^15,27^, or limited in only testing for a small set of populations, and some studies focused on LD coverage^27,28^ that may not be relevant to current imputation practice for use in association studies and PGS analysis^7,11^. Moreover, although PGS is gaining increasing attention, practical evaluation of performance for PGS applications by current genotyping arrays is still lacking. Here, we provide a comprehensive evaluation of imputation-based genomic coverage^15,29^ and PGS performance of 23 human genotyping arrays in diverse populations. These analyses are intended to be a practical guide for researchers in selecting the most suitable genotyping array for their genetic studies.

## Materials and methods

### Genotyping arrays

In this study, we benchmarked 23 different human genotyping arrays including 14 arrays from Illumina and 9 arrays from Affymetrix. The examined arrays contain the numbers of tag SNPs (array size) ranging from approximately 300,000 (Infinium HumanCytoSNP-12 v2.1) up to more than 4,300,000 (Infinium Omni5 v1.2). They can be classified as old arrays such as the Genome-Wide Human SNP Array 6.0; population-specific optimized arrays such as Axiom UK Biobank Array and Axiom Japonica Array NEO; multiple populations optimized arrays such as Infinium Multi-Ethnic Global v1.0 and Infinium Global Diversity Array v1.0; cytogenetics and cancer applications optimized arrays such as Infinium CytoSNP-850K v1.2. Recently developed arrays include Infinium Global Screening Array v3.0, Axiom Precision Medicine Research Array, and Axiom Precision Medicine Diversity Array. Manifests of the 23 examined arrays were obtained from respective manufacturers’ websites. Genomic positions were further harmonized to the UCSC hg38 reference genome coordinate with CrossMap v0.2.6 for those requiring lifted over^30^. Details and component statistics of these arrays are shown in Table 1.

**Table 1:** Details of 23 human genotyping arrays used in this study.

Array full name	Array short name	No. assays	No. positions	No. autosomal	No.X	No.Y	No.MT
Infinium HumanCytoSNP-12 v2.1	CytoSNP-12	293,552	293,467	276,248	15,082	1444	0
Infinium Core-24 v1.2	Infinium_Core	304,151	304,111	293,850	8097	2003	161
Infinium OncoArray-500K v1.0	Infinium_OncoArray	497,191	496,203	481,495	14,276	312	120
Infinium PsychArray v1.3	PsychArray	592,414	584,233	567,619	14,221	2051	342
Axiom Genome-Wide ASI	Axiom_GW_ASI	629,494	629,492	609,774	17,263	2222	233
Infinium Global Screening Array v3.0	Infinium_GSA	654,027	648,380	616,080	26,635	3822	987
Axiom Genome-Wide CHB	Axiom_GW_CHB	656,638	656,625	631,283	24,267	980	95
Axiom Japonica Array NEO	Axiom_JAPONICA	671,123	666,782	652,237	13,336	779	409
Axiom Genome-Wide EUR	Axiom_GW_EUR	674,287	673,449	659,956	13,104	290	99
Infinium Chinese Genotyping Array v1.0	Infinium_Chinese	695,116	682,199	647,335	27,668	6210	986
Infinium Japanese Screening Array v1.0	Infinium_JSA	719,938	707,559	675,012	26,223	4686	948
Axiom UK Biobank Array	Axiom_UKB	843,755	820,407	798,493	20,827	813	274
Infinium CytoSNP-850K v1.2	CytoSNP-850K	845,050	842,682	811,217	29,666	1097	0
Axiom Precision Medicine Research Array	Axiom_PMRA	919,099	900,406	864,096	36,132	8	170
Axiom Precision Medicine Diversity Array	Axiom_PMDA	921,664	900,770	837,511	62,039	448	714
Genome-Wide Human SNP Array 6.0	Affymetrix_6.0	931,991	929,011	889,847	37,894	859	411
Infinium OmniZhongHua v1.4	OmniZhongHua	1,170,268	1,165,100	1,134,324	28,444	2220	112
Infinium Multi-Ethnic EUR/EAS/SAS v1.0	Multi-Ethnic_EUR_EAS_SAS	1,471,475	1,471,475	1,429,754	39,479	1598	644
Infinium Multi-Ethnic Global v1.0	Multi-Ethnic_Global	1,748,250	1,733,356	1,673,788	50,914	3569	776
Infinium Global Diversity Array v1.0	Infinium_GDA	1,904,599	1,825,277	1,752,897	60,512	5744	1115
Axiom Genome-Wide PanAFR	Axiom_GW_PanAFR	2,264,666	2,264,432	2,195,556	65,949	2647	280
Infinium Omni2.5 v1.5	Infinium_Omni2.5	2,373,357	2,363,610	2,311,073	50,841	1515	181
Infinium Omni5 v1.2	Infinium_Omni5	4,327,108	4,245,106	4,131,134	106,418	2396	207

Short name of arrays are used interchangeablely with its full names throughout the texts, tables, and figures. No.Assays: number of assays included in the array; No.Positions: number of variants included in the array; No.Autosomal: number of variants of autosomal chromosomes included in the array; No.X, No.Y, and No.MT: number of variants of X, Y, MT chromosomes included in the array respectively.

### Genomic datasets and pipelines

**Figure 1:**
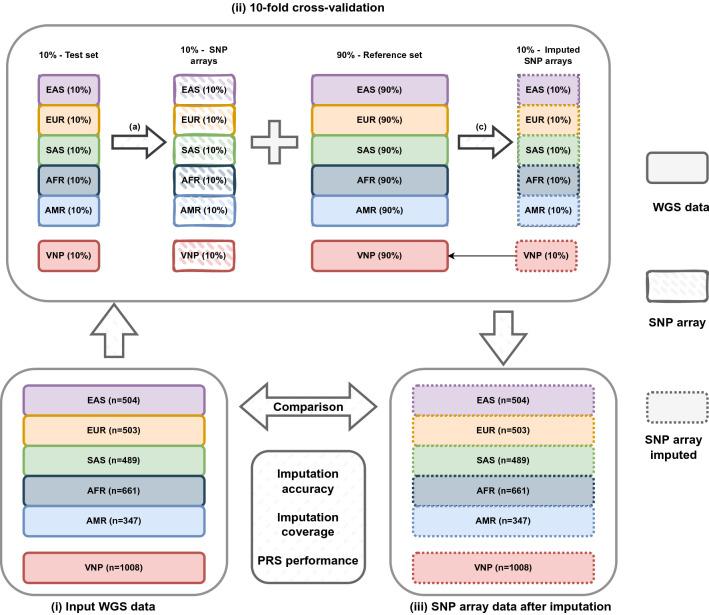
Overview of evaluation pipeline. (i) Two input genetic datasets, including the 1KGP and 1KVG were randomly divided into 10 batches that are equally distributed by populations. (ii) tenfold cross-validation procedure. In each turn, variants of 10% samples were extracted based on arrays’ manifest to generate simulated array genotyping data (arrow a) as input for phasing and imputation with the remaining 90% samples used as the reference set to generate the imputed SNP array data (arrow c). (iii) SNP array data after imputation. Imputed SNP array data of 10 batches were merged according to populations after tenfold cross-validation and were then benchmarked by treating the input WGS data as the golden standard.

An overview of our evaluation pipeline is presented in Fig. 1. In brief, the phased genomic data of 22 autosomal chromosomes in Variant Call Format (VCF) of 2,504 and 1,008 unrelated individuals from the 1000 Genomes Project samples that were re-sequenced by New York Genome Center (1KGP)^31^ and the 1000 Vietnamese Genomes Project (1KVG)^32^, respectively, were used to estimate imputation-based coverage and PGS performance of 23 different genotyping arrays by the tenfold cross-validation approach. In the 1KGP dataset, 26 populations were grouped into 5 supper-populations according to their continental groups including East Asian (EAS), European (EUR), South Asian (SAS), African (AFR), and American (AMR). For consistent naming throughout the text, these continental groups are hereafter considered as a population. This dataset was randomly divided into 10 batches equally distributed across populations (4 batches with 251 samples and 6 batches with 250 samples). Similarly, the Vietnamese population (VNP) was processed separately with 8 batches of 101 and 2 batches of 100 samples. In each turn, one batch was used as the test set and the remaining samples as the reference set. For each array, variants in the test set with the same position as variants on the array were extracted with vcftools v0.1.17^33^ and phasing information was removed to generate the pseudo SNP array genotyped data, while variants in reference data were used as the pre-phasing and imputation reference panel. The pre-phasing and imputation were performed with SHAPEIT v4.1.3^34^ and Minimac4 v1.0.2^12^ respectively. Finally, the imputed genotyping data of 10 batches were combined to estimate imputation and PGS performance according to their populations, including 504, 503, 489, 661, 347, and 1,008 individuals in EAS, EUR, SAS, AFR, AMR, and VNP, respectively. This approach is similar to the strategy used previously to estimate imputation-based genomic coverage^15,29,35^.

### Imputation performance evaluation

Both GWAS and PGS often require genotype imputation that involves the prediction of untyped variants in the genome. While GWAS benefits from boosting the number of imputed SNPs that can be tested for association^11^, computation of PGS is conducted by summing the product of risk allele count (0, 1, or 2) and its effect size derived from the GWAS. Thus, imputation performance is expected to play a key role in PGS derivation. Here, we focus on imputation $r^2$ metric although there are several other criteria that can be used to assess imputation performance such as allele concordance^15^, imputation quality^28^, LD coverage^36^. We choose imputation $r^2$ as the evaluation metric for the following reasons. First, it is more relevant to the context of GWAS and PGS analysis because the imputation $r^2$ at a given variant is proportional to its $\chi ^2$ statistic that results from an association test^37–40^. This leads to the interpretation that an increase in mean imputation $r^2$ at genome wide scale directly corresponds to the increase of statistical power^37,40^. Second, it is less sensitive to allele frequency than concordance^15^. Third, it incorporates imputation uncertainty by using expected allele dosage rather than the most likely genotype^15^. Finally, imputation $r^2$ can be computed on a site-by-site basis, which enables a more detailed evaluation than at the allele frequency level^40^. In this evaluation setting, we treated genotypes derived from WGS datasets as gold standard. Imputation performance is measured as imputation $r^2$ that is SNP-wise squared Pearson’s correlation between the imputed dosages and the WGS genotypes, and imputation coverage is defined as the proportion of SNPs with imputation $r^2$ passing the cut-off of 0.8. These metrics were stratified into three minor allele frequency (MAF) bins, including (0–0.01], (0.01–0.05], (0.05–0.5]. To reduce the data noise, multiallelic sites were not considered, and variants with allele count less than 2 were excluded in the bin of (0–0.01]. Of note, the MAF bin of (0.01–0.5], which is the most common cutoff for GWAS and PGS analysis, was also considered in the analysis^7,41^.

### PGS performance assessment

Instead of using pre-tuned PGS models as in other studies^9,40^, PGS was computed with a standard P+T (Prunning and Thresholding) approach implemented in PRSice-2^42^ in this study. The main reason for using this approach is that we tried to mimic the real-life practice of PGS analysis that involves running a PGS computational method with multiple parameters and selecting the best one^7^. Another reason is that using pre-built PGS models may introduce a potential bias for some specific arrays as they were used in tuning in these established PGS model, i.e., we tried to avoid training using the same array twice. Using summary statistics for three phenotypes, namely height, body mass index (BMI), and type 2 diabetes (T2D), obtained from previous GWAS meta analyses^43,44^, a PGS for an individual *i* was calculated as:

1 $$\begin{aligned} PGS_i(P_T) = \sum _{j=1}^{M} {\textbf {1}}_{\{P_j < P_T\}}x_{ij}\hat{\beta _j}, \end{aligned}$$

where $P_T$ is the p-value threshold values (5e−08, 1e−07, 1e−06, 1e−05, 0.0001, 0.001, 0.01, 0.1, 0.2, 0.3, 0.5, and 1); *M* is number of SNPs after clumping with *“–clump-kb 250kb”* and *“–clump-r2 0.1”*; $x_{ij}$ and $\hat{\beta _j}$ is the allele count and the marginal effect size derived from GWAS summary statistics of $SNP_j$.

Similar to imputation performance evaluation, we treated PGS derived from WGS as the “gold standard”. PGS derived from 23 different SNP arrays were evaluated using Pearson’s correlation to PGS derived from WGS data under the same PRSice-2 parameter settings. In addition, absolute differences in PGS percentile ranking generated by array-imputed and the WGS data were also evaluated.

### Ethics approval and consent to participate

The study did not generated new dataset. Ethics approval and consent to participate were applied according to corresponding orginal works. In the 1KVG study, subjects provided informed consent and the study was approved by the Vinmec International Hospital Institutional Review Board with number 543/2019/QD-VMEC in accordance with the relevant guidelines and regulations (e.g. Helsinki Declaration). In the 1KGP-NYGC study, genetic data are publicly available according to the original ethics approval.

## Results

### Imputation performance

**Figure 2:**
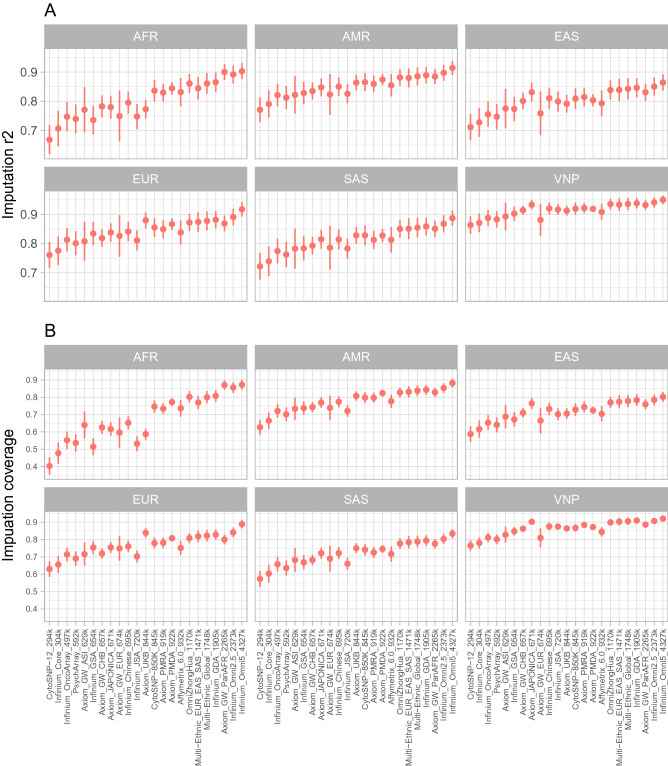
(**A**) Mean imputation $r^2$, and (**B**) Imputation coverage across 22 autosomes of 23 SNP arrays in the MAF bin of (0.01–0.5]. The dots and the vertical lines present the mean and the standard deviation of imputation accuracy, and imputation coverage values in 22 autosomes respectively.

Overall, we found two main factors affecting the imputation accuracy and imputation coverage that are array sizes and population optimization. The two densest arrays that are the Infinium Omni2.5 v1.5 and Infinium Omni5 v1.2 with approximately 2.4 and 4.3 minion tag SNPs yielded the highest imputation performance. In contrast, the two sparsest SNP arrays with approximately 300,000 tag SNPs that are Infinium HumanCytoSNP-12 v2.1 and Infinium Core-24 v1.2 obtained the poorest imputation performance in all six examined populations. At the MAF bin of (0.01–0.5], the Infinium Omni5 v1.2 yielded the mean imputation accuracy $r^2$ of 0.9032, 0.9144, 0.8644, 0.9176, 0.8873, 0.9499 and the imputation coverage of 0.8721, 0.8813, 0.8019, 0.8885, 0.8344, 0.9207 while the Infinium HumanCytoSNP-12 v2.1 obtained 0.6682, 0.7708 0.7112, 0.7608 0.7218, 0.8635 for mean imputation accuracy $r^2$ and 0.4031, 0.6265, 0.5879, 0.6297, 0.5731, 0.7655 for imputation coverage in six populations AFR, AMR, EAS, EUR, SAS, and VNP respectively. Details are reported in Fig. 2 and Tables 2, 3.

Regarding population optimization, imputation performance is generally better for those arrays optimized specifically for the targeted populations. For example, the Axiom UK Biobank Array, which was optimized for the British population, performed superiorly in the EUR than most other arrays (except for the ultra-high-density arrays Infinium Omni2.5 v1.5 and Infinium Omni5 v1.2). In detail, at the MAF bin of (0.01–0.5], The Axiom UK Biobank Array with the size of 844k SNPs obtained the mean imputation coverage of 0.8389 which was higher than globally optimized, higher density arrays such as Axiom Precision Medicine Research Array (919k), Axiom Precision Medicine Diversity Array (922k), Genome-Wide Human SNP Array 6.0 (932k), Infinium Multi-Ethnic Global v1.0 (1784k), and Infinium Global Diversity Array v1.0 (1905k), with imputation coverage of 0.7814, 0.8078, 0.7513, 0.8228, 0.8277, respectively and lower 0.8409, and 0.8885 that were obtained by Infinium Omni2.5 v1.5 and Infinium Omni5 v1.2 arrays with 2373k and 4327k SNPs. Similarly, the Axiom Japonica Array NEO (671k) which was designed for the Japanese population also performed well against global optimized, higher-density arrays. These two arrays yielded mean imputation accuracy of 0.831, 0.9333; and imputation coverage of 0.7642, 0.9024 in EAS and VNP populations. These performances were higher than those of multi-ethics SNP arrays, even with higher density including Axiom Precision Medicine Research Array (919k), Axiom Precision Medicine Diversity Array (922k), Genome-Wide Human SNP Array 6.0 (932k) as showed in Fig. 2 and Tables 2, 3. Notably, the Infinium OmniZhongHua v1.4 (Chinese optimized array) also outperformed other arrays in EAS and VNP populations. Regarding the AFR population, an array optimized for this population is Axiom Genome-Wide PanAFR with 2265k SNPs performed nearly equivalent the Infinium Omni5 v1.2 array with 4327k SNPs (0.9002 versus 0.9032 for mean imputation accuracy, and 0.8700 versus 0.8721 interns of imputation coverage). There were no SNP arrays with superior performances in the two remaining populations (AMR and SAS), although the Axiom UK Biobank Array and the Axiom Genome-Wide ASI obtained slightly better performance than other arrays with the same sizes when applied for the AMR and SAS populations. In this case, we focused on the MAF bin of (0.01–0.5] as this is the most common cutoff allele frequency in both GWAS and PGS analysis^7,45^. However, the results were also generalized for other bins as shown in Fig. S.1 and Table S.1–6.

**Table 2:** Mean and the standard deviation of imputation accuracy $r^2$ measured in 22 autosomes at the MAF bin of (0.01–0.5].

Array name	AFR	AMR	EAS	EUR	SAS	VNP
CytoSNP-12	0.668 ± 0.050	0.771 ± 0.043	0.711 ± 0.045	0.761 ± 0.045	0.722 ± 0.046	0.863 ± 0.030
Infinium_Core	0.707 ± 0.061	0.791 ± 0.047	0.728 ± 0.049	0.776 ± 0.049	0.739 ± 0.051	0.871 ± 0.032
Infinium_OncoArray	0.747 ± 0.050	0.822 ± 0.039	0.755 ± 0.043	0.813 ± 0.040	0.775 ± 0.042	0.888 ± 0.028
PsychArray	0.739 ± 0.050	0.813 ± 0.040	0.747 ± 0.044	0.801 ± 0.041	0.763 ± 0.044	0.883 ± 0.028
Axiom_GW_ASI	0.771 ± 0.078	0.822 ± 0.064	0.775 ± 0.068	0.808 ± 0.067	0.783 ± 0.069	0.893 ± 0.048
Infinium_GSA	0.736 ± 0.049	0.828 ± 0.039	0.774 ± 0.042	0.834 ± 0.039	0.783 ± 0.041	0.903 ± 0.024
Axiom_GW_CHB	0.782 ± 0.037	0.835 ± 0.029	0.801 ± 0.028	0.819 ± 0.030	0.793 ± 0.030	0.915 ± 0.015
Axiom_JAPONICA	0.780 ± 0.039	0.848 ± 0.031	0.831 ± 0.032	0.838 ± 0.031	0.815 ± 0.032	0.933 ± 0.016
Axiom_GW_EUR	0.749 ± 0.087	0.823 ± 0.070	0.758 ± 0.075	0.826 ± 0.071	0.786 ± 0.075	0.881 ± 0.055
Infinium_Chinese	0.795 ± 0.038	0.850 ± 0.032	0.811 ± 0.036	0.841 ± 0.033	0.814 ± 0.035	0.921 ± 0.021
Infinium_JSA	0.748 ± 0.043	0.825 ± 0.033	0.799 ± 0.035	0.811 ± 0.034	0.783 ± 0.035	0.917 ± 0.018
Axiom_UKB	0.773 ± 0.032	0.864 ± 0.027	0.791 ± 0.030	0.880 ± 0.028	0.828 ± 0.029	0.913 ± 0.017
CytoSNP-850K	0.836 ± 0.037	0.865 ± 0.031	0.809 ± 0.035	0.855 ± 0.032	0.828 ± 0.034	0.920 ± 0.021
Axiom_PMRA	0.830 ± 0.030	0.860 ± 0.029	0.814 ± 0.033	0.849 ± 0.031	0.813 ± 0.031	0.922 ± 0.018
Axiom_PMDA	0.844 ± 0.022	0.874 ± 0.019	0.803 ± 0.021	0.867 ± 0.020	0.828 ± 0.023	0.919 ± 0.010
Affymetrix_6.0	0.831 ± 0.049	0.854 ± 0.038	0.793 ± 0.044	0.838 ± 0.041	0.813 ± 0.044	0.909 ± 0.027
OmniZhongHua	0.861 ± 0.033	0.882 ± 0.028	0.839 ± 0.033	0.872 ± 0.029	0.850 ± 0.031	0.935 ± 0.018
Multi-Ethnic_EUR_EAS_SAS	0.845 ± 0.039	0.880 ± 0.032	0.839 ± 0.038	0.874 ± 0.034	0.851 ± 0.036	0.934 ± 0.022
Multi-Ethnic_Global	0.861 ± 0.036	0.886 ± 0.031	0.843 ± 0.036	0.878 ± 0.032	0.855 ± 0.034	0.936 ± 0.021
Infinium_GDA	0.865 ± 0.033	0.889 ± 0.028	0.846 ± 0.033	0.882 ± 0.029	0.859 ± 0.031	0.938 ± 0.018
Axiom_GW_PanAFR	0.900 ± 0.027	0.885 ± 0.026	0.831 ± 0.029	0.869 ± 0.027	0.851 ± 0.028	0.932 ± 0.016
Infinium_Omni2.5	0.892 ± 0.031	0.897 ± 0.027	0.850 ± 0.031	0.891 ± 0.029	0.868 ± 0.029	0.941 ± 0.017
Infinium_Omni5	0.903 ± 0.028	0.914 ± 0.024	0.864 ± 0.028	0.918 ± 0.025	0.887 ± 0.026	0.950 ± 0.015

**Table 3:** Mean and standard deviation of imputation coverage (defined by the proportion of variants with $r^2 \ge 0.8$ over total number of variants in each chromosome) measured in 22 autosomes at the MAF bin of (0.01–0.5].

Array name	AFR	AMR	EAS	EUR	SAS	VNP
CytoSNP-12	0.403 ± 0.058	0.627 ± 0.062	0.588 ± 0.055	0.630 ± 0.060	0.573 ± 0.058	0.766 ± 0.051
Infinium_Core	0.478 ± 0.088	0.665 ± 0.069	0.616 ± 0.060	0.656 ± 0.064	0.604 ± 0.066	0.780 ± 0.049
Infinium_OncoArray	0.553 ± 0.076	0.721 ± 0.056	0.653 ± 0.052	0.714 ± 0.053	0.658 ± 0.055	0.812 ± 0.042
PsychArray	0.536 ± 0.074	0.701 ± 0.058	0.640 ± 0.053	0.691 ± 0.054	0.636 ± 0.056	0.801 ± 0.043
Axiom_GW_ASI	0.639 ± 0.115	0.734 ± 0.091	0.688 ± 0.083	0.716 ± 0.086	0.682 ± 0.087	0.827 ± 0.075
Infinium_GSA	0.514 ± 0.080	0.737 ± 0.059	0.672 ± 0.055	0.754 ± 0.056	0.669 ± 0.058	0.848 ± 0.040
Axiom_GW_CHB	0.626 ± 0.057	0.743 ± 0.038	0.710 ± 0.031	0.720 ± 0.035	0.683 ± 0.036	0.863 ± 0.021
Axiom_JAPONICA	0.617 ± 0.065	0.769 ± 0.044	0.764 ± 0.040	0.755 ± 0.042	0.722 ± 0.043	0.902 ± 0.027
Axiom_GW_EUR	0.596 ± 0.121	0.739 ± 0.099	0.665 ± 0.089	0.749 ± 0.094	0.689 ± 0.095	0.810 ± 0.084
Infinium_Chinese	0.652 ± 0.061	0.774 ± 0.043	0.732 ± 0.043	0.761 ± 0.042	0.722 ± 0.043	0.875 ± 0.032
Infinium_JSA	0.532 ± 0.067	0.721 ± 0.049	0.702 ± 0.046	0.703 ± 0.047	0.660 ± 0.048	0.874 ± 0.032
Axiom_UKB	0.587 ± 0.048	0.807 ± 0.035	0.706 ± 0.035	0.839 ± 0.034	0.750 ± 0.035	0.865 ± 0.025
CytoSNP-850K	0.746 ± 0.057	0.798 ± 0.040	0.728 ± 0.042	0.780 ± 0.039	0.741 ± 0.042	0.867 ± 0.031
Axiom_PMRA	0.734 ± 0.042	0.797 ± 0.037	0.743 ± 0.039	0.781 ± 0.038	0.725 ± 0.038	0.884 ± 0.027
Axiom_PMDA	0.772 ± 0.029	0.823 ± 0.023	0.724 ± 0.026	0.808 ± 0.024	0.746 ± 0.029	0.872 ± 0.018
Affymetrix_6.0	0.736 ± 0.082	0.777 ± 0.057	0.704 ± 0.056	0.751 ± 0.056	0.717 ± 0.059	0.845 ± 0.045
OmniZhongHua	0.802 ± 0.046	0.827 ± 0.035	0.770 ± 0.037	0.809 ± 0.035	0.777 ± 0.036	0.899 ± 0.027
Multi-Ethnic_EUR_EAS_SAS	0.769 ± 0.061	0.831 ± 0.041	0.774 ± 0.045	0.819 ± 0.042	0.785 ± 0.044	0.903 ± 0.032
Multi-Ethnic_Global	0.800 ± 0.056	0.838 ± 0.040	0.778 ± 0.043	0.823 ± 0.040	0.789 ± 0.043	0.906 ± 0.031
Infinium_GDA	0.807 ± 0.051	0.843 ± 0.036	0.783 ± 0.040	0.828 ± 0.037	0.794 ± 0.039	0.909 ± 0.028
Axiom_GW_PanAFR	0.870 ± 0.033	0.828 ± 0.030	0.759 ± 0.031	0.800 ± 0.031	0.776 ± 0.032	0.886 ± 0.023
Infinium_Omni2.5	0.856 ± 0.039	0.853 ± 0.033	0.784 ± 0.034	0.841 ± 0.034	0.803 ± 0.034	0.907 ± 0.025
Infinium_Omni5	0.872 ± 0.035	0.881 ± 0.029	0.802 ± 0.031	0.889 ± 0.029	0.834 ± 0.031	0.921 ± 0.022

### PGS performance

We evaluated PGS performance of these arrays based on two criteria: (i) Pearson’s correlation of PGS estimated by using imputed SNP array data compared to the PGS estimated by using WGS data—hereafter we refer as PGS correlation for short, (ii) absolute difference of percentile ranking (ADPR) between PGS generated by array-imputed and gold standard WGS. Both comparisons are set under various p-value cutoffs that allow us unbiased evaluate PGS performance of these arrays. In general, we found that PGS performance was highly concordant with imputation performance, i.e. SNP arrays with better imputation performance showed higher PGS correlation and less ADPR than the arrays with poor imputation performances.

**Figure 3:**
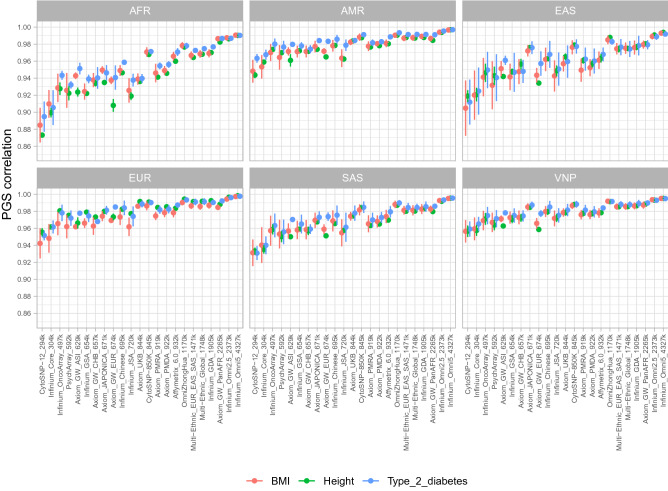
Correlations between PGS estimated from imputed genotyping data of 23 SNP arrays and PGS estimated from WGS in six different populations with three phenotypes including height, BMI, and type 2 diabetes. The dots and the vertical lines present the mean and standard deviation of PGS correlation at various p-value settings including 5e−08, 1e−07, 1e−06, 1e−05, 0.0001, 0.001, 0.01, 0.1, 0.2, 0.3, 0.5, and 1.

The summary results of Pearson’s correlation values of PGS from 23 genotyping SNP arrays for three different phenotypes are shown in Fig. 3 and in Tables S.7–9. In general, all examined arrays yielded high PGS correlations. Notably, the vast of majority PGS correlations ranged from 0.90 to 0.99, except for the two lowest density arrays (Infinium HumanCytoSNP-12 v2.1 and Infinium Core-24 v1.2) which had the lowest values. Interestingly, when optimal arrays for populations were used (the Axiom UK Biobank Array was used for the EUR population; and the Axiom Japonica Array NEO, Infinium OmniZhongHua v1.4 were used for EAS and VNP populations), the PGS correlations were higher than 0.97. The PGS correlation patterns were also highly concordant in all three evaluated traits with comparable performances. As expected, SNP arrays with larger sizes showed higher PGS correlations. The lowest performer was the Infinium HumanCytoSNP-12 v2.1 with a correlation of 0.8731 in the height phenotype in the AFR population while the highest performance was obtained by the Infinium Omni5 v1.2 with PGS correlation higher than 0.99 in all examined populations and traits. We also examined the deviation of PGS correlation in various p-value settings. The results showed that SNP array with lower PGS correlation had higher PGS correlation standard deviation than the high-performance arrays. A possible explanation for this observation is the PGS estimated from low imputation performance are more vulnerable to the random pruning process than the high imputation performance arrays^42^. Notably, we also observed higher standard deviations of PGS correlation in EAS than in other populations.

In agreement with imputation performance, SNP arrays optimized specifically for targeted populations showed superior PGS correlation in the targeted/closely related populations. For instance, Axiom Japonica Array NEO and Infinium OmniZhongHua v1.4 which were optimized for Japanese, and Chinese showed clear advantages in the populations of EAS, and VNP while Axiom UK Biobank Array yielded higher PGS correlation in the EUR population than the other size-comparable genotyping arrays. Taking height as a typical trait of interest, PGS correlations of the Japonica Array NEO were 0.9760, and 0.9847, while the Infinium OmniZhongHua v1.4 had 0.9879, and 0.9914 in EAS and VNP respectively. Interestingly, we observed that the Infinium CytoSNP-850K v1.2 was the array with superior PGS correlations in all populations, for all the three evaluated traits. For example, the PGS correlation for this array for height phenotype in AFR, AMR, EAS, EUR, SAS and VNP were 0.9679, 0.9876, 0.9789, 0.9908, 0.9844, 0.988, respectively.

**Figure 4:**
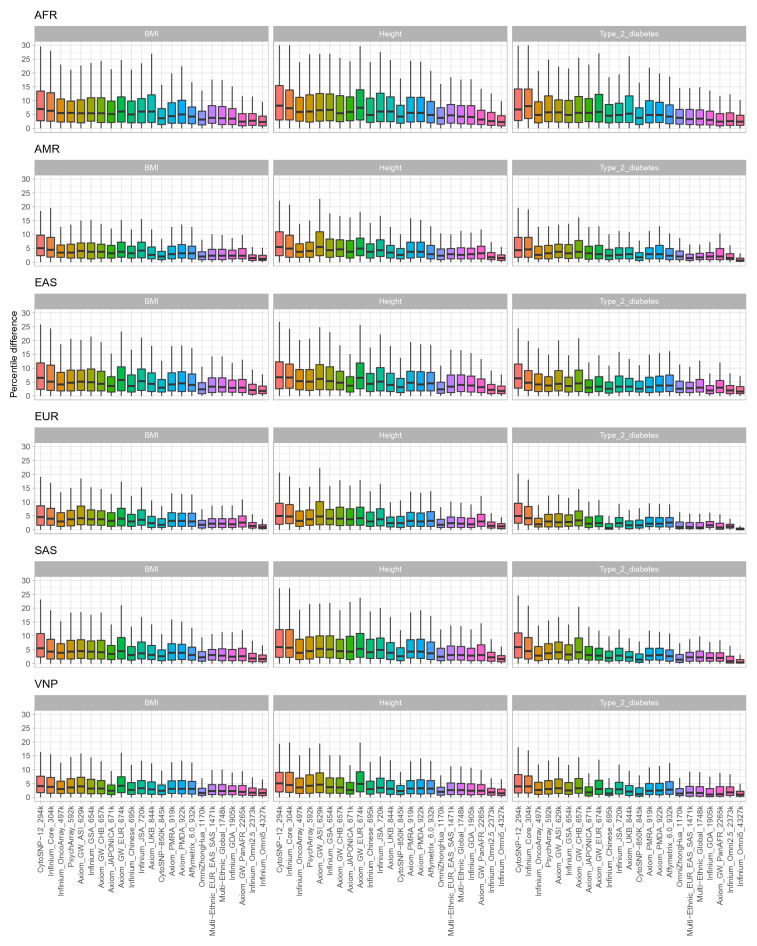
The absolute difference of percentile ranking between PGS estimated from imputed genotyping data of 23 SNP arrays and PGS estimated from WGS in six different populations. The figure shows results of three phenotypes including height, BMI, and type two diabetes with PRsice p-value setting of 5e−08.

Regarding the ADPR metric, the performance of arrays was in an agreement with the trend from comparing PGS correlation i.e. ADPRs were also affected by array sizes and optimization population. ADPR measurements in different PRSice-2 p-values settings are shown in Figs. 4, S.2–12; and reported in Tables S.10–21. Most of the arrays yielded mean ADPR less than 10 in all three traits. Exceptions were the AFR population with low-density arrays. The highest density array, i.e. Infinium Omni5 v1.2, had the highest performance with ADPR less than 4. Notably, ADPR varied by populations. Under-represented populations like AFR, and EAS tended to exhibit higher ADPRs than the others. Taking the p-value cutoff at 5e−8 for the height phenotype as an example (Fig. 4), Infinium Omni5 v1.2 obtained ADPR means of 3.8600, 2.4774, 2.8884, 1.9758, 2.8391, and 2.3699 in AFR, AMR, EAS, EUR, SAS and, VNP respectively. A consistent trend was also observed in other traits, with the lowest performance in AFR and the highest performance in EUR with ADPR means of 3.5974 and 1.8489 in BMI, and of 3.7206 and 1.6592 in type 2 diabetes. Similar to the other experiments, population specific arrays and the Infinium CytoSNP-850K v1.2 also illustrated their advantages when comparing the ADPR metric. The Axiom UK Biobank Array obtained good performance for the EUR population with ADPR means of 3.0584, 3.1714, and 2.2734 in height, BMI, and type 2 diabetes respectively. This trend was also observed in the cases of Axiom Japonica Array NEO, and Infinium OmniZhongHua v1.4 applied for the EAS and VNP populations. Regarding the Infinium CytoSNP-850K v1.2 array, good performances in all traits and populations were observed. Specifically, ADPR means of the height phenotype were 5.7141, 3.4914, 4.3753, 3.2501, 3.7638, 3.0267; for BMI at 4.9872, 2.5463, 4.1560, 2.6272, 3.5409, 3.1523; and for type 2 diabetes at 5.2000, 2.5762, 3.7687, 2.6066, 2.4707, 2.3812 in AFR, AMR, EAS, EUR, SAS and, VNP, respectively, all at the same p-value cutoff.

### Comparative analysis of real SNP array genotyping data and simulated genotyping data

**Figure 5:**
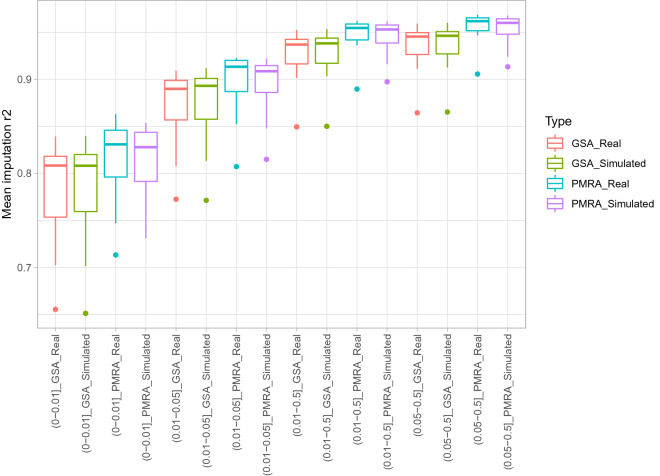
Mean imputation accuracy comparisons of simulated and real data of the Axiom Precision Medicine Research Array (PMRA) and the Infinium Global Screening Array v3.0 (GSA) genotyped of 24 VNP samples at various MAF bins measured in 22 autosomes.

We further utilized the availability of real genotyping data in the 1KVG dataset with 24 out of the 1008 samples also genotyped by the Axiom Precision Medicine Research Array and the Infinium Global Screening Array v3.0 to investigate how our simulated array data performed relative to the real array data. In brief, we generated pseudo genotyping data (termed simulated data) of 24 samples by extracting variants from WGS data that matched with the Axiom Precision Medicine Research Array and the Infinium Global Screening Array v3.0 manifests before excluding phasing information. Regarding real genotyping data, processed VCF (individual calling rate filtering at 97% and Hardy-Weinberg test filtering of 1e−6) files of 24 out of 1008 samples were obtained from https://genome.vinbigdata.org/ with no further filtering and quality control applied. We then applied the same pipeline to compare the imputation performance of the simulated genotyping data against the results obtained from the real genotyping data. In details, both simulated and real genotyping data were phased with SHAPEIT v4.1.3^34^, and imputed with Minimac4 v1.0.2^12^. Reference data for both phasing and imputation were the remaining 984 WGS samples. Finally, imputation performance of both simulated and real arrays were estimated as described in the “Imputation performance evaluation” section. As expected, the imputation accuracies of simulated and real data were highly concordant in both the two examined arrays as shown in Fig. 5 and Table 4. For example, mean and standard deviation of imputation accuracies of simulated Axiom Precision Medicine Research Array were 0.8144 ± 0.0359, 0.8971 ± 0.0273, 0.9459 ± 0.016, 0.9542 ± 0.014; and real data were 0.8173 ± 0.0379, 0.9013 ± 0.0285, 0.9492 ± 0.0158, 0.9573 ± 0.0135 in four MAF bins of (0–0.01], (0.01–0.05], (0.01–0.5], and (0.05–0.5], respectively. Furthermore, relative performances between the Axiom Precision Medicine Research Array and the Infinium Global Screening Array v3.0 were equivalent in simulated and real data. These results indicated the robustness of our simulation approach in imputation performance evaluation of genotyping arrays in reality.

**Table 4:** Mean and the standard deviation of imputation accuracies of simulated and real data of the Axiom Precision Medicine Research Array (PMRA) and the Infinium Global Screening Array v3.0 (GSA) of 24 VNP samples at various MAF bins measured in 22 autosomes.

MAF range	Simulated PMRA	Real PMRA	Simulated GSA	Real GSA
(0–0.01]	0.814 ± 0.036	0.817 ± 0.038	0.786 ± 0.049	0.785 ± 0.049
(0.01–0.05]	0.897 ± 0.027	0.901 ± 0.029	0.877 ± 0.035	0.875 ± 0.035
(0.01–0.5]	0.946 ± 0.016	0.949 ± 0.016	0.929 ± 0.023	0.928 ± 0.023
(0.05–0.5]	0.954 ± 0.014	0.957 ± 0.014	0.938 ± 0.021	0.937 ± 0.021

## Discussions and conclusions

Even in a booming time of next-generation sequencing technologies, current big genotyping projects are still using SNP arrays as the work-horse for generating valuable data, especially for bio-bank scale projects^2,25,26^. Moreover, genotyping by SNP arrays produce the exact information typically required for PGS analysis, which is based on summarizing effect sizes from individual SNPs. A promising application of genomic research that is gaining increasing interest recently across the healthcare system, and direct-to-consumer genomic services based on polygenic scoring like 23andMe^5,46^. SNP arrays are clearly economical in data generation and analysis, an important factor in designing projects with large sample sizes and/or limited budget. Given that there are many available human genotyping arrays optimized for various purposes, a comprehensive guideline for choosing the most suitable SNP arrays in multiple ancestry groups is still lacking. To address this gap, we have introduced a systematic approach to assess a large range of SNP arrays across multiple datasets. We performed imputation and PGS performance assessments for 23 human available genotyping arrays in six ancestry groups using both public and in-house datasets by various metrics. By comparing the relative performance of SNP arrays to WGS with 4 metrics including imputation accuracy, imputation coverage, PGS correlation, and ADPR, we discovered important insights that can be used to suggest suitable arrays for genotyping-based studies on a specific population, especially under-represented populations.

Overall, we found that all 23 assessed arrays had high performances in both imputation and PGS. These commercial arrays differ markedly in designs, i.e. the number of markers on the arrays and targeted ancestry groups that would cause performance deviations. An important finding in our analysis was that in order to obtain high imputation performances, the choice of an array is not necessarily about getting higher density, but small to moderately-sized arrays (approximately 650k–850k tag SNPs), accompanied by well optimization for the targeted population could also produce high imputation and PGS performances. For example, the Japonica Array NEO, and the UK Biobank Array showed the highest performance when compared with other arrays with the same sizes for EAS, and EUR populations respectively. This indicates that using customized, small-size SNP arrays at the population-specific level can be a cost-effective genotyping solution without losing PGS performance^22,47^. We also observed that there were no specific arrays with moderate sizes that had superior imputation performances in AFR, and SAS, suggesting the need for genotyping arrays optimized for these populations. PGS performances were concordant to imputation performances in general. However, CytoSNP-850K v1.2 was an interesting array that showed superior PGS performances in all populations. This superior performance may be explained by the enrichment of cytogenetic regions in the design of the Infinium CytoSNP-850K v1.2 array^48^. The analyses also showed that underrepresented populations such as AFR, and SAS exhibited lower PGS performances (and ADPRs tended to be higher in AFR, and SAS) than other well-studied populations regardless of sample sizes were not significantly different in these populations. A possible explanation for these lower performances is due to the use of meta-analysis GWAS summary statistics in the current study. The strong bias in GWAS participants toward populations of European descent could be a reason for lower PGS in other populations as described previously^43,44,49,50^. In addition, PGS performances of small-sized arrays were significant lower in AFR which was possibly due to the higher number of genetic variations in this population^1^.

Notably, PGS constructed from imputed genotypes were very high in comparison with the original WGS PGS. The majority PGS correlations ranged from 0.90 to 0.99. In cases of optimal arrays for targeted populations in used (UK Biobank Array was used for the EUR population, Japonica Array NEO was used for EAS and VNP populations), the PGS correlation to WGS was higher than 0.97. In addition, PGS ranking differences between WGS and imputed array genotypes were not high with the majority of differences were under 5 percentile when optimal arrays were used. The possible reason for this observation was that current GWAS summary statistics were mostly generated by imputed array genotypes^43,44^ that were limited to detect rare associated markers. This indicates that using WGS for PGS analysis does not provide significant improvement in term of disease risk stratification at this time although this trend can change in the future when GWAS summary statistics at higher resolution become widely available^51^.

Finally, to make this analysis capability available to broad audiences, we have developed a web tool that provides interactive analyses of SNP array contents and performances. As researchers may be interested in specific variants or regions, the tool aimed to support researchers to analyze SNP array contents and imputation performance based on population and genomic regions of interest. We hope this tool could facilitate researchers in designing their SNP array-based studies.

## Supplementary Information

Supplementary Figures.

Supplementary Tables.

## Data Availability

The 1KGP-NYGC datasets are freely available at IGSR data portal (https://www.internationalgenome.org). The 1KVG WGS and genotyping datasets are available under agreement at MASH data portal (https://genome.vinbigdata.org/). Data and source codes to generate figures of this study are available at: https://github.com/datngu/SNP_array_comparison. SNP array analyzing tool is available online at: https://genome.vinbigdata.org/tools/saa/. SNP-wise imputation performance estimation based on 1KGP-NYGC data are freely available at: https://zenodo.org/record/6548098. SNP-wise imputation performance estimation based on 1KVG data are available and can be supplied under ethical policy agreement.
