# Supplementary material for: A comprehensive evaluation of polygenic score and genotype imputation performances of human SNP arrays in diverse populations
Source: Sci Rep. 2022 Oct 20;12:17556. doi: 10.1038/s41598-022-22215-y (PMC9585077; doi:10.1038/s41598-022-22215-y)
Supplement: Supplementary file 1 — Supplementary Figures. [file 41598_2022_22215_MOESM1_ESM.pdf]

A comprehensive evaluation of polygenic score and genotype imputation performances of human SNP arrays in diverse populations
[Supplementary documents]

By Dat Thanh Nguyen, et al

July 4, 2022

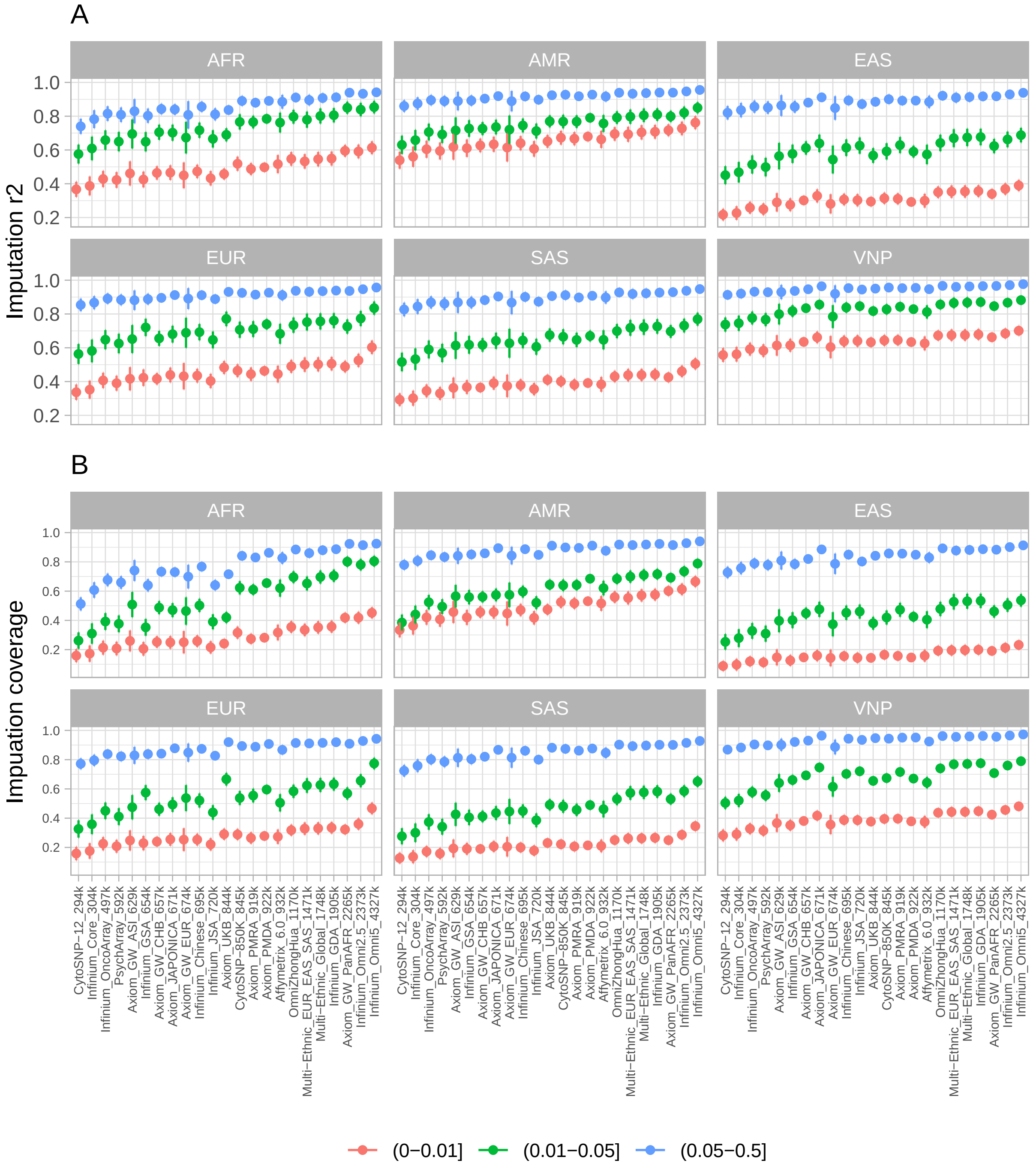

Figure S. 1: A. Mean imputation r^2, and B. Imputation coverage across 22 autosomes of 23 SNP arrays in three MAF bins including (0-0.01], (0.01-0.05], and (0.05-0.5]. The dots and the vertical lines present the mean and the standard deviation of imputation/ coverage values in 22 autosomes respectively.

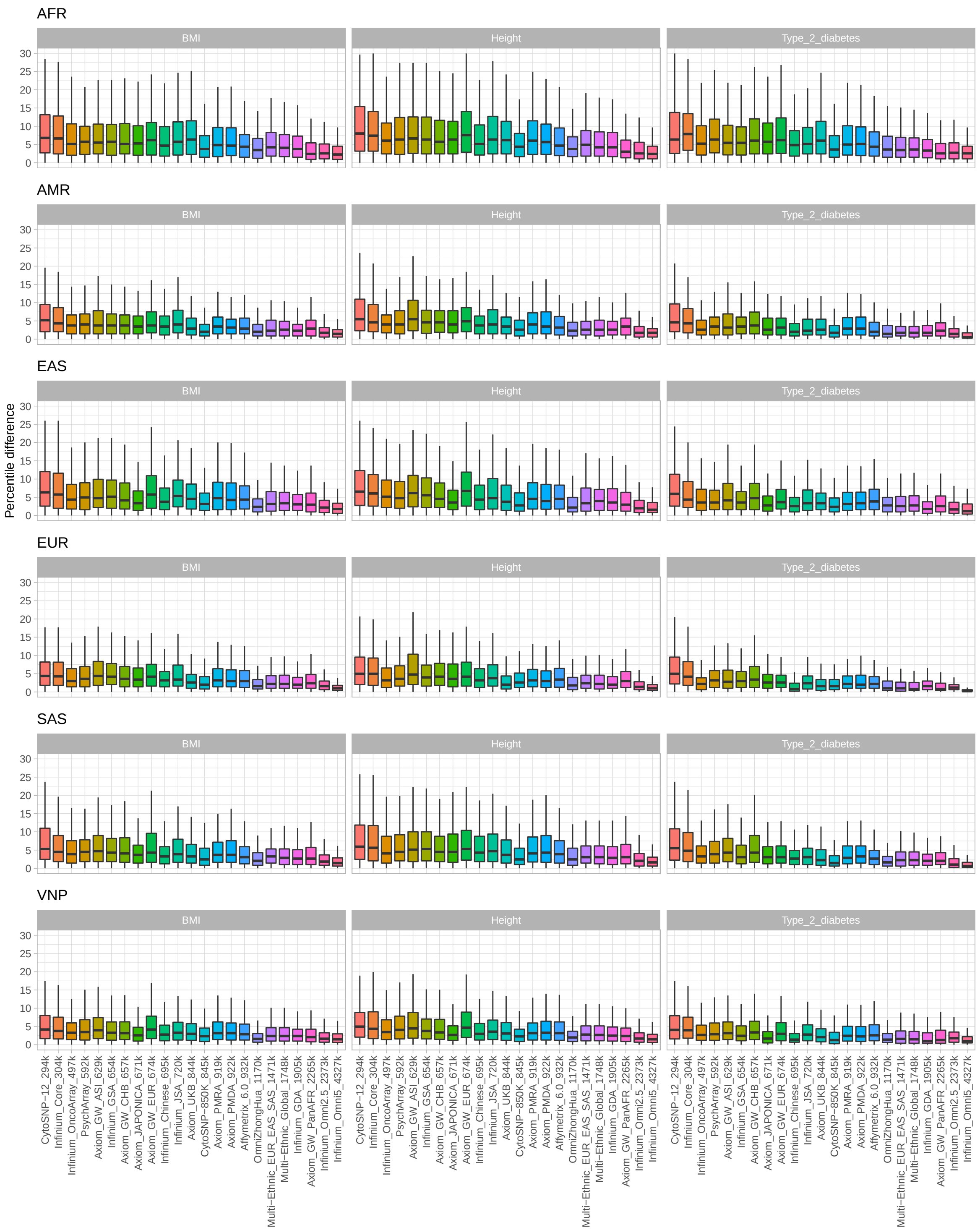

Figure S. 2: Absolute difference of percentile ranking between PGSs estimated from imputed genotyping data of 23 SNP arrays and PGSs estimated from WGS in 6 different populations. The figure shows results of 3 phenotypes including height, BMI, and type 2 diabetes with PRsice p-value setting of 1e-07.

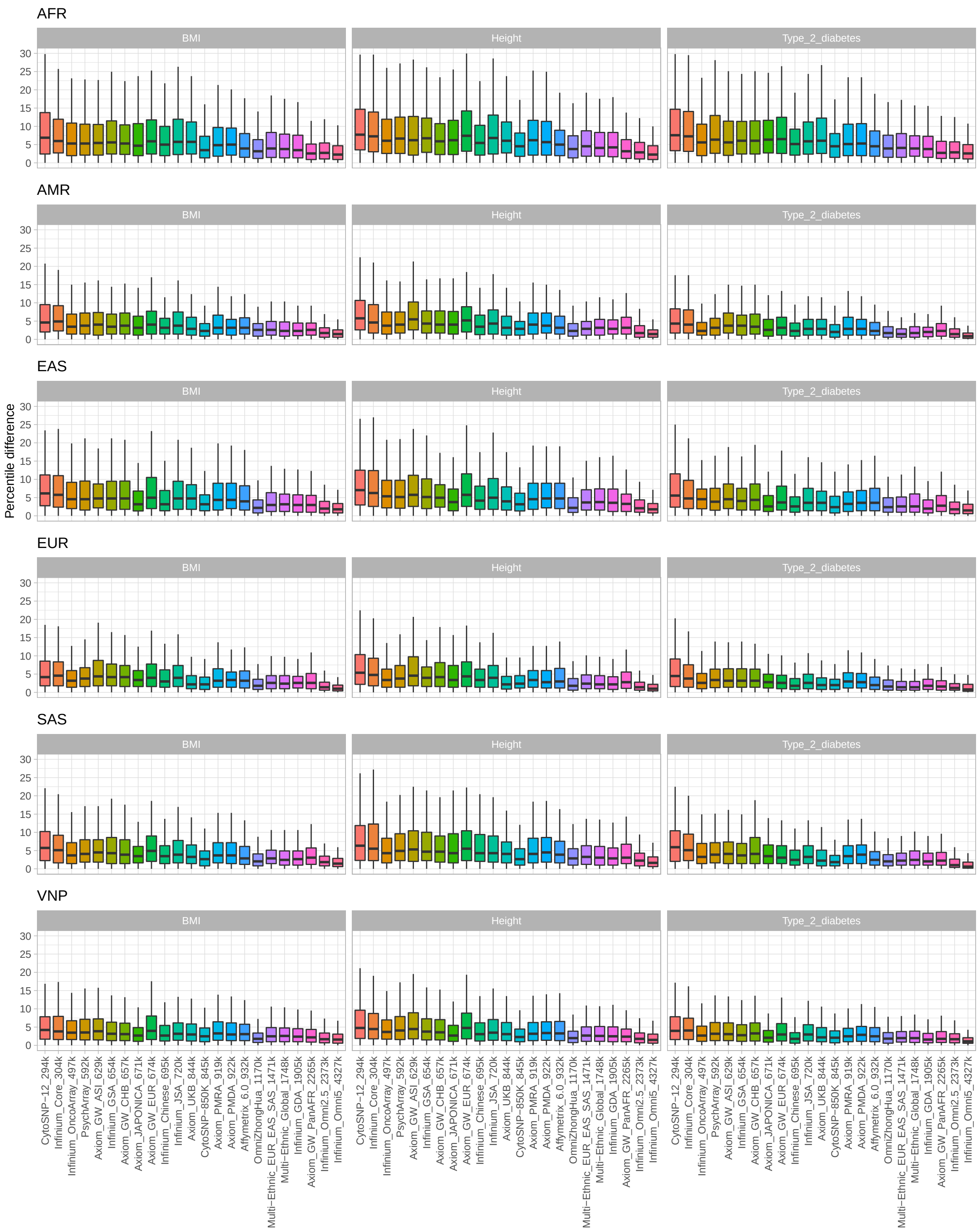

Figure S. 3: Absolute difference of percentile ranking between PGSs estimated from imputed genotyping data of 23 SNP arrays and PGSs estimated from WGS in 6 different populations. The figure shows results of 3 phenotypes including height, BMI, and type 2 diabetes with PRsice p-value setting of 1e-06.

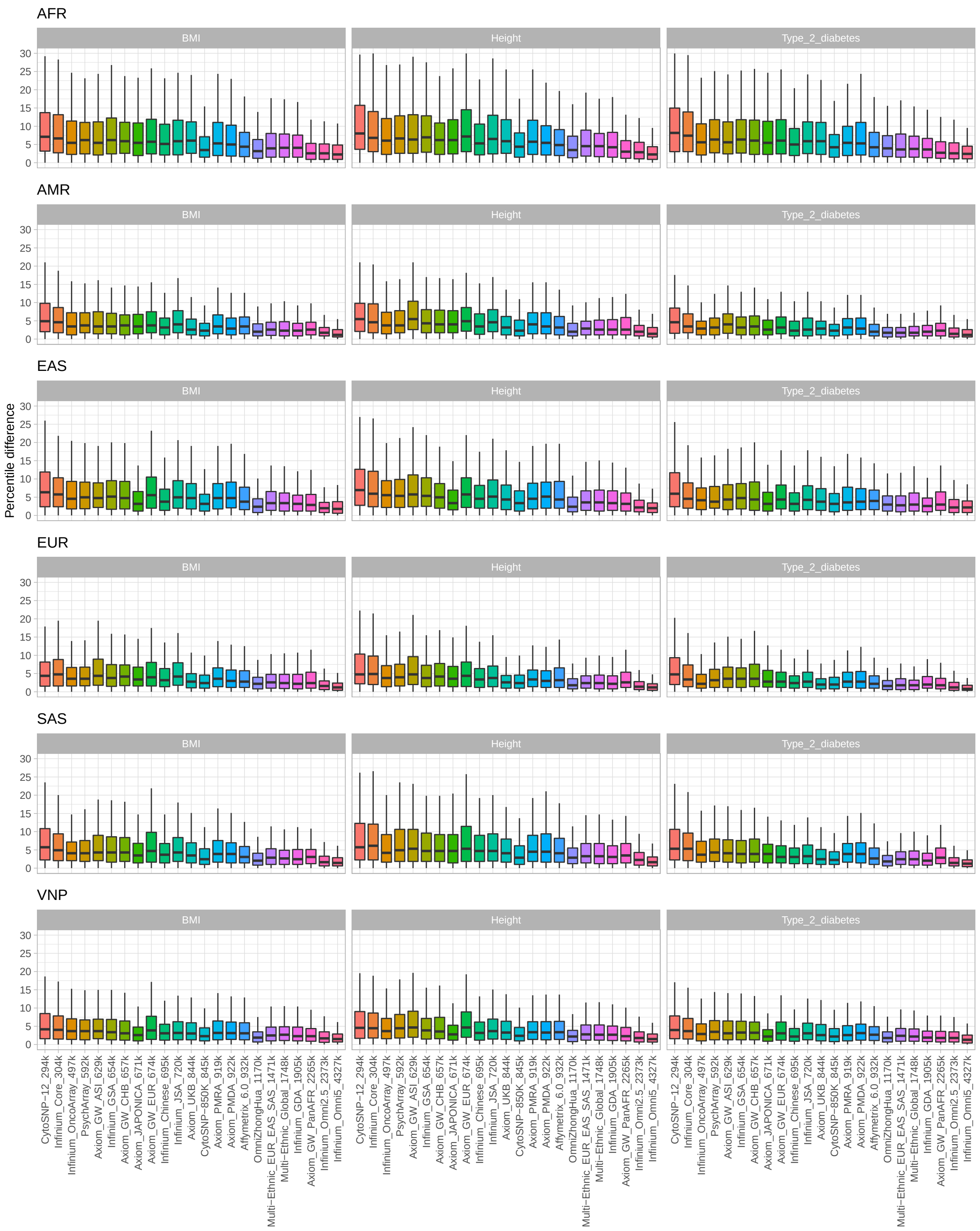

Figure S. 4: Absolute difference of percentile ranking between PGSs estimated from imputed genotyping data of 23 SNP arrays and PGSs estimated from WGS in 6 different populations. The figure shows results of 3 phenotypes including height, BMI, and type 2 diabetes with PRsice p-value setting of 1e-05.

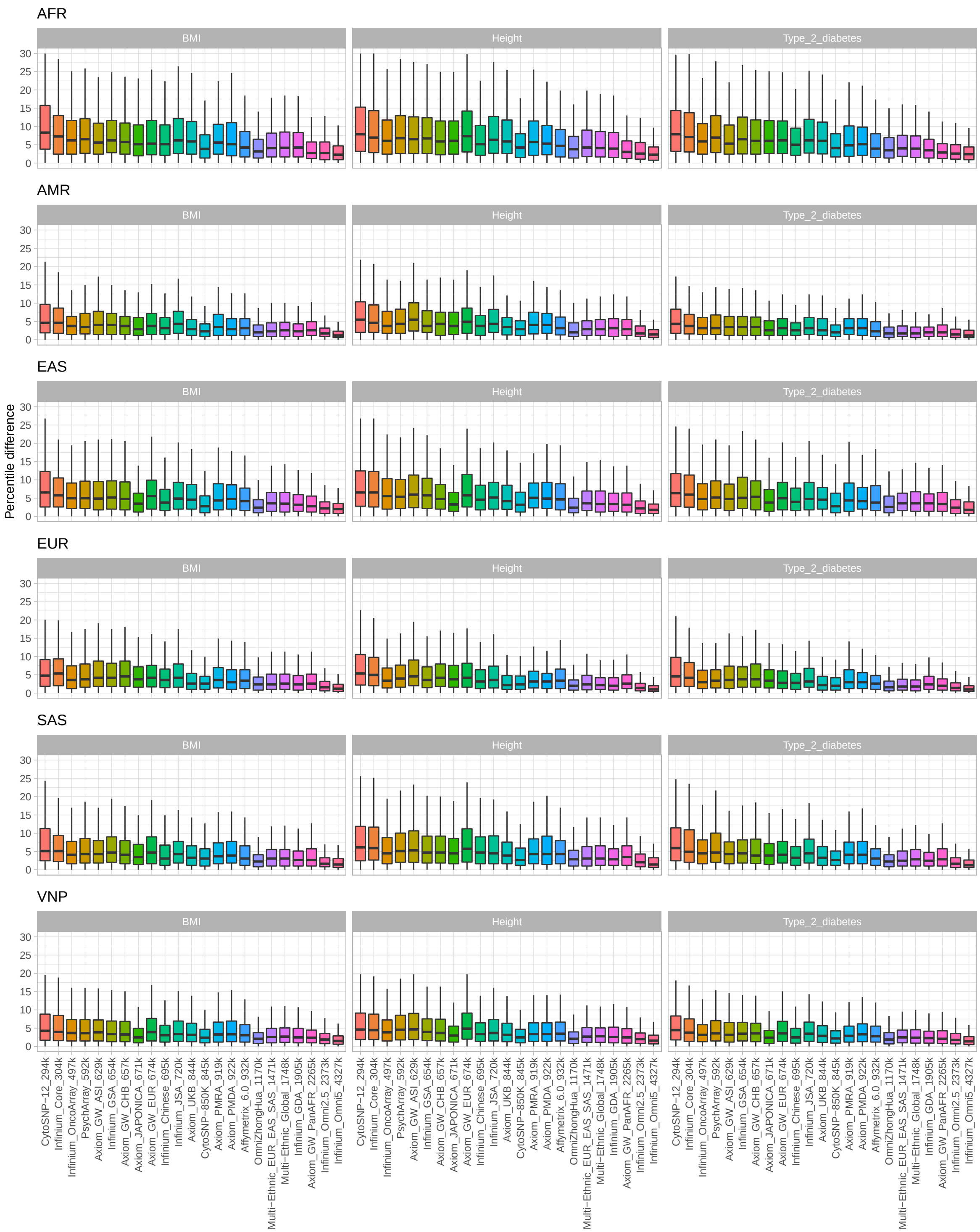

Figure S. 5: Absolute difference of percentile ranking between PGSs estimated from imputed genotyping data of 23 SNP arrays and PGSs estimated from WGS in 6 different populations. The figure shows results of 3 phenotypes including height, BMI, and type 2 diabetes with PRsice p-value setting of 0.0001.

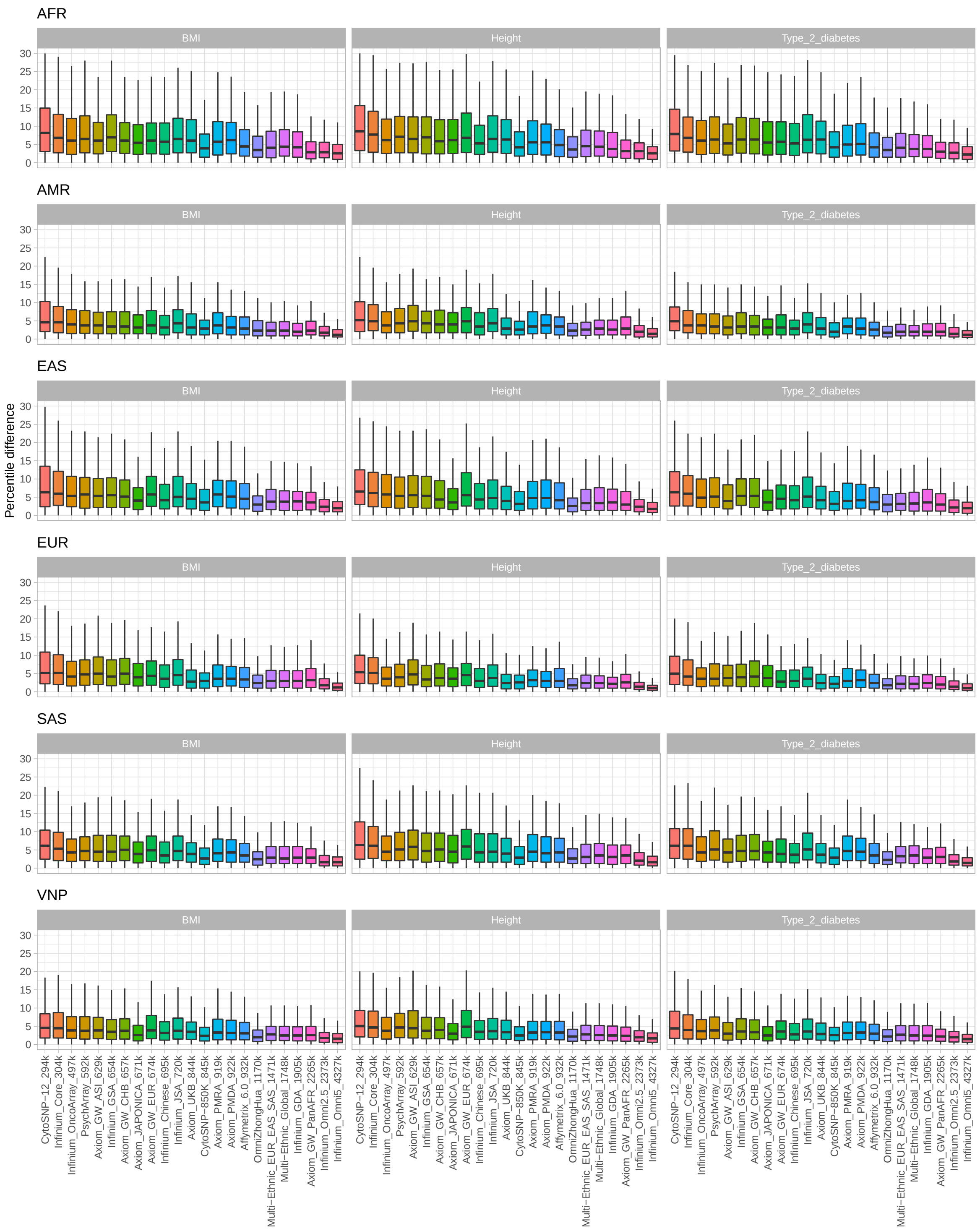

Figure S. 6: Absolute difference of percentile ranking between PGSs estimated from imputed genotyping data of 23 SNP arrays and PGSs estimated from WGS in 6 different populations. The figure shows results of 3 phenotypes including height, BMI, and type 2 diabetes with PRsice p-value setting of 0.001.

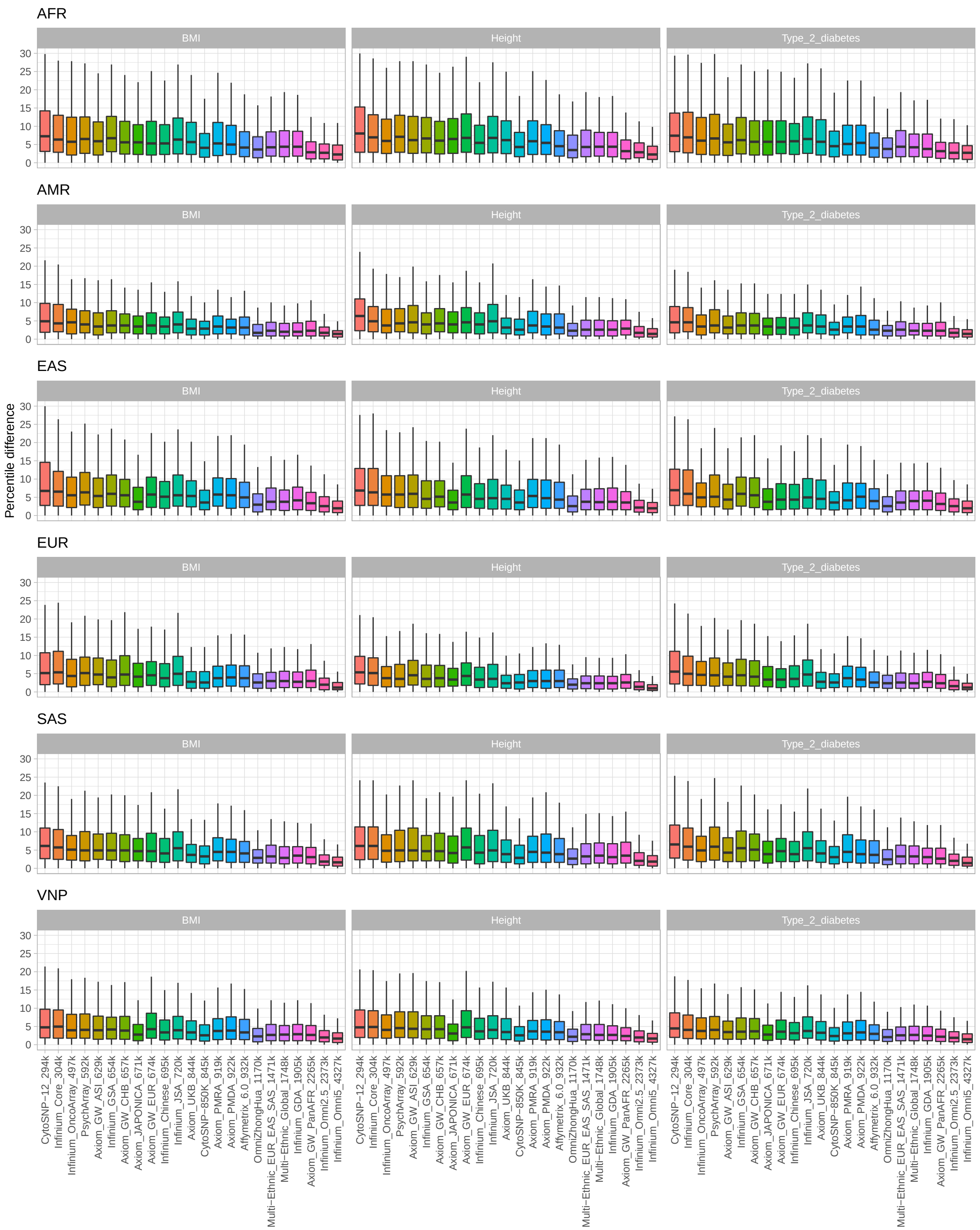

Figure S. 7: Absolute difference of percentile ranking between PGSs estimated from imputed genotyping data of 23 SNP arrays and PGSs estimated from WGS in 6 different populations. The figure shows results of 3 phenotypes including height, BMI, and type 2 diabetes with PRsice p-value setting of 0.01.

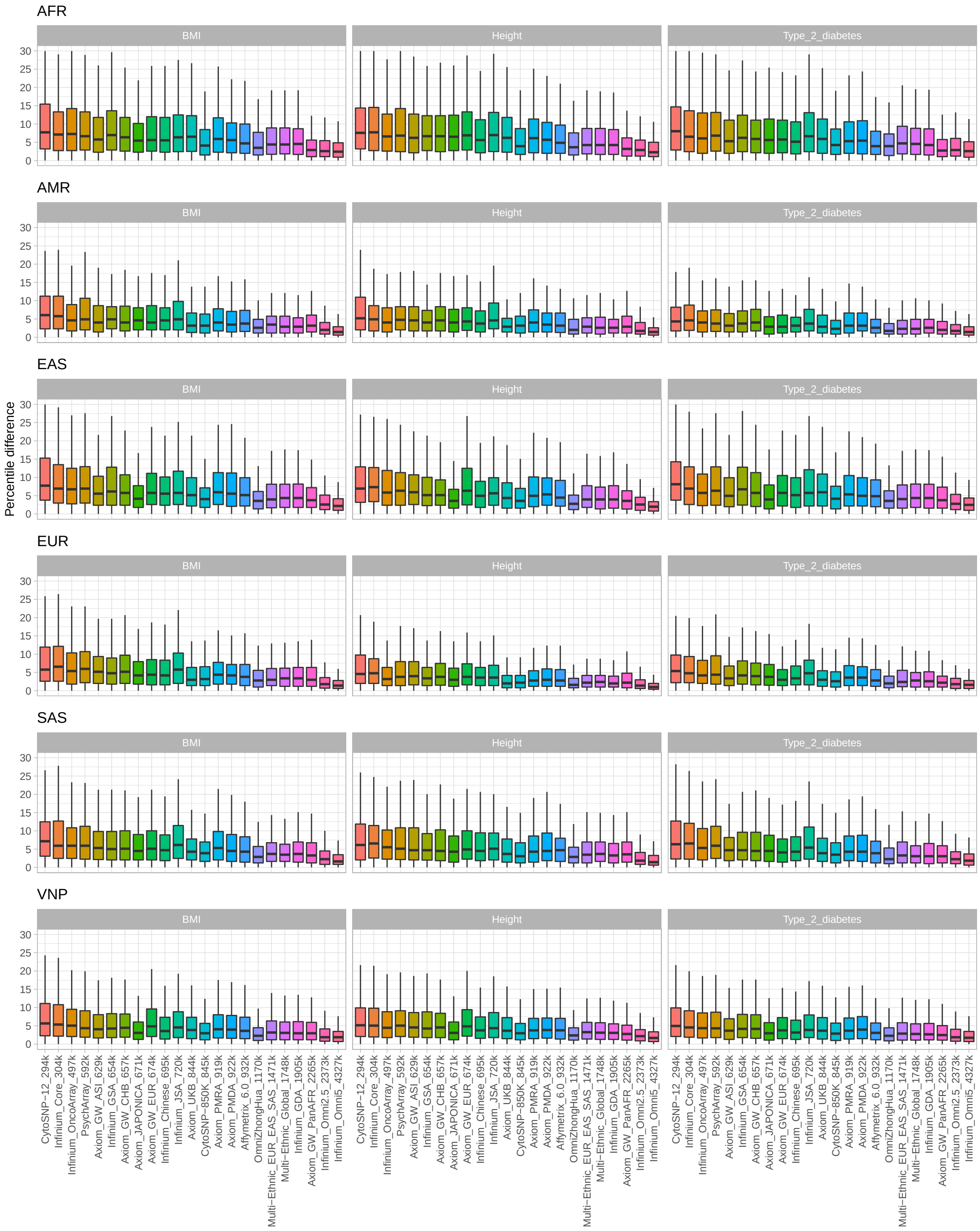

Figure S. 8: Absolute difference of percentile ranking between PGSs estimated from imputed genotyping data of 23 SNP arrays and PGSs estimated from WGS in 6 different populations. The figure shows results of 3 phenotypes including height, BMI, and type 2 diabetes with PRsice p-value setting of 0.1.

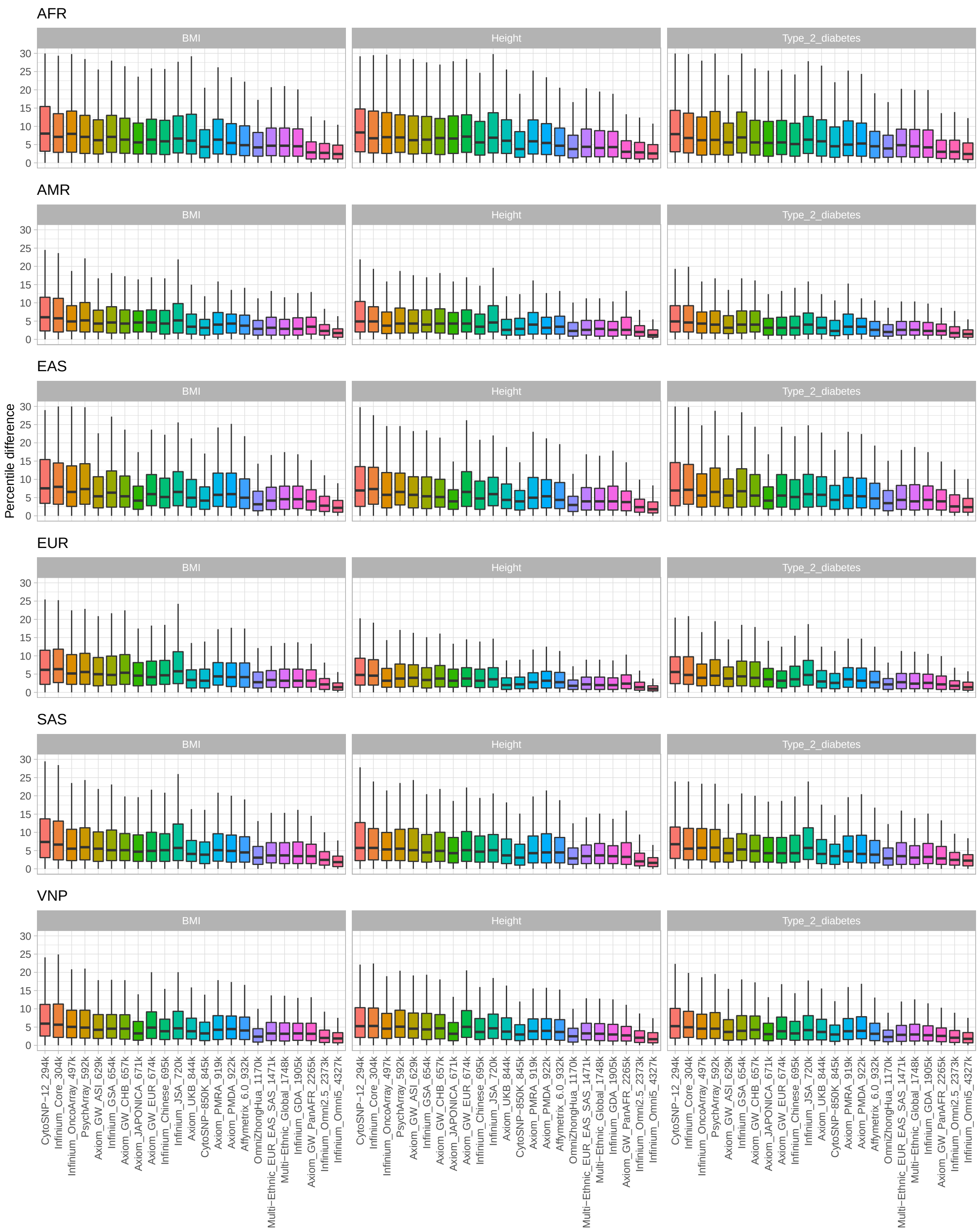

Figure S. 9: Absolute difference of percentile ranking between PGSs estimated from imputed genotyping data of 23 SNP arrays and PGSs estimated from WGS in 6 different populations. The figure shows results of 3 phenotypes including height, BMI, and type 2 diabetes with PRsice p-value setting of 0.2.

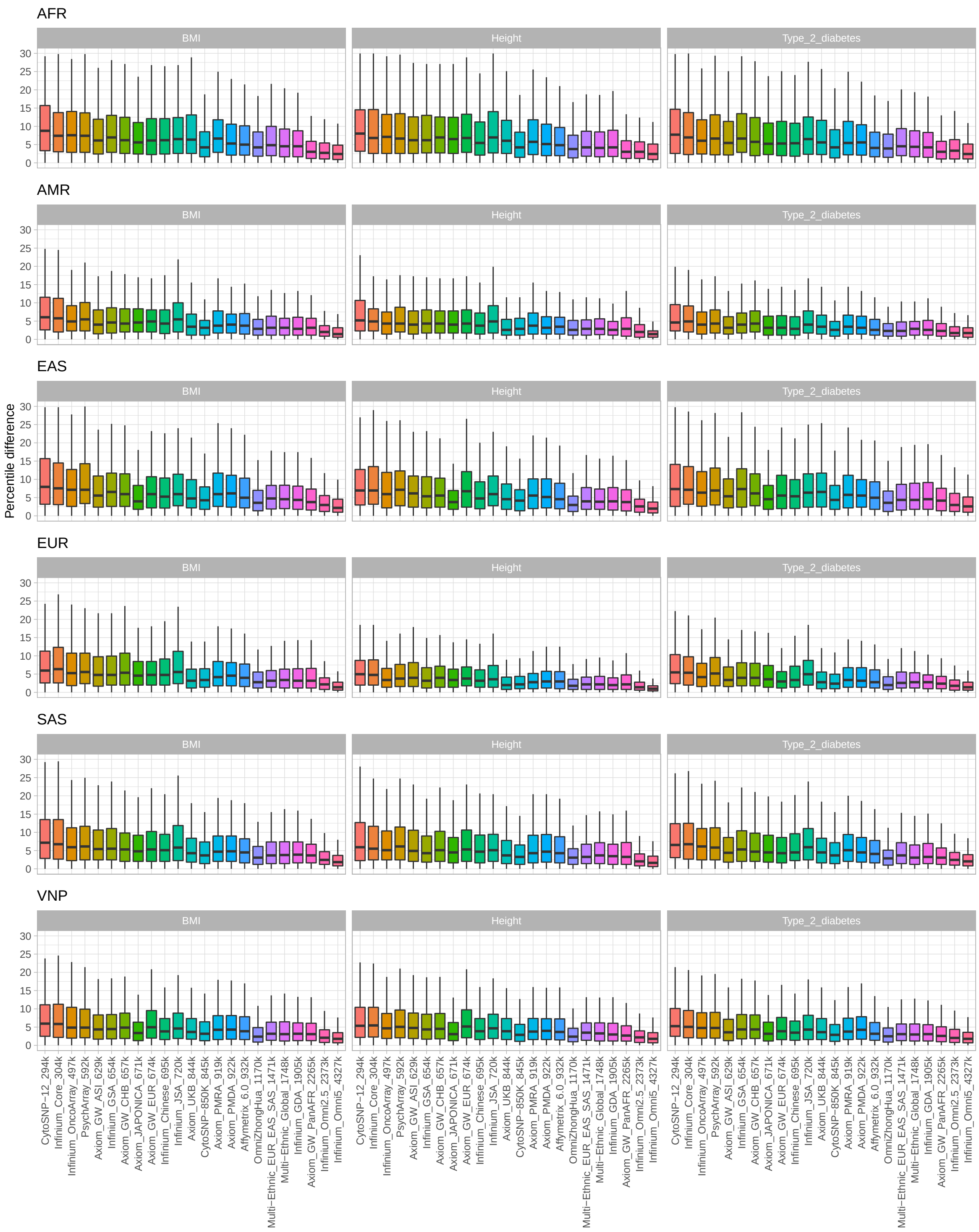

Figure S. 10: Absolute difference of percentile ranking between PGSs estimated from imputed genotyping data of 23 SNP arrays and PGSs estimated from WGS in 6 different populations. The figure shows results of 3 phenotypes including height, BMI, and type 2 diabetes with PRsice p-value setting of 0.3.

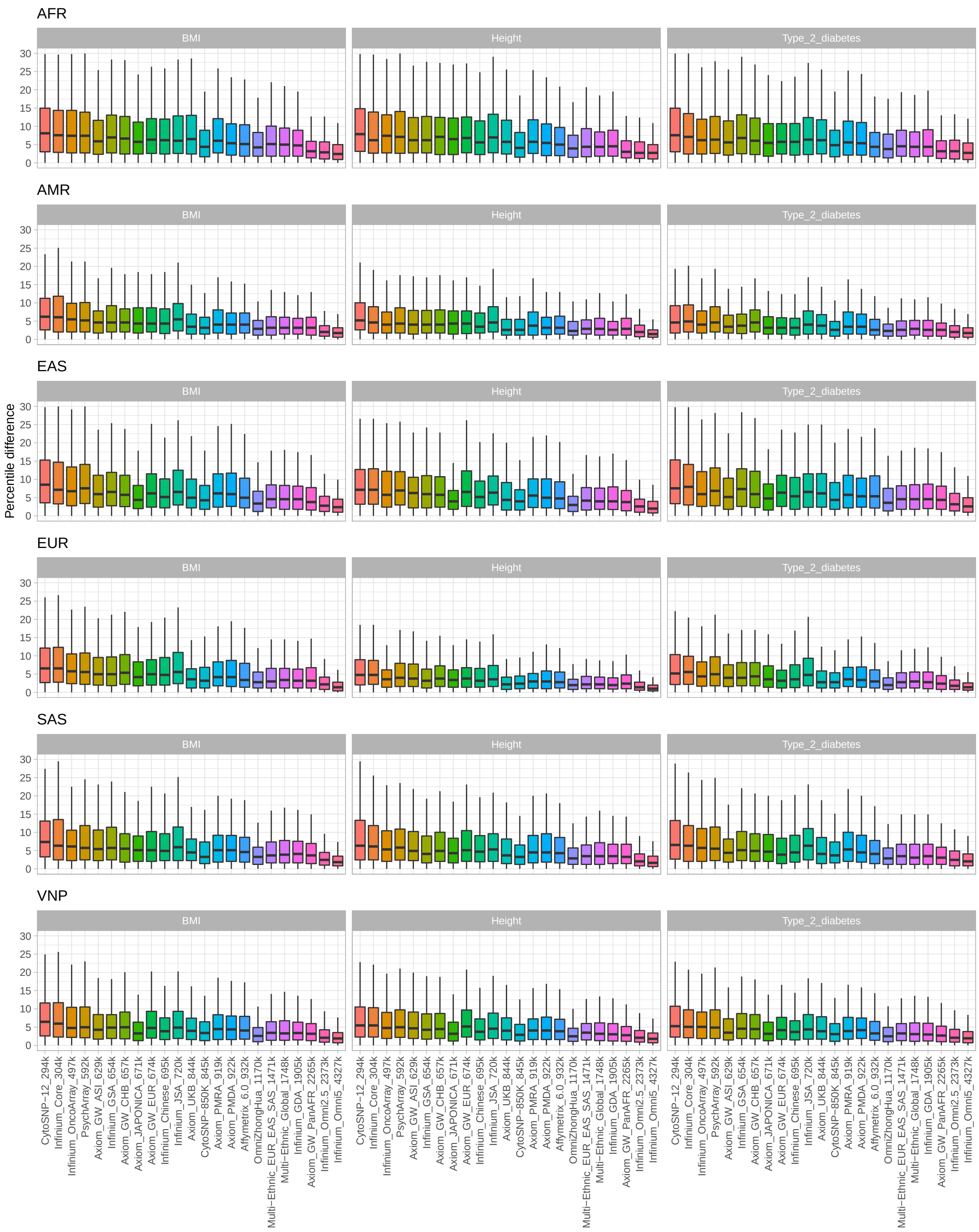

Figure S. 11: Absolute difference of percentile ranking between PGSs estimated from imputed genotyping data of 23 SNP arrays and PGSs estimated from WGS in 6 different populations. The figure shows results of 3 phenotypes including height, BMI, and type 2 diabetes with PRsice p-value setting of 0.5.

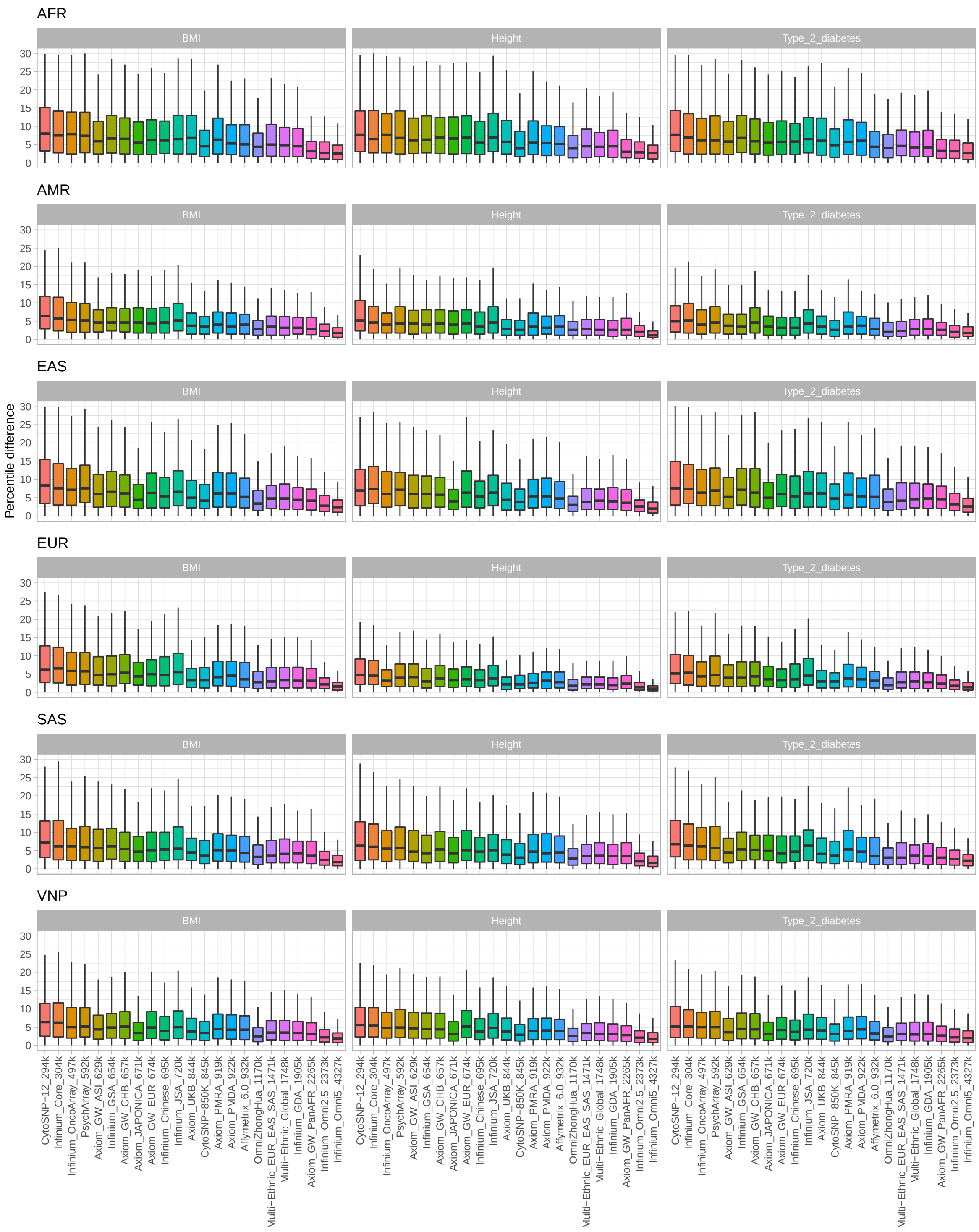

Figure S. 12: Absolute difference of percentile ranking between PGSs estimated from imputed genotyping data of 23 SNP arrays and PGSs estimated from WGS in 6 different populations. The figure shows results of 3 phenotypes including height, BMI, and type 2 diabetes with PRsice p-value setting of 1.
