# Supplementary material for: A comprehensive evaluation of polygenic score and genotype imputation performances of human SNP arrays in diverse populations
Source: Sci Rep. 2022 Oct 20;12:17556. doi: 10.1038/s41598-022-22215-y (PMC9585077; doi:10.1038/s41598-022-22215-y)
Supplement: Supplementary file 2 — Supplementary Tables. [file 41598_2022_22215_MOESM2_ESM.pdf]

Table S. 1 Mean and the standard deviation of imputation accuracy r^2 measured in 22 autosomes at the MAF bin of (0-0.01].

Array name	AFR	AMR	EAS	EUR	SAS	VNP
CytoSNP-12	0.3671±0.0451	0.5394±0.0503	0.2172±0.035	0.3369±0.0459	0.2924±0.0379	0.557±0.0409
Infinium_Core	0.3873±0.056	0.5603±0.0601	0.2267±0.0403	0.352±0.0533	0.3014±0.0449	0.5617±0.0437
Infinium_OncoArray	0.4283±0.0481	0.6045±0.0507	0.2583±0.0359	0.4066±0.0457	0.3447±0.0386	0.5919±0.0384
PsychArray	0.4225±0.0468	0.5941±0.0504	0.2492±0.0355	0.3901±0.0445	0.3298±0.0385	0.5827±0.0382
Axiom_GW_ASI	0.4612±0.0716	0.6179±0.0754	0.2899±0.0548	0.4165±0.0689	0.3626±0.0613	0.6138±0.0607
Infinium_GSA	0.4251±0.0469	0.6104±0.0518	0.2757±0.0383	0.4226±0.0492	0.3674±0.0414	0.6148±0.0385
Axiom_GW_CHB	0.4641±0.0401	0.627±0.0407	0.3016±0.0272	0.4162±0.0348	0.3644±0.029	0.6348±0.0261
Axiom_JAPONICA	0.4659±0.0436	0.6337±0.0451	0.3282±0.0392	0.439±0.044	0.3905±0.0389	0.6615±0.035
Axiom_GW_EUR	0.4498±0.0765	0.6143±0.0829	0.281±0.057	0.4315±0.078	0.3742±0.0668	0.604±0.0659
Infinium_Chinese	0.473±0.0401	0.6403±0.0425	0.307±0.0351	0.4348±0.041	0.3791±0.0357	0.6379±0.0352
Infinium_JSA	0.4325±0.0429	0.6067±0.0466	0.3027±0.0368	0.4035±0.0425	0.3552±0.0368	0.6402±0.0354
Axiom_UKB	0.4578±0.0334	0.6512±0.0383	0.2945±0.0291	0.4831±0.039	0.4101±0.032	0.6328±0.0297
CytoSNP-850K	0.5186±0.042	0.6726±0.0412	0.3139±0.0342	0.4641±0.0386	0.4008±0.0339	0.6433±0.0334
Axiom_PMRA	0.4873±0.0359	0.6667±0.0395	0.3109±0.0336	0.4439±0.0399	0.3813±0.0344	0.6451±0.0326
Axiom_PMDA	0.4974±0.0271	0.6793±0.0292	0.2924±0.0237	0.463±0.029	0.3913±0.0262	0.6339±0.0239
Affymetrix_6.0	0.5164±0.0538	0.6628±0.051	0.2996±0.0412	0.4443±0.0498	0.3835±0.0438	0.6258±0.0415
OmniZhongHua	0.5473±0.0406	0.6949±0.0406	0.35±0.0352	0.49±0.0387	0.4301±0.0344	0.6727±0.0313
Multi-Ethnic_EUR_EAS_SAS	0.5325±0.0431	0.6928±0.0446	0.3532±0.0373	0.5003±0.043	0.4381±0.0372	0.6745±0.0348
Multi-Ethnic_Global	0.5455±0.0429	0.7044±0.0427	0.3542±0.0369	0.5019±0.042	0.4391±0.0365	0.6754±0.0348
Infinium_GDA	0.5493±0.0417	0.7077±0.0411	0.3564±0.0357	0.5057±0.0403	0.4419±0.0355	0.6789±0.0334
Axiom_GW_PanAFR	0.595±0.0356	0.7168±0.0359	0.3398±0.0306	0.4886±0.0349	0.425±0.0306	0.6622±0.0285
Infinium_Omni2.5	0.5911±0.0411	0.7277±0.0405	0.3689±0.0348	0.5253±0.04	0.4603±0.035	0.6845±0.0308
Infinium_Omni5	0.6132±0.0385	0.7617±0.0389	0.3898±0.0336	0.6028±0.0404	0.5053±0.0345	0.7008±0.0296

Table S. 2 Mean and the standard deviation of imputation accuracy r^2 measured in 22 autosomes at the MAF bin of (0.01-0.05].

Array name	AFR	AMR	EAS	EUR	SAS	VNP
CytoSNP-12	0.5758±0.056	0.6304±0.0543	0.4508±0.0532	0.5633±0.059	0.5158±0.055	0.7376±0.0419
Infinium_Core	0.609±0.0698	0.658±0.0607	0.4682±0.0609	0.5812±0.0674	0.5326±0.0637	0.7453±0.0452
Infinium_OncoArray	0.6584±0.0576	0.7062±0.0497	0.5141±0.054	0.6478±0.0566	0.5898±0.053	0.7761±0.0385
PsychArray	0.6496±0.0573	0.6919±0.0507	0.4988±0.0549	0.6252±0.0578	0.5691±0.0545	0.7665±0.039
Axiom_GW_ASI	0.6949±0.0861	0.7157±0.0773	0.5637±0.0784	0.6515±0.083	0.6136±0.0793	0.799±0.0612
Infinium_GSA	0.6487±0.0573	0.7271±0.0492	0.5773±0.0544	0.7204±0.0514	0.6174±0.0528	0.8175±0.035
Axiom_GW_CHB	0.7051±0.0448	0.7264±0.0387	0.612±0.0397	0.6554±0.0442	0.6169±0.0403	0.8343±0.0238
Axiom_JAPONICA	0.7023±0.0477	0.7358±0.0436	0.639±0.0521	0.6805±0.0499	0.6412±0.0479	0.8555±0.0293
Axiom_GW_EUR	0.6731±0.0937	0.7202±0.0838	0.5433±0.0825	0.6889±0.0888	0.6267±0.0856	0.7845±0.0677
Infinium_Chinese	0.7169±0.0456	0.7451±0.0413	0.6134±0.0494	0.6923±0.0483	0.6429±0.0457	0.8381±0.0312
Infinium_JSA	0.6658±0.0513	0.7121±0.0444	0.6259±0.0483	0.6465±0.0497	0.6059±0.0475	0.8463±0.0288
Axiom_UKB	0.6898±0.0389	0.7695±0.036	0.5674±0.0439	0.7708±0.0434	0.6751±0.0393	0.8171±0.0279
CytoSNP-850K	0.7663±0.0454	0.7682±0.0403	0.5918±0.0481	0.7067±0.0478	0.6651±0.0447	0.8275±0.0312
Axiom_PMRA	0.7654±0.0377	0.7678±0.0374	0.629±0.0477	0.7104±0.0471	0.6466±0.0425	0.8429±0.0287
Axiom_PMDA	0.7843±0.0278	0.7903±0.0262	0.5902±0.0354	0.7394±0.0316	0.67±0.0326	0.8286±0.0202
Affymetrix_6.0	0.7621±0.0587	0.7571±0.05	0.5736±0.0577	0.6828±0.0592	0.6471±0.0566	0.8121±0.0392
OmniZhongHua	0.7969±0.0411	0.7917±0.0376	0.6416±0.0489	0.734±0.0454	0.6987±0.042	0.8561±0.0293
Multi-Ethnic_EUR_EAS_SAS	0.7793±0.0478	0.7971±0.0423	0.6698±0.0531	0.7523±0.0501	0.7181±0.047	0.8652±0.0321
Multi-Ethnic_Global	0.8013±0.0448	0.8053±0.0404	0.6741±0.0515	0.7562±0.048	0.7219±0.0458	0.8677±0.0316
Infinium_GDA	0.8058±0.0426	0.8095±0.0372	0.6767±0.049	0.7605±0.0445	0.7261±0.0432	0.8707±0.0287
Axiom_GW_PanAFR	0.8498±0.036	0.799±0.0337	0.6232±0.0422	0.7256±0.0422	0.6967±0.038	0.8463±0.0252
Infinium_Omni2.5	0.8391±0.0398	0.8202±0.0367	0.6625±0.0464	0.7731±0.0451	0.7313±0.0408	0.8669±0.0273
Infinium_Omni5	0.8537±0.0373	0.8498±0.0333	0.6878±0.0431	0.8347±0.0393	0.7692±0.0383	0.8821±0.0246

Table S. 3 Mean and the standard deviation of imputation accuracy r^2 measured in 22 autosomes at the MAF bin of (0.05-0.5].

Array name	AFR	AMR	EAS	EUR	SAS	VNP
CytoSNP-12	0.7399±0.0449	0.86±0.0352	0.8202±0.0401	0.8536±0.0372	0.8266±0.0406	0.9132±0.0258
Infinium_Core	0.7822±0.0532	0.8748±0.0374	0.8361±0.0425	0.8669±0.039	0.8442±0.0439	0.9206±0.0268
Infinium_OncoArray	0.8154±0.0434	0.8949±0.0313	0.8563±0.0366	0.8909±0.0317	0.8686±0.0365	0.9319±0.0232
PsychArray	0.8085±0.0433	0.8894±0.0321	0.8508±0.0373	0.8838±0.0327	0.861±0.0375	0.929±0.0235
Axiom_GW_ASI	0.8294±0.0714	0.8894±0.055	0.8633±0.0617	0.8814±0.0581	0.8686±0.0626	0.9298±0.0423
Infinium_GSA	0.8028±0.0425	0.8924±0.0317	0.8557±0.0358	0.8878±0.0328	0.868±0.0355	0.9363±0.0204
Axiom_GW_CHB	0.8424±0.0316	0.904±0.0227	0.8803±0.0229	0.8955±0.0226	0.8821±0.0254	0.9465±0.0121
Axiom_JAPONICA	0.8397±0.0321	0.9188±0.0216	0.9115±0.0224	0.912±0.0213	0.9035±0.0237	0.9641±0.0109
Axiom_GW_EUR	0.8077±0.0811	0.8889±0.0603	0.8484±0.07	0.8911±0.062	0.8674±0.0687	0.9195±0.0503
Infinium_Chinese	0.856±0.0316	0.9171±0.0255	0.8931±0.0292	0.9113±0.0254	0.9009±0.0287	0.9531±0.0177
Infinium_JSA	0.8112±0.0363	0.8975±0.0252	0.8718±0.0276	0.8882±0.0256	0.873±0.028	0.9447±0.0142
Axiom_UKB	0.8367±0.0264	0.9241±0.0209	0.8851±0.0234	0.9311±0.0199	0.906±0.0233	0.9503±0.0124
CytoSNP-850K	0.8905±0.03	0.9272±0.0248	0.9002±0.0288	0.925±0.0239	0.9113±0.0287	0.9564±0.0171
Axiom_PMRA	0.8799±0.0242	0.9181±0.0228	0.892±0.0251	0.9147±0.0224	0.8974±0.0249	0.9529±0.0134
Axiom_PMDA	0.8907±0.0167	0.9279±0.0151	0.8921±0.017	0.9263±0.0153	0.9078±0.0174	0.9545±0.0079
Affymetrix_6.0	0.8852±0.0407	0.9165±0.0304	0.8847±0.0372	0.9114±0.0319	0.8975±0.0367	0.9468±0.0221
OmniZhongHua	0.9105±0.0258	0.9389±0.0224	0.9211±0.0248	0.9371±0.0215	0.9276±0.0251	0.9666±0.0141
Multi-Ethnic_EUR_EAS_SAS	0.8952±0.0321	0.9329±0.0262	0.9097±0.0305	0.9316±0.0257	0.9188±0.03	0.9605±0.0182
Multi-Ethnic_Global	0.9075±0.0287	0.9367±0.0249	0.9135±0.0287	0.9353±0.0243	0.9226±0.0283	0.963±0.0172
Infinium_GDA	0.9116±0.0255	0.94±0.0221	0.9174±0.0251	0.9386±0.0216	0.9265±0.0245	0.9651±0.014
Axiom_GW_PanAFR	0.9393±0.0204	0.9395±0.0204	0.918±0.0226	0.9364±0.0197	0.9294±0.0227	0.9661±0.0123
Infinium_Omni2.5	0.9328±0.0236	0.9467±0.0214	0.9291±0.0236	0.9465±0.0205	0.9373±0.0238	0.9705±0.0136
Infinium_Omni5	0.9415±0.0208	0.9555±0.0189	0.9385±0.0205	0.9567±0.0174	0.9475±0.0203	0.9767±0.0107

Table S. 4 Mean and the standard deviation of imputation coverage measured in 22 autosomes at the MAF bin of (0-0.01].

Array name	AFR	AMR	EAS	EUR	SAS	VNP
CytoSNP-12	0.1588±0.0294	0.3343±0.0468	0.0882±0.0193	0.1583±0.0304	0.1271±0.0228	0.2822±0.0387
Infinium_Core	0.1728±0.0406	0.3633±0.0636	0.0972±0.024	0.1758±0.0398	0.1362±0.0299	0.2906±0.0422
Infinium_OncoArray	0.2133±0.0369	0.4207±0.0547	0.1195±0.023	0.2254±0.0368	0.1719±0.0277	0.3279±0.0374
PsychArray	0.2077±0.0354	0.4068±0.0539	0.1132±0.0223	0.2081±0.0343	0.158±0.0266	0.3146±0.037
Axiom_GW_ASI	0.2595±0.0591	0.4572±0.0813	0.1471±0.0355	0.2475±0.0548	0.1929±0.0444	0.3663±0.0617
Infinium_GSA	0.2056±0.0375	0.4203±0.0574	0.1259±0.0245	0.2292±0.0419	0.1904±0.0312	0.3518±0.0402
Axiom_GW_CHB	0.2524±0.034	0.4567±0.0435	0.1472±0.0202	0.2397±0.028	0.1896±0.0221	0.3807±0.026
Axiom_JAPONICA	0.2497±0.0372	0.4567±0.0492	0.1595±0.028	0.2549±0.0374	0.2066±0.0304	0.4172±0.04
Axiom_GW_EUR	0.2507±0.0615	0.4471±0.0887	0.1429±0.0365	0.2532±0.0641	0.2044±0.0492	0.3561±0.0645
Infinium_Chinese	0.258±0.0356	0.4708±0.0472	0.1553±0.0253	0.2534±0.0352	0.1998±0.0281	0.3873±0.037
Infinium_JSA	0.2156±0.0347	0.4177±0.0507	0.1441±0.0249	0.221±0.0341	0.1782±0.0271	0.3864±0.0392
Axiom_UKB	0.2412±0.0276	0.4743±0.0406	0.1438±0.0203	0.2905±0.0347	0.2305±0.026	0.3767±0.0293
CytoSNP-850K	0.3158±0.0402	0.525±0.0453	0.1648±0.0255	0.2884±0.0333	0.222±0.0272	0.3949±0.0356
Axiom_PMRA	0.2735±0.0328	0.5169±0.0422	0.1569±0.023	0.2646±0.0327	0.2066±0.0265	0.3961±0.035
Axiom_PMDA	0.2814±0.0245	0.531±0.0297	0.1461±0.0169	0.2781±0.025	0.2138±0.0213	0.3782±0.027
Affymetrix_6.0	0.317±0.0534	0.5161±0.06	0.1584±0.0296	0.2731±0.043	0.2102±0.0347	0.3746±0.0449
OmniZhongHua	0.3547±0.0383	0.5593±0.0434	0.1928±0.0266	0.3178±0.0335	0.2505±0.0282	0.4373±0.0349
Multi-Ethnic_EUR_EAS_SAS	0.3348±0.0432	0.553±0.0512	0.1953±0.0289	0.3283±0.0396	0.2613±0.032	0.4425±0.0392
Multi-Ethnic_Global	0.3519±0.0434	0.5711±0.0495	0.1967±0.0283	0.3302±0.0382	0.2624±0.031	0.4432±0.0389
Infinium_GDA	0.357±0.0418	0.5759±0.0471	0.1989±0.0271	0.3346±0.0363	0.2657±0.0296	0.448±0.0374
Axiom_GW_PanAFR	0.4182±0.0383	0.6013±0.0384	0.1913±0.0233	0.3228±0.0301	0.2495±0.025	0.4241±0.0297
Infinium_Omni2.5	0.4184±0.0417	0.6126±0.0442	0.2126±0.0266	0.3601±0.0365	0.2865±0.0302	0.4561±0.0347
Infinium_Omni5	0.4517±0.0396	0.6655±0.043	0.2322±0.0265	0.4667±0.04	0.3455±0.0316	0.4807±0.0335

Table S. 5 Mean and the standard deviation of imputation coverage measured in 22 autosomes at the MAF bin of (0.01-0.05].

Array name	AFR	AMR	EAS	EUR	SAS	VNP
CytoSNP-12	0.2618±0.0502	0.3854±0.0655	0.2533±0.0428	0.3263±0.0573	0.2763±0.0483	0.504±0.061
Infinium_Core	0.3099±0.0837	0.4403±0.082	0.2786±0.0543	0.3584±0.0718	0.3002±0.0627	0.5207±0.0655
Infinium_OncoArray	0.3922±0.0736	0.5241±0.0684	0.3281±0.0508	0.4503±0.0642	0.3739±0.0557	0.578±0.0568
PsychArray	0.3766±0.0721	0.4939±0.0706	0.3093±0.0509	0.4107±0.0638	0.3414±0.0562	0.5564±0.0578
Axiom_GW_ASI	0.5082±0.1141	0.5651±0.1018	0.3972±0.072	0.4749±0.0893	0.4261±0.0802	0.6405±0.0909
Infinium_GSA	0.3524±0.074	0.5587±0.0723	0.4012±0.0528	0.5747±0.0622	0.4051±0.0582	0.661±0.0567
Axiom_GW_CHB	0.4872±0.0603	0.5608±0.0496	0.4483±0.0393	0.4611±0.0467	0.412±0.0421	0.6925±0.0327
Axiom_JAPONICA	0.4701±0.0674	0.574±0.0624	0.4755±0.0555	0.4928±0.0601	0.4351±0.054	0.7465±0.0527
Axiom_GW_EUR	0.4638±0.1157	0.5748±0.1126	0.3735±0.0726	0.5374±0.1032	0.4456±0.0898	0.6148±0.0946
Infinium_Chinese	0.5023±0.0641	0.5963±0.0558	0.4523±0.0492	0.5209±0.0547	0.4487±0.0501	0.7026±0.0489
Infinium_JSA	0.3904±0.066	0.5205±0.0608	0.4602±0.0483	0.4392±0.0546	0.3855±0.0508	0.7207±0.0494
Axiom_UKB	0.4199±0.0479	0.644±0.0488	0.3804±0.0411	0.6663±0.0543	0.4916±0.043	0.655±0.0418
CytoSNP-850K	0.6227±0.0669	0.6398±0.0527	0.4196±0.0462	0.5378±0.0546	0.4809±0.0504	0.6741±0.0475
Axiom_PMRA	0.6102±0.0483	0.6421±0.0481	0.4726±0.0472	0.5544±0.0546	0.4569±0.0443	0.7161±0.045
Axiom_PMDA	0.655±0.037	0.6849±0.0315	0.4249±0.0386	0.5957±0.0393	0.4894±0.0387	0.6708±0.0419
Affymetrix_6.0	0.6204±0.0924	0.6206±0.0713	0.4047±0.0559	0.505±0.0694	0.4616±0.064	0.6428±0.0628
OmniZhongHua	0.6954±0.0582	0.6846±0.0473	0.4782±0.0492	0.5838±0.0524	0.531±0.047	0.7405±0.046
Multi-Ethnic_EUR_EAS_SAS	0.6522±0.0734	0.699±0.0561	0.5272±0.0563	0.6234±0.0613	0.5726±0.0566	0.7684±0.0512
Multi-Ethnic_Global	0.6965±0.0689	0.7099±0.0539	0.5309±0.0546	0.627±0.0595	0.5765±0.0554	0.7709±0.0498
Infinium_GDA	0.7053±0.0642	0.716±0.0495	0.5337±0.0523	0.6327±0.0558	0.5818±0.0524	0.7756±0.0468
Axiom_GW_PanAFR	0.8012±0.0452	0.6917±0.039	0.4611±0.0396	0.5687±0.0455	0.5302±0.0406	0.708±0.037
Infinium_Omni2.5	0.7807±0.0521	0.7349±0.045	0.505±0.0463	0.6559±0.0537	0.5846±0.0467	0.76±0.0422
Infinium_Omni5	0.8045±0.047	0.7885±0.0398	0.537±0.0444	0.7738±0.0471	0.6508±0.0457	0.7895±0.0392

Table S. 6 Mean and the standard deviation of imputation coverage measured in 22 autosomes at the MAF bin of (0.05-0.5].

Array name	AFR	AMR	EAS	EUR	SAS	VNP
CytoSNP-12	0.5126±0.0643	0.7797±0.0591	0.7276±0.059	0.7721±0.06	0.7239±0.0628	0.8687±0.047
Infinium_Core	0.6075±0.0912	0.807±0.0585	0.7572±0.0604	0.7954±0.0589	0.7586±0.0654	0.8825±0.0428
Infinium_OncoArray	0.6767±0.077	0.8454±0.0463	0.7892±0.0508	0.8381±0.0464	0.8019±0.0535	0.9041±0.0367
PsychArray	0.6593±0.0758	0.8333±0.0483	0.7789±0.0522	0.8226±0.0485	0.7855±0.055	0.8978±0.0367
Axiom_GW_ASI	0.7409±0.1153	0.8411±0.0828	0.8089±0.0854	0.8292±0.0839	0.8127±0.09	0.9013±0.0687
Infinium_GSA	0.64±0.0851	0.8509±0.0493	0.785±0.0548	0.8382±0.0522	0.8035±0.057	0.9211±0.0351
Axiom_GW_CHB	0.7329±0.0545	0.8584±0.0291	0.8198±0.0275	0.8413±0.0288	0.8202±0.0323	0.93±0.0174
Axiom_JAPONICA	0.73±0.0622	0.8936±0.0313	0.8851±0.031	0.8778±0.031	0.8673±0.0352	0.964±0.0175
Axiom_GW_EUR	0.6989±0.1257	0.8428±0.0898	0.7867±0.0942	0.8483±0.0888	0.8133±0.0972	0.8865±0.0794
Infinium_Chinese	0.7676±0.0577	0.8869±0.0339	0.8496±0.0387	0.874±0.0342	0.8608±0.0393	0.9433±0.0253
Infinium_JSA	0.641±0.0679	0.848±0.0406	0.8026±0.0439	0.8269±0.0415	0.8001±0.045	0.9352±0.0251
Axiom_UKB	0.7158±0.047	0.9109±0.0255	0.8418±0.0303	0.9202±0.024	0.8815±0.0299	0.9473±0.019
CytoSNP-850K	0.8413±0.0489	0.8984±0.0316	0.8575±0.0378	0.8932±0.0308	0.8738±0.038	0.9436±0.0254
Axiom_PMRA	0.8303±0.0372	0.8952±0.0299	0.8564±0.0337	0.888±0.0296	0.8618±0.0335	0.9506±0.0207
Axiom_PMDA	0.8621±0.024	0.9113±0.0181	0.8489±0.0235	0.9073±0.0185	0.8762±0.0239	0.9513±0.0119
Affymetrix_6.0	0.8264±0.0734	0.8761±0.0477	0.8291±0.0543	0.867±0.0492	0.8466±0.0562	0.9242±0.0388
OmniZhongHua	0.8845±0.0365	0.9183±0.0269	0.8923±0.0304	0.9142±0.0257	0.9027±0.0306	0.9615±0.0204
Multi-Ethnic_EUR_EAS_SAS	0.8599±0.051	0.9147±0.0317	0.8774±0.0387	0.9109±0.0319	0.8925±0.0379	0.956±0.0251
Multi-Ethnic_Global	0.8797±0.0453	0.9188±0.0301	0.8816±0.0371	0.915±0.0305	0.8966±0.0362	0.9589±0.0237
Infinium_GDA	0.8866±0.0398	0.923±0.0271	0.8868±0.0326	0.9195±0.027	0.9018±0.0321	0.9623±0.0204
Axiom_GW_PanAFR	0.9233±0.0242	0.9148±0.0239	0.8833±0.0262	0.9089±0.0231	0.901±0.0265	0.9565±0.0175
Infinium_Omni2.5	0.9143±0.0294	0.928±0.0249	0.9011±0.0279	0.9279±0.0239	0.9148±0.0279	0.965±0.0191
Infinium_Omni5	0.9245±0.0253	0.9404±0.0219	0.9128±0.0246	0.9426±0.0204	0.928±0.0242	0.9725±0.0156

Table S. 7 Mean and the standard deviation of PGS correlation of 23 human genotyping array of the phenotype height.

Arrays	AFR	AMR	EAS	EUR	SAS	VNP
CytoSNP-12	0.8731±0.0029	0.9437±0.0036	0.919±0.007	0.9554±0.0048	0.9332±0.0042	0.9566±0.0067
Infinium_Core	0.8993±0.0056	0.9589±0.0014	0.9207±0.0081	0.9616±0.0014	0.9352±0.0049	0.9575±0.0065
Infinium_OncoArray	0.9276±0.0074	0.9738±0.0027	0.9463±0.0108	0.9806±7e-04	0.9587±0.006	0.971±0.007
PsychArray	0.9223±0.0084	0.9699±0.0019	0.9429±0.0107	0.9752±9e-04	0.9501±0.0065	0.9638±0.0062
Axiom_GW_ASI	0.9237±0.0053	0.961±0.0073	0.942±9e-04	0.9662±0.0066	0.9501±0.001	0.9627±8e-04
Infinium_GSA	0.9221±0.002	0.9722±0.001	0.9477±0.0037	0.979±0.0017	0.959±0.0016	0.9706±0.0046
Axiom_GW_CHB	0.934±0.0038	0.9715±0.0016	0.9566±0.0062	0.9733±0.0018	0.957±0.005	0.9722±0.0046
Axiom_JAPONICA	0.9349±0.0011	0.974±0.0013	0.976±0.001	0.98±0.0025	0.9676±0.0013	0.9847±0.002
Axiom_GW_EUR	0.908±0.0075	0.965±0.0027	0.9342±0.0022	0.9733±0.0039	0.9512±0.003	0.9586±0.0014
Infinium_Chinese	0.9464±0.002	0.9784±0.0011	0.9628±0.0026	0.9825±5e-04	0.9656±0.0023	0.9795±0.0037
Infinium_JSA	0.9189±0.0047	0.9625±0.0032	0.9486±0.0064	0.9772±5e-04	0.9575±0.0028	0.972±0.0046
Axiom_UKB	0.9361±0.0011	0.9837±7e-04	0.9651±0.0039	0.9915±6e-04	0.9744±0.0015	0.9784±0.0035
CytoSNP-850K	0.9679±0.0026	0.9876±0.0011	0.9789±0.0019	0.9908±5e-04	0.9844±0.0016	0.988±0.0025
Axiom_PMRA	0.9413±0.0025	0.9766±0.0015	0.9608±0.0038	0.9844±0.0017	0.9633±0.0026	0.9774±0.0033
Axiom_PMDA	0.9457±0.001	0.9782±0.0014	0.9566±0.0042	0.9853±8e-04	0.965±0.0017	0.9774±0.0035
Affymetrix_6.0	0.9599±0.003	0.9804±0.001	0.9635±0.0051	0.9835±0.0021	0.9696±0.0027	0.9783±0.0029
OmniZhongHua	0.9766±0.0011	0.9906±6e-04	0.9879±0.0015	0.9943±3e-04	0.9872±7e-04	0.9914±0.0014
Multi-Ethnic_EUR_EAS_SAS	0.9644±6e-04	0.9877±6e-04	0.9742±0.0039	0.9914±6e-04	0.9801±0.0014	0.9854±0.0021
Multi-Ethnic_Global	0.9681±6e-04	0.9872±7e-04	0.975±0.0032	0.9918±3e-04	0.9804±0.0022	0.9855±0.0021
Infinium_GDA	0.97±0.0012	0.9878±5e-04	0.9765±0.0037	0.9925±3e-04	0.983±0.0013	0.9865±0.0022
Axiom_GW_PanAFR	0.9825±4e-04	0.9845±9e-04	0.9804±0.0021	0.9882±0.0017	0.9797±8e-04	0.9885±0.0014
Infinium_Omni2.5	0.9863±2e-04	0.9939±3e-04	0.9907±0.0013	0.9965±1e-04	0.9917±4e-04	0.9931±9e-04
Infinium_Omni5	0.9903±8e-04	0.9969±2e-04	0.9938±7e-04	0.9983±1e-04	0.9954±3e-04	0.9952±6e-04

Table S. 8 Mean and the standard deviation of PGS correlation of 23 human genotyping array of the phenotype body mass index (BMI).

Arrays	AFR	AMR	EAS	EUR	SAS	VNP
CytoSNP-12	0.8847±0.0204	0.9482±0.0134	0.9048±0.0325	0.9422±0.0177	0.9312±0.0157	0.9565±0.0132
Infinium_Core	0.9098±0.0164	0.9533±0.0139	0.9201±0.029	0.9482±0.0168	0.9403±0.021	0.9593±0.0134
Infinium_OncoArray	0.9286±0.0167	0.9701±0.0101	0.9412±0.0226	0.9655±0.0137	0.9572±0.0176	0.9691±0.0115
PsychArray	0.9256±0.0205	0.9645±0.0145	0.9317±0.0282	0.962±0.0139	0.9532±0.0169	0.967±0.0115
Axiom_GW_ASI	0.9428±0.004	0.9714±0.0049	0.9512±0.009	0.9618±0.0022	0.9569±0.0089	0.9711±0.004
Infinium_GSA	0.9243±0.0095	0.9713±0.007	0.9415±0.0156	0.9661±0.0057	0.958±0.0108	0.9726±0.0071
Axiom_GW_CHB	0.9378±0.0083	0.9714±0.007	0.9477±0.0147	0.9626±0.0103	0.9584±0.0113	0.9728±0.0081
Axiom_JAPONICA	0.9495±0.0045	0.9772±0.0068	0.9721±0.0067	0.9742±0.0059	0.9694±0.009	0.9851±0.0042
Axiom_GW_EUR	0.9375±0.0045	0.9717±0.0036	0.9435±0.0086	0.9692±0.0027	0.959±0.0058	0.9658±0.0063
Infinium_Chinese	0.949±0.0063	0.9781±0.0068	0.9621±0.0138	0.9733±0.0094	0.9687±0.0124	0.9797±0.0064
Infinium_JSA	0.9256±0.0144	0.9635±0.0105	0.9426±0.0184	0.9618±0.0109	0.9547±0.0159	0.9717±0.0093
Axiom_UKB	0.939±0.0066	0.9824±0.0034	0.957±0.0091	0.986±0.0031	0.9742±0.0052	0.9785±0.0048
CytoSNP-850K	0.9708±0.0056	0.9882±0.0043	0.9763±0.0083	0.9863±0.0053	0.9817±0.0065	0.9866±0.0039
Axiom_PMRA	0.946±0.0109	0.9776±0.0057	0.9494±0.0137	0.9745±0.005	0.9652±0.01	0.976±0.0055
Axiom_PMDA	0.9489±0.0054	0.9806±0.0031	0.9523±0.01	0.9784±0.0053	0.9681±0.0071	0.9761±0.0053
Affymetrix_6.0	0.9657±0.0052	0.981±0.0045	0.9612±0.0102	0.9782±0.0053	0.9739±0.0078	0.978±0.0057
OmniZhongHua	0.9783±0.004	0.9905±0.0028	0.985±0.0052	0.9903±0.0033	0.988±0.0043	0.9919±0.0022
Multi-Ethnic_EUR_EAS_SAS	0.9667±0.0047	0.987±0.0035	0.9746±0.0067	0.9863±0.0036	0.9812±0.0051	0.9854±0.0037
Multi-Ethnic_Global	0.9685±0.0044	0.9876±0.0037	0.9753±0.0066	0.9857±0.004	0.9812±0.0061	0.9856±0.0038
Infinium_GDA	0.9692±0.0047	0.9891±0.0033	0.976±0.0083	0.9869±0.0038	0.9826±0.0058	0.9863±0.0041
Axiom_GW_PanAFR	0.9864±0.0012	0.9866±0.0032	0.9795±0.0047	0.9846±0.0032	0.9826±0.0041	0.9873±0.0041
Infinium_Omni2.5	0.9875±0.001	0.9941±0.0017	0.9891±0.0035	0.9944±0.0016	0.9926±0.0024	0.9937±0.0013
Infinium_Omni5	0.9906±6e-04	0.9966±7e-04	0.9929±0.0017	0.9973±6e-04	0.9951±8e-04	0.9951±9e-04

Table S. 9 Mean and the standard deviation of PGS correlation of 23 human genotyping array of the phenotype type 2 diabetes (T2D).

Arrays	AFR	AMR	EAS	EUR	SAS	VNP
CytoSNP-12	0.8948±0.0178	0.9633±0.0052	0.9119±0.0265	0.9515±0.0058	0.9309±0.0082	0.9592±0.0089
Infinium_Core	0.9054±0.0205	0.9676±0.0059	0.9249±0.0277	0.9616±0.0076	0.94±0.0105	0.9651±0.0084
Infinium_OncoArray	0.9433±0.0053	0.9801±0.0067	0.9496±0.0214	0.9781±0.0097	0.9633±0.014	0.975±0.0102
PsychArray	0.9322±0.0044	0.9766±0.0074	0.9406±0.0275	0.972±0.009	0.9558±0.0145	0.9713±0.0101
Axiom_GW_ASI	0.9513±0.0069	0.9798±0.0019	0.961±0.0055	0.9777±0.003	0.9703±0.0024	0.9782±0.0025
Infinium_GSA	0.9391±0.0051	0.978±0.0043	0.9469±0.0229	0.9745±0.0073	0.965±0.0111	0.975±0.0085
Axiom_GW_CHB	0.9403±0.0127	0.9741±0.0048	0.948±0.0136	0.968±0.0031	0.9591±0.0057	0.9745±0.0066
Axiom_JAPONICA	0.9463±0.0091	0.9842±0.0025	0.9758±0.0076	0.9812±0.0051	0.9729±0.0069	0.9873±0.0041
Axiom_GW_EUR	0.9407±0.0143	0.9834±0.0014	0.9572±0.0143	0.9851±0.0025	0.9738±0.0051	0.9774±0.004
Infinium_Chinese	0.9587±0.0028	0.9858±0.0052	0.968±0.0158	0.984±0.0086	0.9754±0.0114	0.985±0.0072
Infinium_JSA	0.9377±0.0075	0.9787±0.0068	0.9507±0.0195	0.974±0.0117	0.9613±0.0171	0.9752±0.0085
Axiom_UKB	0.9396±0.0052	0.9843±0.0024	0.9595±0.019	0.9886±0.004	0.9778±0.009	0.9815±0.0073
CytoSNP-850K	0.9713±0.0022	0.9911±0.0019	0.9773±0.0084	0.9902±0.0021	0.9847±0.0068	0.9883±0.004
Axiom_PMRA	0.9545±0.0041	0.9816±0.0039	0.9624±0.0135	0.9818±0.0048	0.9699±0.0086	0.9814±0.0062
Axiom_PMDA	0.9561±0.0039	0.9828±0.0031	0.9602±0.0147	0.9826±0.005	0.9727±0.0064	0.9794±0.0067
Affymetrix_6.0	0.9708±0.0046	0.9886±0.0023	0.968±0.0081	0.9873±0.0029	0.9794±0.0067	0.9839±0.0028
OmniZhongHua	0.978±0.0036	0.9934±0.0012	0.9829±0.0036	0.9934±0.0011	0.9898±0.0034	0.9915±0.0024
Multi-Ethnic_EUR_EAS_SAS	0.9726±0.0027	0.9913±0.0031	0.9765±0.0096	0.991±0.0038	0.9844±0.0055	0.9881±0.0038
Multi-Ethnic_Global	0.9747±0.002	0.9914±0.003	0.9743±0.0094	0.9913±0.004	0.9844±0.0045	0.9878±0.0038
Infinium_GDA	0.9768±0.0023	0.9914±0.0023	0.9783±0.0116	0.9904±0.0028	0.9855±0.006	0.989±0.0045
Axiom_GW_PanAFR	0.9867±0.0015	0.991±6e-04	0.9793±0.0054	0.9922±0.0025	0.9859±0.002	0.9898±0.0026
Infinium_Omni2.5	0.9869±0.0013	0.9953±7e-04	0.9888±0.0038	0.9961±0.001	0.9933±0.0029	0.993±0.0013
Infinium_Omni5	0.9903±8e-04	0.997±0.0011	0.9919±0.0025	0.9977±0.001	0.9955±0.0022	0.995±0.0018

Table S. 10 Mean absolute difference of percentile ranking between PGSs estimated from imputed genotyping data of 23 SNP arrays and PGS estimated from WGS in 6 different populations with PRsice p-value setting of 1e-8.

Array_name	Trait	AFR	AMR	EAS	EUR	SAS	VNP
CytoSNP-12	Height	11.75223896	7.614879286	8.687641723	6.910426111	8.149012425	6.121956885
Infinium_Core	Height	10.122196	6.487056615	8.562846435	6.439691869	7.904784607	5.98987859
Infinium_OncoArray	Height	8.185690319	4.752966971	6.669658604	4.389962412	5.835539329	4.605123299
PsychArray	Height	8.444089435	5.271200658	6.825554296	4.974921841	6.32775875	5.361276455
Axiom_GW_ASI	Height	9.266663768	7.090001578	7.899108718	6.787505583	7.333107506	6.032592435
Infinium_GSA	Height	8.889478876	5.42235215	7.315680902	5.071361098	6.820396368	4.864752614
Axiom_GW_CHB	Height	8.254581492	5.430657177	6.368102797	5.417593841	6.132041937	4.713088939
Axiom_JAPONICA	Height	8.133278098	5.201438431	4.913470018	5.084404112	5.948452875	3.620933327
Axiom_GW_EUR	Height	10.25883398	6.195550167	8.301445578	5.768569498	7.499550437	6.268896447
Infinium_Chinese	Height	7.376619572	4.640018603	5.637833837	4.401424455	5.934652331	3.964021951
Infinium_JSA	Height	8.877119662	5.723824631	6.917674477	4.979664755	6.484583119	4.818692523
Axiom_UKB	Height	8.055231953	4.320275062	5.609489166	3.058389227	5.134638948	4.144522392
CytoSNP-850K	Height	5.714076458	3.491433365	4.375314941	3.250082013	3.763784862	3.026679422
Axiom_PMRA	Height	7.620828479	4.731373901	6.096466364	4.29273267	5.979399551	4.23605206
Axiom_PMDA	Height	7.690406275	4.767085517	6.088199169	4.066653755	5.799574274	4.267054044
Affymetrix_6.0	Height	6.512161237	4.232241776	5.703577727	4.374547941	5.255498262	4.240579334
OmniZhongHua	Height	4.916220552	3.126842678	3.297036407	2.536668656	3.625361219	2.585368638
Multi-Ethnic_EUR_EAS_SAS	Height	6.210276	3.439942197	4.896935626	3.16115237	4.294478528	3.473009574
Multi-Ethnic_Global	Height	5.777016898	3.569500619	5.144951499	3.013726784	4.159400471	3.437381897
Infinium_GDA	Height	5.533723488	3.488941857	4.689074704	2.855234399	4.003412498	3.248319004
Axiom_GW_PanAFR	Height	4.340601619	4.001362024	4.220993953	3.839784355	4.374772605	3.210230852
Infinium_Omni2.5	Height	3.859965532	2.477389564	2.888400731	1.975819042	2.839148381	2.369929453
Infinium_Omni5	Height	3.160983336	1.881088623	2.415595868	1.457655657	2.067154286	2.029596561
CytoSNP-12	BMI	9.586401203	6.375769253	8.234126984	6.162231383	7.622082544	5.489713246

Infinium_Core	BMI	8.647558712	5.886603161	7.263321995	5.424708212	6.086876519	5.100859788
Infinium_OncoArray	BMI	7.275685994	4.219784235	6.078357269	4.231074784	5.001233685	4.251799099
PsychArray	BMI	7.163995322	4.610951009	6.18425611	4.780462355	5.566219613	4.619886149
Axiom_GW_ASI	BMI	7.292622694	4.841000257	6.67398904	5.972119569	6.078094354	5.081668084
Infinium_GSA	BMI	7.855195791	4.612612014	6.669658604	5.179657641	5.576256372	4.447849742
Axiom_GW_CHB	BMI	7.574366991	4.679882733	6.201184177	4.893106569	5.441178316	4.281915312
Axiom_JAPONICA	BMI	6.986388844	4.068632743	4.76505417	4.099854155	4.437502352	3.194975907
Axiom_GW_EUR	BMI	7.872819114	5.064405485	7.225135425	5.382021983	6.156297439	5.418851568
Infinium_Chinese	BMI	6.673288764	4.016311073	5.195735702	3.929899727	4.136399563	3.641699735
Infinium_JSA	BMI	7.691550646	5.188150387	6.81531872	4.915240169	5.300663681	4.262822027
Axiom_UKB	BMI	7.947432144	3.636771338	5.83388448	3.171428684	4.407810272	3.990299824
CytoSNP-850K	BMI	4.987171594	2.546321288	4.156037415	2.62717927	3.540885159	3.152262062
Axiom_PMRA	BMI	6.288322145	3.857685057	5.932697153	4.429881941	4.975305389	4.293725592
Axiom_PMDA	BMI	6.775595588	4.176598095	6.055524061	4.053610741	4.964014035	4.337226789
Affymetrix_6.0	BMI	5.30484916	3.836922489	5.54925674	4.037010541	4.162746057	4.061259921
OmniZhongHua	BMI	4.233717308	2.557117823	3.21042769	2.364738013	2.877622626	2.291784769
Multi-Ethnic_EUR_EAS_SAS	BMI	5.457050588	3.171689824	4.365866717	2.882110913	3.657144291	3.237985009
Multi-Ethnic_Global	BMI	5.116256715	3.047944921	4.440665155	2.896734899	3.583541387	3.218990142
Infinium_GDA	BMI	4.934988247	2.907589964	3.951719577	2.712551727	3.36398727	3.086321334
Axiom_GW_PanAFR	BMI	3.501548335	3.219858981	4.097379693	3.261148813	3.797658926	2.953160431
Infinium_Omni2.5	BMI	3.597446678	1.862817563	2.8462774	1.848946085	2.387075999	2.302217183
Infinium_Omni5	BMI	3.09827177	1.688411996	2.418745276	1.270310542	2.103955738	2.098686697
CytoSNP-12	Type_2_diabetes	11.34736028	6.189736648	8.603395062	6.821101226	8.292872646	5.872661565
Infinium_Core	Type_2_diabetes	11.1795954	6.012839572	6.812169312	5.769359983	6.871834761	5.559197058
Infinium_OncoArray	Type_2_diabetes	7.040860934	3.7787873	5.393361048	3.030326984	4.115489648	3.866291887
PsychArray	Type_2_diabetes	7.860688774	4.087734306	4.941421013	3.90262797	4.981578364	4.087636212
Axiom_GW_ASI	Type_2_diabetes	7.564525395	4.937338571	6.275982615	3.978119355	5.278917368	4.700196051

Infinium_GSA	Type_2_diabetes	7.13057967	4.375088241	4.632779038	3.692754013	4.398609909	3.738347191
Axiom_GW_CHB	Type_2_diabetes	8.268313951	5.174862344	6.402746284	5.574900498	6.201462858	4.638684177
Axiom_JAPONICA	Type_2_diabetes	7.670265334	4.006345041	4.001322751	3.26075357	4.302424296	2.690972222
Axiom_GW_EUR	Type_2_diabetes	8.457135272	4.076107268	4.880794911	3.286444356	4.19452913	4.27138448
Infinium_Chinese	Type_2_diabetes	6.100645197	3.227333505	3.522219073	1.477417799	3.093412958	2.252318752
Infinium_JSA	Type_2_diabetes	6.731422843	3.630127316	5.030391786	3.097913513	3.901372109	3.851233781
Axiom_UKB	Type_2_diabetes	7.895935421	3.775465289	4.529635928	2.273436913	3.183325597	3.006897203
CytoSNP-850K	Type_2_diabetes	5.200024718	2.576219386	3.768660242	2.606626642	2.470715663	2.381247638
Axiom_PMRA	Type_2_diabetes	6.980666986	3.937413316	4.62569287	3.231900841	4.028086199	3.466218663
Axiom_PMDA	Type_2_diabetes	6.599362356	3.957345381	4.987087428	3.027165042	4.012194663	3.539639235
Affymetrix_6.0	Type_2_diabetes	6.040222374	3.381807008	5.278013983	3.271029884	3.545067142	3.924949609
OmniZhongHua	Type_2_diabetes	4.988315966	2.773879029	3.681264172	1.975819042	2.30970931	2.282631803
Multi-Ethnic_EUR_EAS_SAS	Type_2_diabetes	4.663314421	2.079578769	3.822593852	1.778988099	2.956662108	2.648750472
Multi-Ethnic_Global	Type_2_diabetes	4.563982963	2.39018678	4.129661124	1.646976985	3.211763082	2.635070232
Infinium_GDA	Type_2_diabetes	4.319774055	2.498982634	2.708490804	2.182135813	2.747981148	2.100851915
Axiom_GW_PanAFR	Type_2_diabetes	3.526953385	3.252248586	3.885582011	1.618914742	2.949552737	2.561059146
Infinium_Omni2.5	Type_2_diabetes	3.720581066	2.275577407	2.77265999	1.659229514	1.753505547	2.599737812
Infinium_Omni5	Type_2_diabetes	3.27702262	1.169347806	2.400242504	0.6173693426	1.214447915	1.736111111

Table S. 11 Mean absolute difference of percentile ranking between PGSs estimated from imputed genotyping data of 23 SNP arrays and PGS estimated from WGS in 6 different populations with PRsice p-value setting of 1e-7.

Array_name	Trait	AFR	AMR	EAS	EUR	SAS	VNP
CytoSNP-12	Height	11.81884139	7.518540973	8.584104938	6.947183697	8.221360734	6.124122103
Infinium_Core	Height	10.1997844	6.526090242	8.342781557	6.429020312	7.926949118	5.943129567
Infinium_OncoArray	Height	8.247257513	4.779543057	6.645644369	4.36466687	5.983163336	4.630515401
PsychArray	Height	8.513209482	5.243794069	6.717687075	4.945673869	6.408471025	5.327715577
Axiom_GW_ASI	Height	9.274674369	7.084188059	7.877062862	6.849558711	7.14073628	6.019404289
Infinium_GSA	Height	8.92037691	5.481317842	7.264109347	5.113652084	6.731738325	4.882566453
Axiom_GW_CHB	Height	8.161887389	5.318539312	6.351174729	5.534585726	6.210245022	4.679232804
Axiom_JAPONICA	Height	8.206060134	5.387471036	4.927642353	5.052784684	6.013691813	3.638255071
Axiom_GW_EUR	Height	10.24807688	6.009517561	8.278612371	5.768174255	7.502059627	6.171855316
Infinium_Chinese	Height	7.346637035	4.567764868	5.692161124	4.49746847	5.990272707	3.960774124
Infinium_JSA	Height	8.932964998	5.779468312	6.804689468	5.003774569	6.469946178	4.766333617
Axiom_UKB	Height	8.163260635	4.336885117	5.478788738	3.024398342	5.018379816	4.077203798
CytoSNP-850K	Height	5.638319055	3.464026775	4.293824011	3.401460027	3.767548647	3.017231198
Axiom_PMRA	Height	7.78058276	4.762933003	6.146463215	4.280480141	5.864813212	4.205935847
Axiom_PMDA	Height	7.656075126	4.747153452	6.025604686	4.037801027	5.82550257	4.292347726
Affymetrix_6.0	Height	6.481949826	4.243038311	5.720505795	4.457944184	5.277662773	4.260164714
OmniZhongHua	Height	4.896079612	3.112724132	3.265936004	2.559987985	3.63247059	2.634676556
Multi-Ethnic_EUR_EAS_SAS	Height	6.231332438	3.409213597	4.865835223	3.157990427	4.296151321	3.510507212
Multi-Ethnic_Global	Height	5.77129504	3.497246884	5.054799698	3.007798142	4.19201994	3.487969262
Infinium_GDA	Height	5.643583165	3.487280851	4.598922902	2.819267299	3.985848169	3.31642495
Axiom_GW_PanAFR	Height	4.267819583	4.01215856	4.299729151	3.860732227	4.422029015	3.167615426
Infinium_Omni2.5	Height	3.781461637	2.465762526	2.912808642	2.010995656	2.78729179	2.355461861
Infinium_Omni5	Height	3.187303883	1.940054315	2.402998236	1.441450699	2.125283852	2.028612371
CytoSNP-12	BMI	9.591665312	6.482904102	8.411674855	6.123497583	7.750469428	5.626220396

Infinium_Core	BMI	8.64641434	6.100872858	7.637707861	5.613239055	6.208154031	5.18412226
Infinium_OncoArray	BMI	7.268362015	4.583544419	6.143313807	4.351623855	5.129620569	4.39696712
PsychArray	BMI	7.236319609	4.833525733	6.301177879	4.876901612	5.634804137	4.648526077
Axiom_GW_ASI	BMI	7.3754752	5.124201679	6.67989418	5.995438897	6.075166966	5.178020282
Infinium_GSA	BMI	7.861146523	4.684865749	6.816893424	5.399412669	5.770300392	4.399033919
Axiom_GW_CHB	BMI	7.569102881	4.801136128	6.225198413	4.854768012	5.549073482	4.348249717
Axiom_JAPONICA	BMI	6.982269106	4.170784576	4.749700806	4.440158255	4.564634641	3.285226127
Axiom_GW_EUR	BMI	7.879456469	5.407403101	7.311350466	5.470556383	6.137478515	5.478690319
Infinium_Chinese	BMI	6.642161855	4.228089262	5.172902494	3.98839567	4.252240497	3.681559429
Infinium_JSA	BMI	7.75677983	5.378335506	6.912950365	4.980059998	5.357120454	4.241465105
Axiom_UKB	BMI	7.799808203	3.898379689	6.05079995	3.187238399	4.432483973	4.042560311
CytoSNP-850K	BMI	5.079407948	2.684184737	4.179657974	2.788043113	3.560958678	3.11220553
Axiom_PMRA	BMI	6.435030589	4.253834846	6.147644243	4.456363212	5.03970793	4.394900321
Axiom_PMDA	BMI	6.685647978	4.217292727	6.125992063	4.065468027	5.08194596	4.337620465
Affymetrix_6.0	BMI	5.419515198	3.991395992	5.622480474	4.098668427	4.142672538	4.030454775
OmniZhongHua	BMI	4.39988007	2.724879369	3.246252205	2.350904513	2.86424028	2.272002551
Multi-Ethnic_EUR_EAS_SAS	BMI	5.553177806	3.286299197	4.367835097	3.026769799	3.982502582	3.274990552
Multi-Ethnic_Global	BMI	5.309197773	3.175011835	4.460348954	3.056413013	3.624524822	3.264066043
Infinium_GDA	BMI	5.112365851	2.887657899	4.051713278	2.843377113	3.510356681	3.118898022
Axiom_GW_PanAFR	BMI	3.563115529	3.337790365	4.264298312	3.318063784	3.811877669	2.915859631
Infinium_Omni2.5	BMI	3.609805892	2.116120888	2.906903502	1.991233513	2.504589727	2.242476852
Infinium_Omni5	BMI	3.120014831	1.876936109	2.414808516	1.344220957	2.052517345	2.122799351
CytoSNP-12	Type_2_diabetes	11.34644478	6.207177204	8.29829617	6.852720654	8.178286307	5.795205814
Infinium_Core	Type_2_diabetes	11.12947192	5.741265188	6.611788234	5.757107455	6.95171064	5.39611678
Infinium_OncoArray	Type_2_diabetes	7.346865909	3.503060402	5.036296926	2.929540056	4.271059422	3.737559839
PsychArray	Type_2_diabetes	8.164862756	4.161649046	4.90244709	4.099458913	5.096582902	4.081435815
Axiom_GW_ASI	Type_2_diabetes	7.423309935	4.556968333	5.888999118	4.000648198	5.559946638	4.542528817

Infinium_GSA	Type_2_diabetes	7.286443087	4.326088581	4.590262031	3.839784355	4.3760272	3.670930178
Axiom_GW_CHB	Type_2_diabetes	8.619636044	5.110083133	6.33739607	5.536957183	6.276320357	4.493909832
Axiom_JAPONICA	Type_2_diabetes	7.842378828	3.827786959	3.84345868	3.394740899	4.152291099	2.588321208
Axiom_GW_EUR	Type_2_diabetes	8.744143678	3.975616441	4.951262913	3.298301641	4.337552954	4.237429926
Infinium_Chinese	Type_2_diabetes	6.051894965	3.022199337	3.460018267	1.601919299	3.537121374	2.24562626
Infinium_JSA	Type_2_diabetes	7.054593393	3.517178948	5.013857395	3.190005099	3.841569749	3.77082546
Axiom_UKB	Type_2_diabetes	8.087960982	3.555382073	4.421375031	2.402681328	3.30920329	2.970088498
CytoSNP-850K	Type_2_diabetes	5.182172521	2.554626315	3.511589821	2.672632199	2.548082352	2.315995843
Axiom_PMRA	Type_2_diabetes	7.197868722	3.933260803	4.623330814	3.287234841	4.242621936	3.39683327
Axiom_PMDA	Type_2_diabetes	6.806722497	3.936582814	4.622937138	3.112932742	4.411155858	3.42439059
Affymetrix_6.0	Type_2_diabetes	6.121701635	3.134317202	5.142195767	3.026769799	3.59525094	3.753011621
OmniZhongHua	Type_2_diabetes	4.935445996	2.468254034	3.523793777	2.027991099	2.406313122	2.251334562
Multi-Ethnic_EUR_EAS_SAS	Type_2_diabetes	4.78438894	2.453304986	3.711577223	1.777407128	2.964607876	2.545804201
Multi-Ethnic_Global	Type_2_diabetes	4.785304437	2.59282944	4.002897455	1.682548842	3.23978237	2.553677721
Infinium_GDA	Type_2_diabetes	4.420021011	2.62355804	2.677784077	2.149725899	2.735853396	2.057350718
Axiom_GW_PanAFR	Type_2_diabetes	3.735229023	3.054588943	3.70016062	1.694010885	3.074175836	2.593635834
Infinium_Omni2.5	Type_2_diabetes	3.789472239	2.137713958	2.526218821	1.692429914	1.827108451	2.504566641
Infinium_Omni5	Type_2_diabetes	3.25642393	1.193432385	2.081758629	0.6782367426	1.244558194	1.715935217

Table S. 12 Mean absolute difference of percentile ranking between PGSs estimated from imputed genotyping data of 23 SNP arrays and PGS estimated from WGS in 6 different populations with PRsice p-value setting of 1e-6.

Array_name	Trait	AFR	AMR	EAS	EUR	SAS	VNP
CytoSNP-12	Height	11.73507339	7.949571876	8.784486017	7.13294784	8.201705413	6.273718978
Infinium_Core	Height	10.20207314	6.434734945	8.396715168	6.466568383	8.021043739	6.021667926
Infinium_OncoArray	Height	8.198736156	4.989660241	6.603127362	4.40893407	5.983581534	4.837982647
PsychArray	Height	8.725375983	5.227184014	6.826341648	5.057527598	6.684063717	5.452314027
Axiom_GW_ASI	Height	9.008264652	6.919748524	7.749905518	6.385148354	7.261595594	6.081506677
Infinium_GSA	Height	9.020623866	5.394115058	7.012944067	4.967412226	6.681972725	4.904612308
Axiom_GW_CHB	Height	7.820178018	5.36089495	6.291335979	5.554743112	6.409307422	4.854221781
Axiom_JAPONICA	Height	8.271518192	5.39162355	4.844970396	4.849234612	6.0417111	3.710297777
Axiom_GW_EUR	Height	10.20665063	6.178940112	7.903832829	5.663434898	7.521714948	6.233465608
Infinium_Chinese	Height	7.289189579	4.513782192	5.75042517	4.374943184	6.114059409	4.081042139
Infinium_JSA	Height	8.967525022	5.682299496	6.744457042	4.914449684	6.363305607	4.877054989
Axiom_UKB	Height	8.050425592	4.333563106	5.528785588	2.990802699	5.171022202	4.266069854
CytoSNP-850K	Height	5.682034052	3.562856597	4.379251701	3.259567841	3.836551369	3.109154541
Axiom_PMRA	Height	7.71924444	4.885847403	6.040170698	4.269808584	5.977308559	4.366063555
Axiom_PMDA	Height	7.813998412	4.959762144	6.310626102	3.935433127	5.981908741	4.352973828
Affymetrix_6.0	Height	6.382847242	4.377579749	5.871283699	4.38956717	5.33077396	4.386436287
OmniZhongHua	Height	4.915533929	3.135978208	3.323806374	2.51927797	3.708582684	2.685854434
Multi-Ethnic_EUR_EAS_SAS	Height	6.041137872	3.573653132	4.806783825	3.259567841	4.401955495	3.530486269
Multi-Ethnic_Global	Height	5.653424761	3.804532884	4.924492945	3.037836599	4.181564982	3.540229749
Infinium_GDA	Height	5.538300974	3.786261824	4.573333963	2.92321617	3.977066004	3.413958176
Axiom_GW_PanAFR	Height	4.360284811	4.217292727	4.140290375	3.74492607	4.536197155	3.235130858
Infinium_Omni2.5	Height	3.868662756	2.526389223	2.879346183	2.003881285	2.885150196	2.429079271
Infinium_Omni5	Height	3.07446884	1.923444261	2.327412446	1.443426914	2.148702958	2.092584719
CytoSNP-12	BMI	9.545432698	6.389057296	8.024297682	6.143654969	7.402528427	5.595021573

Infinium_Core	BMI	8.471096606	6.304346021	7.497952885	5.644858483	6.382124531	5.311870118
Infinium_OncoArray	BMI	7.441619881	4.586035928	6.313381834	4.393124355	5.08194596	4.590458869
PsychArray	BMI	7.28026348	4.748814457	6.310626102	4.727895055	5.537363929	4.785230064
Axiom_GW_ASI	BMI	7.477095402	5.100117101	6.221261653	5.906904497	5.824666173	5.094265716
Infinium_GSA	BMI	8.366958787	4.768746522	6.536989796	5.410874712	5.800410671	4.477277022
Axiom_GW_CHB	BMI	7.459929827	5.142472739	6.33542769	5.097051884	5.466688413	4.30376433
Axiom_JAPONICA	BMI	6.984786723	4.395020306	4.62175611	4.370990755	4.666675031	3.372425359
Axiom_GW_EUR	BMI	7.927291204	5.359233944	6.918068153	5.305344869	6.111550219	5.445719955
Infinium_Chinese	BMI	6.915666677	4.022955095	4.793792517	4.169416898	4.595163118	3.72073019
Infinium_JSA	BMI	8.044245985	5.27203116	6.366528093	5.109699655	5.334119546	4.37344498
Axiom_UKB	BMI	7.675987192	4.025446603	6.014581759	3.164709556	4.525323999	4.091080877
CytoSNP-850K	BMI	4.897910606	2.865234326	4.006046863	2.74614737	3.397861334	3.275088971
Axiom_PMRA	BMI	6.709222033	4.340207127	6.1106387	4.40616737	4.979905571	4.43229954
Axiom_PMDA	BMI	6.832356421	4.179089603	6.029935122	4.091949298	4.796316509	4.361733119
Affymetrix_6.0	BMI	5.430501166	4.179089603	5.5236678	4.027524713	4.227566797	4.075629094
OmniZhongHua	BMI	4.35914044	3.073690505	3.070672714	2.484891842	2.812801887	2.431047651
Multi-Ethnic_EUR_EAS_SAS	BMI	5.564850396	3.402569575	4.361929957	3.221229284	3.71945584	3.319180682
Multi-Ethnic_Global	BMI	5.293634318	3.253079089	4.286737843	3.12795197	3.541303357	3.313373961
Infinium_GDA	BMI	5.09748902	3.089470056	3.90526581	3.105027884	3.51077488	3.203144684
Axiom_GW_PanAFR	BMI	3.595386809	3.385959521	4.043839758	3.44335577	3.94821032	2.945975844
Infinium_Omni2.5	BMI	3.614383378	2.194188142	2.675422021	1.982142928	2.395021767	2.352017196
Infinium_Omni5	BMI	3.073553343	1.816309412	2.34276581	1.418526614	1.991042192	2.132444413
CytoSNP-12	Type_2_diabetes	11.71630569	5.690604523	7.734552154	6.464196926	7.686485085	5.480757118
Infinium_Core	Type_2_diabetes	10.90998144	5.328505344	6.640526581	5.560671755	6.686572907	5.276832955
Infinium_OncoArray	Type_2_diabetes	7.904403771	3.393434046	5.338640086	3.582086013	4.660820254	3.592293399
PsychArray	Type_2_diabetes	8.540216652	3.968141916	5.535084404	4.705761455	5.021307204	4.273156022
Axiom_GW_ASI	Type_2_diabetes	7.701392243	4.854288301	6.104339884	4.548454798	5.208660051	4.35966632

Infinium_GSA	Type_2_diabetes	8.110161791	4.691509771	5.217387881	4.429091455	4.811371649	3.861961451
Axiom_GW_CHB	Type_2_diabetes	8.713932267	4.711441836	6.105520912	5.212462798	5.764863814	4.258885267
Axiom_JAPONICA	Type_2_diabetes	8.293261253	3.653381392	3.793855505	3.567066784	4.525323999	2.850804674
Axiom_GW_EUR	Type_2_diabetes	9.127965925	4.183242116	5.444145251	3.529913956	4.357208275	4.175229119
Infinium_Chinese	Type_2_diabetes	6.64559497	2.963233645	3.67850844	2.612950527	3.450554322	2.457128685
Infinium_JSA	Type_2_diabetes	7.820635767	3.640923851	5.207939657	3.588409898	4.207911476	3.946897046
Axiom_UKB	Type_2_diabetes	8.652365073	3.777956797	4.615457294	2.812152927	3.480664601	3.207671958
CytoSNP-850K	Type_2_diabetes	5.688442533	2.748133445	3.724174855	2.727570956	2.6354858	2.773053666
Axiom_PMRA	Type_2_diabetes	7.244559085	4.007175543	4.669390905	3.789588513	4.463848846	3.350576342
Axiom_PMDA	Type_2_diabetes	7.287587459	3.787092327	4.846151424	3.72832587	4.748641901	3.618472852
Affymetrix_6.0	Type_2_diabetes	6.115293154	3.116876645	4.938271605	2.958788027	3.466445858	3.495842782
OmniZhongHua	Type_2_diabetes	5.314233008	2.345339634	3.498992189	2.405052785	2.691524375	2.456538171
Multi-Ethnic_EUR_EAS_SAS	Type_2_diabetes	5.403493995	2.050511174	3.667485513	2.146563956	2.972135446	2.747956822
Multi-Ethnic_Global	Type_2_diabetes	5.301873794	2.273085899	4.000535399	2.060796256	3.28452959	2.759963939
Infinium_GDA	Type_2_diabetes	5.064302242	2.495660623	2.979339884	2.601883727	2.938261382	2.353001386
Axiom_GW_PanAFR	Type_2_diabetes	4.031621277	2.915894991	3.954475309	2.394381228	3.369842046	2.679949295
Infinium_Omni2.5	Type_2_diabetes	4.038716381	1.947528839	2.61755165	1.773059456	1.763542307	2.296016786
Infinium_Omni5	Type_2_diabetes	3.413889467	1.302228239	2.220332577	1.343825714	1.276759465	1.657179075

Table S. 13 Mean absolute difference of percentile ranking between PGSs estimated from imputed genotyping data of 23 SNP arrays and PGS estimated from WGS in 6 different populations with PRsice p-value setting of 1e-5.

Array_name	Trait	AFR	AMR	EAS	EUR	SAS	VNP
CytoSNP-12	Height	11.81014417	7.533490022	8.843931091	6.94757894	8.256907591	6.122153723
Infinium_Core	Height	10.01668494	6.364142215	8.430964979	6.592255611	8.323819322	6.030624055
Infinium_OncoArray	Height	8.392134963	4.934847063	6.773195389	4.691137469	6.154624646	4.901758157
PsychArray	Height	8.802277757	5.291963225	7.060578861	5.262263398	6.833778715	5.685862308
Axiom_GW_ASI	Height	9.235307985	6.84500328	7.607001134	6.409653412	7.468603761	6.224115804
Infinium_GSA	Height	9.200519087	5.445606225	7.073570169	5.046856041	6.667335784	4.993583081
Axiom_GW_CHB	Height	7.93026657	5.304420766	6.338970773	5.432217826	6.567386386	4.946538801
Axiom_JAPONICA	Height	8.381148995	5.350928917	4.808752205	4.952392998	6.042965695	3.734016755
Axiom_GW_EUR	Height	10.1837632	6.148211512	7.753842278	5.614424783	7.554752615	6.280116213
Infinium_Chinese	Height	7.277516988	4.733034906	5.76026707	4.347276184	6.186825917	4.160072594
Infinium_JSA	Height	9.117208832	5.791925853	6.752724238	4.861091898	6.434817519	4.945456192
Axiom_UKB	Height	8.154563411	4.37176623	5.651612497	3.180124027	5.448705885	4.441550926
CytoSNP-850K	Height	5.61108301	3.479806327	4.467828798	3.17498587	3.975393211	3.147144274
Axiom_PMRA	Height	7.819491395	4.936508068	6.011038675	4.27889917	6.18849871	4.381416919
Axiom_PMDA	Height	7.504789195	4.854288301	6.431878307	3.954800027	6.201462858	4.398738662
Affymetrix_6.0	Height	6.357213318	4.367613717	5.938995969	4.436996312	5.556182853	4.406218506
OmniZhongHua	Height	4.901572596	3.062063467	3.327743134	2.50821117	3.795567934	2.684378149
Multi-Ethnic_EUR_EAS_SAS	Height	6.029007532	3.586941175	4.646557697	3.167871499	4.542051932	3.610402494
Multi-Ethnic_Global	Height	5.734675147	3.736431662	4.696554548	3.082894284	4.421192618	3.634121473
Infinium_GDA	Height	5.549973565	3.639262846	4.481607458	2.938630642	4.172364619	3.525368481
Axiom_GW_PanAFR	Height	4.195953044	4.213970716	4.180839002	3.690382556	4.659983858	3.241035998
Infinium_Omni2.5	Height	3.81441954	2.57455838	2.906903502	1.933528056	2.99848194	2.407919186
Infinium_Omni5	Height	3.073782217	1.944206828	2.360874906	1.426431471	2.181322427	2.085498551
CytoSNP-12	BMI	9.9956285	6.766105524	8.297902494	6.140888269	7.523387741	5.653187201

Infinium_Core	BMI	9.135747652	6.070144258	7.488504661	6.143654969	6.555676833	5.439519558
Infinium_OncoArray	BMI	7.650353268	4.599323971	6.193310658	4.716828255	5.334955943	4.724210286
PsychArray	BMI	7.734807894	5.054439452	6.593285462	4.880063555	5.616821609	4.779324924
Axiom_GW_ASI	BMI	7.738927632	4.940660582	6.569664903	6.212031983	6.155042844	5.031179138
Infinium_GSA	BMI	8.596519737	4.801136128	6.52281746	5.165033655	5.867322402	4.699703956
Axiom_GW_CHB	BMI	7.706885226	4.867576344	6.183075082	5.310878269	5.766954805	4.294119268
Axiom_JAPONICA	BMI	7.148660742	4.491358619	4.432004283	4.560707327	4.712676846	3.319082263
Axiom_GW_EUR	BMI	8.106728676	5.245455074	7.212537793	5.297835255	6.270883779	5.317480001
Infinium_Chinese	BMI	7.220527281	4.12593743	5.038265306	4.402214941	4.700549094	3.910482017
Infinium_JSA	BMI	8.174475477	5.240472058	6.572026959	5.452375212	5.738935518	4.367736678
Axiom_UKB	BMI	7.838945713	3.948209851	6.172052154	3.422407899	4.698458103	4.143636621
CytoSNP-850K	BMI	4.937734739	3.067046483	4.087144117	3.007798142	3.644598341	3.166532817
Axiom_PMRA	BMI	7.354647637	4.564442857	6.211026077	4.757538269	5.177713375	4.382007433
Axiom_PMDA	BMI	7.069470225	4.057836208	6.429122575	4.079301527	5.048490095	4.385058422
Affymetrix_6.0	BMI	5.734904022	4.18739463	5.431941295	4.050844041	4.332116376	4.17030817
OmniZhongHua	BMI	4.404457556	2.855268294	3.204128874	2.757609413	2.840402976	2.44285793
Multi-Ethnic_EUR_EAS_SAS	BMI	5.440800511	3.319519305	4.55601222	3.331502041	3.684327182	3.350084247
Multi-Ethnic_Global	BMI	5.586135709	3.214875964	4.354056437	3.282491927	3.538375969	3.391223388
Infinium_GDA	BMI	5.417913078	2.945793089	4.047382842	3.13625207	3.51077488	3.188677091
Axiom_GW_PanAFR	BMI	3.624224974	3.429145662	4.000929075	3.553233284	3.892171746	3.033371914
Infinium_Omni2.5	BMI	3.55258731	2.210798196	2.68526392	2.056843828	2.305109129	2.357823917
Infinium_Omni5	BMI	3.228272388	1.749869196	2.439216427	1.575042785	2.007351926	2.051347159
CytoSNP-12	Type_2_diabetes	10.8024105	5.713858599	7.717230411	6.434158469	7.218102969	5.440405329
Infinium_Core	Type_2_diabetes	10.52890568	4.90328796	6.627141597	5.173728998	6.774394553	4.978524975
Infinium_OncoArray	Type_2_diabetes	7.824068882	3.515517943	5.416981607	3.405017213	5.132129759	3.840014015
PsychArray	Type_2_diabetes	8.489406552	3.854363046	5.677988788	4.267041884	5.491780312	4.488398369
Axiom_GW_ASI	Type_2_diabetes	7.859086654	4.852627295	5.882700302	4.615646084	5.298990887	4.347265527

Infinium_GSA	Type_2_diabetes	8.211324244	4.15666603	6.079538297	4.602998312	5.002070082	4.401986489
Axiom_GW_CHB	Type_2_diabetes	8.55280474	4.463121527	6.029541446	5.253568055	5.488434726	4.175425957
Axiom_JAPONICA	Type_2_diabetes	7.696357007	3.548738051	4.457593222	4.017248398	4.59725411	2.992823287
Axiom_GW_EUR	Type_2_diabetes	8.560586468	4.164140554	5.838608592	3.82239367	4.544142923	4.22522597
Infinium_Chinese	Type_2_diabetes	6.57258406	3.237299537	4.311145755	3.201467141	3.78678577	3.020282187
Infinium_JSA	Type_2_diabetes	7.957273741	3.83110897	5.699640967	3.990371884	4.478485788	4.141176146
Axiom_UKB	Type_2_diabetes	7.988629523	3.537111013	4.965041572	2.595559842	3.469373246	3.687858245
CytoSNP-850K	Type_2_diabetes	5.654340258	2.752285959	4.058799446	2.781323985	3.002663923	2.96890747
Axiom_PMRA	Type_2_diabetes	7.114558467	4.04620917	5.289036911	3.90539467	4.857791662	3.480587837
Axiom_PMDA	Type_2_diabetes	7.287816333	3.836922489	5.183925422	3.77812647	4.935576549	3.893160273
Affymetrix_6.0	Type_2_diabetes	5.771981663	2.898454434	5.006377551	3.17775257	3.899281117	3.500566893
OmniZhongHua	Type_2_diabetes	5.111221479	2.330390585	3.830467372	2.248536613	2.490789182	2.414513259
Multi-Ethnic_EUR_EAS_SAS	Type_2_diabetes	5.091767162	2.188374623	3.800941673	2.420072013	3.122268642	3.076479434
Multi-Ethnic_Global	Type_2_diabetes	5.051256406	2.333712596	4.281226379	2.243398456	3.198380736	3.041442271
Infinium_GDA	Type_2_diabetes	4.683684236	2.549643299	3.435216679	2.976969199	2.809874499	2.647274187
Axiom_GW_PanAFR	Type_2_diabetes	3.976004816	3.068707489	4.481213782	2.446158042	3.733674583	2.633397109
Infinium_Omni2.5	Type_2_diabetes	3.751936849	2.01230805	2.971466364	1.730373228	2.016134091	2.401817208
Infinium_Omni5	Type_2_diabetes	3.169222811	1.728276126	2.673847317	1.243829271	1.59375379	1.837482678

Table S. 14 Mean absolute difference of percentile ranking between PGSs estimated from imputed genotyping data of 23 SNP arrays and PGS estimated from WGS in 6 different populations with PRsice p-value setting of 1e-4.

Array_name	Trait	AFR	AMR	EAS	EUR	SAS	VNP
CytoSNP-12	Height	11.49292435	7.722844638	8.911643361	7.012398768	8.381948888	6.307575113
Infinium_Core	Height	10.36686266	6.49453114	8.529777652	6.56300764	8.2297247	6.174315791
Infinium_OncoArray	Height	8.45072679	5.242963566	6.990504535	4.605369769	6.301830454	5.049878748
PsychArray	Height	9.114233466	5.655723409	7.04601285	5.282025541	6.917836576	5.893329554
Axiom_GW_ASI	Height	9.24720945	6.92473154	7.775888133	6.271318412	7.405874014	6.237697625
Infinium_GSA	Height	9.069145223	5.285319204	7.058216805	4.869391998	6.595405673	5.199770881
Axiom_GW_CHB	Height	7.9201961	5.458063766	6.266928068	5.519566498	6.444017882	5.04869772
Axiom_JAPONICA	Height	8.468121239	5.216387479	4.6875	4.973736112	5.941761702	3.903887944
Axiom_GW_EUR	Height	10.3103307	6.237075302	8.099883472	5.446446569	7.530497112	6.428925737
Infinium_Chinese	Height	7.310017143	4.790339593	5.900809398	4.25953227	6.073494172	4.41271416
Infinium_JSA	Height	9.076698076	5.891586177	6.810200932	4.867020541	6.371251375	5.10361552
Axiom_UKB	Height	8.089791976	4.365122208	5.776014109	3.158780913	5.36799361	4.492925642
CytoSNP-850K	Height	5.630308454	3.601059721	4.449326027	3.250872499	4.006758085	3.277451027
Axiom_PMRA	Height	7.916762985	5.068557998	6.292517007	4.186412341	6.022892176	4.58642369
Axiom_PMDA	Height	7.477324276	4.998795771	6.457467246	3.790774241	6.129532747	4.553453326
Affymetrix_6.0	Height	6.397495199	4.439036949	6.054736709	4.464663312	5.515199418	4.582290092
OmniZhongHua	Height	4.910956443	3.108571618	3.383251449	2.523625642	3.547158133	2.786832326
Multi-Ethnic_EUR_EAS_SAS	Height	6.118497394	3.802041376	4.791430461	3.191981313	4.409901263	3.588848734
Multi-Ethnic_Global	Height	5.81043255	3.973955435	4.735922147	3.049298642	4.32877079	3.626247953
Infinium_GDA	Height	5.5719455	3.935752311	4.557586924	2.874206056	4.029340794	3.531470459
Axiom_GW_PanAFR	Height	4.216551734	4.189055635	4.346182918	3.633862827	4.572998607	3.345261716
Infinium_Omni2.5	Height	3.782377135	2.572897375	2.867142227	1.891237071	3.002245725	2.543245307
Infinium_Omni5	Height	3.079046326	2.024765591	2.362449609	1.366354557	2.241542985	2.159312799
CytoSNP-12	BMI	11.01251714	6.772749545	8.495527841	6.738890711	7.367399768	5.92511889

Infinium_Core	BMI	9.189761994	5.956365388	7.478269085	6.587512697	6.509675018	5.73566232
Infinium_OncoArray	BMI	8.147239432	4.649154133	6.3893613	5.050808469	5.364229825	4.985315886
PsychArray	BMI	8.201482648	4.961423149	6.68973608	5.343683426	5.771136788	5.027931311
Axiom_GW_ASI	BMI	7.714438079	5.178184355	6.3992032	6.199384212	5.660732433	5.122216711
Infinium_GSA	BMI	8.544107516	5.032015879	6.788548753	5.552371655	6.000727665	4.744976694
Axiom_GW_CHB	BMI	7.774174279	4.59600196	6.239370748	5.819951069	5.526490772	4.704920163
Axiom_JAPONICA	BMI	7.118449331	4.117632403	4.624511842	4.823939069	4.774988395	3.417009165
Axiom_GW_EUR	BMI	8.005337349	4.876711874	7.012156715	5.421546269	5.955980445	5.386865394
Infinium_Chinese	BMI	7.23792173	4.049531181	5.098497732	4.633432012	4.476394796	4.100922777
Infinium_JSA	BMI	8.647329838	5.553571577	6.417705971	5.597824583	5.559946638	4.776864449
Axiom_UKB	BMI	7.694983761	3.821973441	6.005527211	3.70065887	4.447539112	4.430429579
CytoSNP-850K	BMI	5.235500239	2.986487721	3.880464223	3.237434241	3.709837279	3.261211892
Axiom_PMRA	BMI	7.409577475	4.428240414	5.9614355	4.809315084	5.025907386	4.514971498
Axiom_PMDA	BMI	7.279119108	3.955684376	6.153155707	4.448458355	5.353356669	4.61093002
Affymetrix_6.0	BMI	5.836524223	3.980599457	5.372889897	4.313680541	4.448793707	4.273844955
OmniZhongHua	BMI	4.506993255	2.787167072	3.241921769	2.966297642	2.91651507	2.654754031
Multi-Ethnic_EUR_EAS_SAS	BMI	5.619322486	3.165876305	4.63356639	3.52635677	3.780094596	3.536391408
Multi-Ethnic_Global	BMI	5.698741878	3.15507977	4.424918115	3.582876498	3.73451098	3.577333711
Infinium_GDA	BMI	5.537614351	2.944962586	4.190287226	3.278934741	3.515375061	3.447026959
Axiom_GW_PanAFR	BMI	3.912606627	3.303739754	3.889912446	3.744530827	3.742456748	3.228930461
Infinium_Omni2.5	BMI	3.747817111	2.260628358	2.817932729	2.177392899	2.241542985	2.458801808
Infinium_Omni5	BMI	3.22941676	1.701700039	2.437248047	1.594014442	2.021570669	2.061779573
CytoSNP-12	Type_2_diabetes	10.71543826	5.731299155	8.506550768	6.607670083	7.747542039	5.843332703
Infinium_Core	Type_2_diabetes	10.0981642	5.034507387	7.74281935	5.765407555	7.122335554	5.035214317
Infinium_OncoArray	Type_2_diabetes	7.994122507	4.136733965	6.561004031	4.193921955	5.576256372	4.174737024
PsychArray	Type_2_diabetes	8.68921384	4.303665008	6.968852356	4.687580284	6.403870844	4.740252583
Axiom_GW_ASI	Type_2_diabetes	7.604578402	4.439036949	6.368890149	4.993893498	5.318646208	4.438598356

Infinium_GSA	Type_2_diabetes	8.409071663	4.418274381	7.066484001	4.963459798	6.058020835	4.608371126
Axiom_GW_CHB	Type_2_diabetes	8.183401576	4.351003663	6.621630134	5.502571055	5.998218475	4.475800737
Axiom_JAPONICA	Type_2_diabetes	7.960249107	3.528805986	4.898904006	4.480473027	4.96275944	3.139566012
Axiom_GW_EUR	Type_2_diabetes	8.240391284	4.07361576	6.658635676	4.109339984	5.13171156	4.647345049
Infinium_Chinese	Type_2_diabetes	6.646739342	3.287960202	5.215813177	3.69512547	4.246385721	3.312881866
Infinium_JSA	Type_2_diabetes	8.527857439	4.369274722	6.419674351	4.657541827	5.934234133	4.550205499
Axiom_UKB	Type_2_diabetes	7.713293708	3.753041716	5.637833837	3.15561897	4.284859966	3.894734977
CytoSNP-850K	Type_2_diabetes	5.620924607	2.79879411	4.082813681	2.868672656	3.469373246	2.969301146
Axiom_PMRA	Type_2_diabetes	6.868976314	3.891735668	5.893329554	4.385219498	5.258843849	3.802024282
Axiom_PMDA	Type_2_diabetes	6.871493931	3.935752311	5.581931847	4.062306084	5.197786895	4.139896699
Affymetrix_6.0	Type_2_diabetes	5.508776186	3.177503343	5.588624339	3.275772799	4.00048511	3.713447184
OmniZhongHua	Type_2_diabetes	4.730374599	2.294678969	3.787950365	2.294780028	2.938679581	2.509487591
Multi-Ethnic_EUR_EAS_SAS	Type_2_diabetes	5.251750316	2.532202742	4.567035147	2.584493042	3.533357589	3.141140716
Multi-Ethnic_Global	Type_2_diabetes	5.128844803	2.44250845	4.684350592	2.506630199	3.668017447	3.160922934
Infinium_GDA	Type_2_diabetes	4.655761568	2.529711234	4.382794785	3.119256627	3.306275902	2.900014172
Axiom_GW_PanAFR	Type_2_diabetes	3.752165723	2.832014218	4.477670698	2.760376113	3.91099067	2.855627205
Infinium_Omni2.5	Type_2_diabetes	3.541143593	1.979918445	3.152163643	1.901908628	2.194286575	2.413233812
Infinium_Omni5	Type_2_diabetes	3.067144861	1.725784617	2.645896321	1.374259414	1.829199443	1.891711546

Table S. 15 Mean absolute difference of percentile ranking between PGSs estimated from imputed genotyping data of 23 SNP arrays and PGS estimated from WGS in 6 different populations with PRsice p-value setting of 1e-3.

Array_name	Trait	AFR	AMR	EAS	EUR	SAS	VNP
CytoSNP-12	Height	11.45493121	7.373203	9.032895566	6.992241383	8.468097741	6.502838404
Infinium_Core	Height	10.13066435	6.482073599	8.728584026	6.532178697	8.196687033	6.176874685
Infinium_OncoArray	Height	8.519846837	5.243794069	7.508582137	4.593907727	6.427289949	5.076451877
PsychArray	Height	9.160694954	5.630808328	7.469608214	5.131042769	6.794886271	5.900317303
Axiom_GW_ASI	Height	9.064109988	6.557649345	7.562515747	6.122311855	7.640065072	6.117921706
Infinium_GSA	Height	9.162297074	5.397437069	7.079868985	4.771767012	6.484583119	5.189830562
Axiom_GW_CHB	Height	8.149299301	5.482148344	6.396053792	5.375302855	6.715428591	5.069660966
Axiom_JAPONICA	Height	8.519389089	5.101778106	4.947326153	4.589955298	6.129532747	3.907529447
Axiom_GW_EUR	Height	10.27073544	5.998721026	8.158541194	5.315621183	7.631701105	6.353635204
Infinium_Chinese	Height	7.418961323	4.694831782	5.949231544	4.224355655	6.090640303	4.444798753
Infinium_JSA	Height	9.2387411	5.99706002	7.005857899	4.967807469	6.500474655	5.042694161
Axiom_UKB	Height	8.235584923	3.974785938	5.707908163	3.102656427	5.386394336	4.507491654
CytoSNP-850K	Height	5.687298161	3.669991446	4.600497606	3.131113913	4.187419758	3.238378685
Axiom_PMRA	Height	7.947889893	4.948135106	6.302752583	4.137402227	6.219445385	4.495779793
Axiom_PMDA	Height	7.551937307	4.616764528	6.495260141	3.947685655	6.05425705	4.565952538
Affymetrix_6.0	Height	6.324484289	4.432392927	6.002771479	4.263879941	5.562037629	4.386042611
OmniZhongHua	Height	4.892875371	3.199096413	3.540328168	2.401100356	3.684327182	2.802677784
Multi-Ethnic_EUR_EAS_SAS	Height	6.086683863	3.448247224	4.91819413	3.066689327	4.591817532	3.602233718
Multi-Ethnic_Global	Height	6.037704757	3.772143278	4.96425422	2.928354327	4.680893773	3.577136873
Infinium_GDA	Height	5.572860998	3.604381732	4.837096876	2.809386227	4.264368249	3.389255008
Axiom_GW_PanAFR	Height	4.249509637	4.051192187	4.522549761	3.395136141	4.557107071	3.233359316
Infinium_Omni2.5	Height	3.856761291	2.60528698	3.054138322	1.837484042	2.927806424	2.573263102
Infinium_Omni5	Height	3.166705194	1.955003364	2.443940539	1.269915299	2.218960275	2.166005291
CytoSNP-12	BMI	11.01366151	7.090832081	9.172256866	7.197767668	7.405874014	6.095088498

Infinium_Core	BMI	9.823286132	6.618276042	8.24829932	7.015955954	6.635552712	5.926595175
Infinium_OncoArray	BMI	8.611396568	5.652401399	7.120023936	5.704935398	5.708825239	5.218962585
PsychArray	BMI	8.917859293	5.29943775	7.561728395	6.02350114	5.988599914	5.258625441
Axiom_GW_ASI	BMI	7.79751946	5.314386798	6.906257874	6.461430226	5.926706563	5.163847947
Infinium_GSA	BMI	9.078300196	5.308573279	7.33064059	5.922714212	6.18348033	4.826369205
Axiom_GW_CHB	BMI	8.06621792	5.15825229	6.744063366	6.199779454	6.067639396	4.972029321
Axiom_JAPONICA	BMI	7.372957583	4.684035247	5.11148904	5.273725441	5.1467667	3.578514739
Axiom_GW_EUR	BMI	8.072168653	5.267048144	7.438114135	5.791493583	6.12869635	5.369445232
Infinium_Chinese	BMI	7.704825357	4.542019284	5.710270219	4.869391998	4.907557262	4.243039809
Infinium_JSA	BMI	8.81829896	5.683129999	7.147581255	6.132588169	6.079767147	5.011003244
Axiom_UKB	BMI	8.043101613	4.619256036	6.252755732	3.857965527	4.980741967	4.485052123
CytoSNP-850K	BMI	5.407613733	3.475653813	4.668603553	3.639000984	3.702727908	3.282568815
Axiom_PMRA	BMI	7.555370422	5.180675863	6.88696775	4.878877826	5.471288594	4.615457294
Axiom_PMDA	BMI	7.846269692	4.478070576	6.662966112	4.829077227	5.403958665	4.594690886
Affymetrix_6.0	BMI	6.119184017	4.265461884	5.728772991	4.488773127	4.731913968	4.289395156
OmniZhongHua	BMI	4.885322518	3.204909932	3.707640464	3.221624527	3.091740165	2.703471435
Multi-Ethnic_EUR_EAS_SAS	BMI	5.786400745	3.296265229	4.807571177	3.893537384	4.051087107	3.599576405
Multi-Ethnic_Global	BMI	5.932880315	3.254740094	4.668603553	4.031081898	3.883389581	3.481965703
Infinium_GDA	BMI	5.655255756	2.971538672	4.62766125	3.85006067	3.95824708	3.404116276
Axiom_GW_PanAFR	BMI	3.888574822	3.464857278	4.444995591	4.230284298	3.796404331	3.346344325
Infinium_Omni2.5	BMI	3.8620254	2.452474483	3.125393676	2.435881728	2.304690931	2.307531809
Infinium_Omni5	BMI	3.440438889	1.836241477	2.548658352	1.698753799	2.122774662	2.082841238
CytoSNP-12	Type_2_diabetes	10.83147754	6.112499896	8.72228521	6.76497674	8.072482132	6.1204806
Infinium_Core	Type_2_diabetes	9.649570517	5.180675863	7.8026581	5.85749914	7.478222323	5.696196303
Infinium_OncoArray	Type_2_diabetes	8.37611376	4.555307328	6.773589065	4.763862155	5.998636673	4.793202003
PsychArray	Type_2_diabetes	8.980570858	4.703967311	7.352292769	5.112861598	6.763939595	5.236973262
Axiom_GW_ASI	Type_2_diabetes	7.371355462	4.277088922	5.629566641	5.073732555	5.413159028	4.256031116

Infinium_GSA	Type_2_diabetes	8.732013339	4.674899717	7.390085664	5.319968855	6.287611711	4.803043903
Axiom_GW_CHB	Type_2_diabetes	8.499477022	4.539527776	6.698790627	5.689520926	6.336540914	4.594789305
Axiom_JAPONICA	Type_2_diabetes	7.857255659	3.968972419	4.760330058	4.714456798	5.213678431	3.387286628
Axiom_GW_EUR	Type_2_diabetes	8.218419348	4.215631722	6.200003149	3.962309641	5.23124276	4.645770345
Infinium_Chinese	Type_2_diabetes	7.143396632	3.567009111	5.580357143	4.04372967	4.588053747	3.948570169
Infinium_JSA	Type_2_diabetes	9.027947844	4.938999576	6.837758251	4.936583284	6.584532517	4.992795729
Axiom_UKB	Type_2_diabetes	7.962766724	4.012989062	5.750031494	3.355611856	4.641583132	4.058110513
CytoSNP-850K	Type_2_diabetes	5.658459996	2.982335208	4.444601915	3.079337099	3.670108439	3.339454995
Axiom_PMRA	Type_2_diabetes	7.148660742	4.155835527	5.722474175	4.314075784	5.998636673	4.30081176
Axiom_PMDA	Type_2_diabetes	7.198097597	3.964819905	5.97718254	4.193921955	5.642749905	4.329156431
Affymetrix_6.0	Type_2_diabetes	5.606505524	3.149266251	5.245732552	3.273796584	4.657474668	3.870031809
OmniZhongHua	Type_2_diabetes	4.799036897	2.391017283	3.878889519	2.519673213	3.100940528	2.876393613
Multi-Ethnic_EUR_EAS_SAS	Type_2_diabetes	5.519075531	2.758099478	4.232410557	2.929935299	4.067396841	3.526746347
Multi-Ethnic_Global	Type_2_diabetes	5.321328112	2.59282944	4.476883346	2.797924185	4.10503469	3.546626984
Infinium_GDA	Type_2_diabetes	5.474902786	2.770557018	4.616244646	3.184076456	3.700636916	3.44200759
Axiom_GW_PanAFR	Type_2_diabetes	3.864543018	2.881013878	4.128480096	2.93704967	3.941937346	2.959951342
Infinium_Omni2.5	Type_2_diabetes	3.787183495	2.094527818	3.019101159	2.004671771	2.530518022	2.485473356
Infinium_Omni5	Type_2_diabetes	3.182726397	1.602870217	2.512833837	1.400740685	1.999824357	1.98648904

Table S. 16 Mean absolute difference of percentile ranking between PGSs estimated from imputed genotyping data of 23 SNP arrays and PGS estimated from WGS in 6 different populations with PRsice p-value setting of 0.01.

Array_name	Trait	AFR	AMR	EAS	EUR	SAS	VNP
CytoSNP-12	Height	11.4077831	7.849911551	9.133282943	6.800153354	8.288272465	6.621630134
Infinium_Core	Height	10.3460351	6.531073259	8.847474175	6.474868483	8.318382743	6.454022581
Infinium_OncoArray	Height	8.759936007	5.371691485	7.465277778	4.604579284	6.664408396	5.534789147
PsychArray	Height	9.360502242	5.737112674	7.552280171	5.127090341	7.126517537	6.133668745
Axiom_GW_ASI	Height	8.899320472	6.105025372	7.637707861	5.926271397	7.434729698	6.140853332
Infinium_GSA	Height	9.210818432	5.162404804	6.6779258	4.861487141	6.405125439	5.493157911
Axiom_GW_CHB	Height	8.131904852	5.658214917	6.583049887	5.151595398	6.703719038	5.324369331
Axiom_JAPONICA	Height	8.390075094	5.266217642	4.865441547	4.518021098	6.026237762	3.911859883
Axiom_GW_EUR	Height	9.883022331	5.880789642	8.003039179	5.293882826	7.502059627	6.519274376
Infinium_Chinese	Height	7.529507623	5.095964587	5.926398337	4.435020098	5.998218475	4.768203578
Infinium_JSA	Height	9.57198212	6.482073599	6.8074452	4.876111126	6.813286997	5.409009669
Axiom_UKB	Height	8.141517574	4.043717662	5.830341396	3.092380113	5.364229825	4.762888952
CytoSNP-850K	Height	5.778619018	3.562856597	4.792217813	3.222415013	4.215439046	3.429508377
Axiom_PMRA	Height	8.132133727	5.274522669	6.516124969	4.074558613	6.183062132	4.716238347
Axiom_PMDA	Height	7.413926087	4.770407528	6.503527337	3.991557613	6.080185345	4.766235198
Affymetrix_6.0	Height	6.338216749	4.664933684	6.231103553	4.027919955	5.463342826	4.517825649
OmniZhongHua	Height	4.92720652	3.088639554	3.535997732	2.590421685	3.689345561	2.914481765
Multi-Ethnic_EUR_EAS_SAS	Height	6.185557572	3.508873921	5.05165029	3.118070899	4.754078479	3.827317964
Multi-Ethnic_Global	Height	5.919147855	3.605212235	5.034328546	3.015702999	4.797152906	3.757637314
Infinium_GDA	Height	5.806083937	3.544585538	5.094167297	2.955230842	4.477649391	3.627625819
Axiom_GW_PanAFR	Height	4.375161642	3.955684376	4.542627236	3.462327427	4.746550909	3.331679894
Infinium_Omni2.5	Height	3.900247413	2.444999958	3.011227639	1.972261856	2.945370754	2.616764298
Infinium_Omni5	Height	3.284117724	1.821292428	2.520313681	1.322482599	2.303854534	2.203896605
CytoSNP-12	BMI	11.24436683	6.453836507	9.726552658	7.713559597	7.701540224	6.733138857

Infinium_Core	BMI	9.665133972	6.37493875	8.474269337	7.382741325	7.629191915	6.577341585
Infinium_OncoArray	BMI	8.539072281	5.467199296	7.526691232	5.87133264	6.271720175	5.668638983
PsychArray	BMI	9.00551816	5.484639853	8.25223608	6.48277334	6.666499387	5.680646101
Axiom_GW_ASI	BMI	7.912643247	4.889169414	7.031053162	6.317166583	6.37752435	5.421508881
Infinium_GSA	BMI	9.367368472	5.235489042	7.643613001	5.820741555	6.545221875	5.343954712
Axiom_GW_CHB	BMI	7.915847487	4.990490744	7.032627866	6.401748554	6.269210985	5.311968537
Axiom_JAPONICA	BMI	7.421707814	4.329410592	5.208333333	5.406922283	5.669932796	3.890404541
Axiom_GW_EUR	BMI	7.96253785	4.922389522	7.190098262	5.750388326	6.350341459	5.942932729
Infinium_Chinese	BMI	7.449630482	4.515443198	6.254330436	5.082427898	5.431141556	4.715352576
Infinium_JSA	BMI	9.182438015	5.409064107	7.625110229	6.61280824	6.839215293	5.460679642
Axiom_UKB	BMI	8.134880219	3.936582814	6.648400101	3.821207941	4.712676846	4.731591711
CytoSNP-850K	BMI	5.556839795	3.36104444	4.733953767	3.765083456	4.086633964	3.593671265
Axiom_PMRA	BMI	7.46748268	4.607628998	7.207420005	4.937769012	5.914160613	5.012085853
Axiom_PMDA	BMI	7.514401917	4.12593743	7.110969388	4.848444126	5.474215983	5.176937673
Affymetrix_6.0	BMI	5.905873144	4.036243138	6.242520156	4.921168812	5.158476253	4.726277085
OmniZhongHua	BMI	4.80658975	2.624388542	3.878889519	3.351659427	3.513702268	3.022545824
Multi-Ethnic_EUR_EAS_SAS	BMI	5.933109189	3.254740094	5.052043966	3.717654313	4.415337842	3.835486741
Multi-Ethnic_Global	BMI	5.935397932	3.056249948	4.932760141	3.924366327	4.077433601	3.749271699
Infinium_GDA	BMI	5.971560076	3.01389431	5.31462585	3.765083456	4.302424296	3.797792265
Axiom_GW_PanAFR	BMI	3.966163219	3.383468013	4.543414588	4.195502927	4.059451073	3.633727797
Infinium_Omni2.5	BMI	3.771848915	2.306306007	3.400966868	2.493982428	2.589483985	2.601017259
Infinium_Omni5	BMI	3.312955889	1.710835569	2.645896321	1.702706228	2.175049452	2.250645629
CytoSNP-12	Type_2_diabetes	10.51082461	6.047720685	8.993527967	7.557833911	8.655032389	6.124318941
Infinium_Core	Type_2_diabetes	10.20916825	6.012839572	8.686854371	6.844025311	7.967932553	5.538922745
Infinium_OncoArray	Type_2_diabetes	8.926785391	4.550324311	6.573207987	5.837341755	6.26293801	5.066216301
PsychArray	Type_2_diabetes	8.85057024	5.291963225	7.704239103	6.122707097	7.795216648	5.222899345
Axiom_GW_ASI	Type_2_diabetes	7.321918608	4.439036949	5.983875031	5.490713769	5.545309697	4.557586924

Infinium_GSA	Type_2_diabetes	8.482082573	4.670747203	7.427484883	5.812441455	6.789031495	4.918686224
Axiom_GW_CHB	Type_2_diabetes	8.077432762	4.947304603	7.182618418	5.73576434	6.490437895	4.946735639
Axiom_JAPONICA	Type_2_diabetes	7.918593979	4.087734306	5.064247921	4.991126798	5.384303344	3.672996977
Axiom_GW_EUR	Type_2_diabetes	7.836885844	4.017972078	6.137802343	4.41723417	5.783682738	4.67372134
Infinium_Chinese	Type_2_diabetes	7.391038655	3.931599797	5.744126354	4.738566612	5.23124276	4.144325554
Infinium_JSA	Type_2_diabetes	9.030236587	4.994643258	7.094828672	5.991881712	7.190920078	5.267876827
Axiom_UKB	Type_2_diabetes	8.12915836	4.303665008	6.338577098	3.806979198	5.193604911	4.30868528
CytoSNP-850K	Type_2_diabetes	5.788918363	3.282977186	4.577664399	3.517661427	4.161491462	3.374098482
Axiom_PMRA	Type_2_diabetes	7.070614596	4.428240414	6.246850592	4.914054441	6.068893991	4.256326373
Axiom_PMDA	Type_2_diabetes	7.073589962	4.401664327	6.358260897	4.691137469	5.393921906	4.519203515
Affymetrix_6.0	Type_2_diabetes	5.677914314	3.559534586	5.245732552	3.711330427	4.836045349	3.879676871
OmniZhongHua	Type_2_diabetes	4.863350583	2.762251991	3.745827035	3.099099242	3.384060789	2.849328389
Multi-Ethnic_EUR_EAS_SAS	Type_2_diabetes	5.881154717	3.2871297	4.806390149	3.567066784	4.414501445	3.44299178
Multi-Ethnic_Global	Type_2_diabetes	5.379462191	3.092792067	4.926067649	3.480903841	4.206238683	3.579794186
Infinium_GDA	Type_2_diabetes	5.344902168	3.052927937	4.848907155	3.711330427	4.179055792	3.451947909
Axiom_GW_PanAFR	Type_2_diabetes	3.905282648	3.095283575	4.24382716	3.281701441	3.843660741	2.904246189
Infinium_Omni2.5	Type_2_diabetes	3.785352501	2.115290385	3.19861741	2.244584185	2.673960045	2.464018015
Infinium_Omni5	Type_2_diabetes	3.318448873	1.812156899	2.682901864	1.754878285	2.167521882	2.07683768

Table S. 17 Mean absolute difference of percentile ranking between PGSs estimated from imputed genotyping data of 23 SNP arrays and PGS estimated from WGS in 6 different populations with PRsice p-value setting of 0.1.

Array_name	Trait	AFR	AMR	EAS	EUR	SAS	VNP
CytoSNP-12	Height	11.76871791	7.777657816	9.366339128	6.402934283	8.534173076	6.951038517
Infinium_Core	Height	10.80836124	6.274447923	9.473812673	6.182784012	8.582265882	6.787367725
Infinium_OncoArray	Height	9.112402471	5.404911593	7.996740363	4.359528712	7.198865846	5.924626795
PsychArray	Height	9.393460145	6.052703702	8.010125346	5.127880826	7.644247055	6.368693311
Axiom_GW_ASI	Height	8.730640093	5.837603501	7.59558453	5.367397998	7.247376851	6.046174257
Infinium_GSA	Height	9.237825602	5.389962544	7.098371756	4.567821698	6.746793464	5.772471025
Axiom_GW_CHB	Height	8.536554663	5.672333463	6.769652305	5.038951184	6.841306284	5.707416068
Axiom_JAPONICA	Height	8.586449267	5.256251609	4.841427312	4.232655755	6.1412423	4.127692744
Axiom_GW_EUR	Height	9.569693377	5.575164647	8.312862182	5.003774569	7.069642566	6.594269652
Infinium_Chinese	Height	7.734350146	4.911592987	6.173233182	4.405376884	6.343650286	4.920457766
Infinium_JSA	Height	9.708162345	6.320125572	7.281037415	4.830262955	6.796140866	5.662044911
Axiom_UKB	Height	8.111535037	3.944887841	5.857111363	2.80701477	5.418177408	5.00647597
CytoSNP-850K	Height	5.828284747	3.847719024	4.668603553	2.967088127	4.496050117	3.73254047
Axiom_PMRA	Height	8.184317073	5.232167031	6.552736835	3.77535977	6.316467395	4.930299666
Axiom_PMDA	Height	7.436355771	4.687357257	6.968852356	3.942942741	6.269210985	5.082553855
Affymetrix_6.0	Height	6.676035256	4.640018603	6.414556563	3.883656313	5.807520042	4.83867158
OmniZhongHua	Height	5.129989174	3.214875964	3.68638196	2.33390907	3.864152458	3.09065177
Multi-Ethnic_EUR_EAS_SAS	Height	6.0569302	3.640093349	5.372496221	2.972621527	4.68423936	4.156627929
Multi-Ethnic_Global	Height	5.785027499	3.666669435	5.23904006	2.899106356	4.903375279	4.048760708
Infinium_GDA	Height	5.785485248	3.583619165	5.170934114	2.771838156	4.522814809	3.927410084
Axiom_GW_PanAFR	Height	4.232572937	3.944057338	4.521762409	3.287630084	4.838554539	3.576152683
Infinium_Omni2.5	Height	3.79748284	2.641829099	3.165154951	1.973447585	2.935752192	2.770396353
Infinium_Omni5	Height	3.292814948	1.847868515	2.531336609	1.332758914	2.26998047	2.30743339
CytoSNP-12	BMI	11.94014479	7.802572897	11.21740363	8.500883368	9.146833611	7.473249717

Infinium_Core	BMI	10.37739088	7.833301497	10.01314878	8.399305954	8.796801619	7.381720049
Infinium_OncoArray	BMI	9.953973373	6.154855534	8.752991938	6.93927884	7.563534779	6.521734851
PsychArray	BMI	9.699465121	7.269390162	9.50648778	7.277211483	7.65052003	6.282281431
Axiom_GW_ASI	BMI	7.945143401	5.762027755	7.350324389	6.617551154	6.798231858	5.57868402
Infinium_GSA	BMI	9.770873911	6.198041675	8.501039305	6.259461126	6.936237302	5.687141755
Axiom_GW_CHB	BMI	8.577980916	5.722163626	7.631802721	6.851139683	6.993112274	5.815873803
Axiom_JAPONICA	BMI	7.335879942	5.643265869	5.591773747	5.650391883	6.119495987	4.219911344
Axiom_GW_EUR	BMI	8.585304895	5.869162604	7.779431217	5.751574055	6.823323757	6.452054201
Infinium_Chinese	BMI	7.887695945	5.636621847	7.030659486	5.823508255	6.264610804	5.013266881
Infinium_JSA	BMI	9.483407756	6.719597372	8.25617284	7.117928611	7.855019007	6.018616938
Axiom_UKB	BMI	8.763140247	4.507968673	7.104670572	4.243327312	5.54196411	5.102336073
CytoSNP-850K	BMI	5.758478077	4.114310392	5.121724616	4.376524155	4.807607864	4.028683233
Axiom_PMRA	BMI	8.130531606	5.209743458	7.838088939	5.66383014	6.718774177	5.481052375
Axiom_PMDA	BMI	7.567729635	4.674069214	7.588892038	5.181633855	6.087294717	5.382141282
Affymetrix_6.0	BMI	6.589978509	5.040320906	6.811775636	5.185586283	5.734335337	5.094364135
OmniZhongHua	BMI	5.101608758	3.611025754	4.241465105	3.518847156	3.897608324	3.173323728
Multi-Ethnic_EUR_EAS_SAS	BMI	6.23041694	4.145869495	5.492567397	4.131078341	4.954813672	4.253275384
Multi-Ethnic_Global	BMI	6.043884364	3.999701019	5.53154132	4.286804027	4.872846801	4.163517259
Infinium_GDA	BMI	5.961947354	3.763007749	5.707908163	4.28166587	4.785025155	4.177197499
Axiom_GW_PanAFR	BMI	3.94716665	4.091886819	5.002834467	4.300242284	4.519469223	4.029864261
Infinium_Omni2.5	BMI	3.661989238	2.856098797	3.563161376	2.533901956	3.106377106	2.779844577
Infinium_Omni5	BMI	3.194856736	1.996528499	2.884463971	1.832345885	2.323509855	2.453388763
CytoSNP-12	Type_2_diabetes	11.10498237	5.914840253	10.69184618	7.003308183	8.457642783	6.872106481
Infinium_Core	Type_2_diabetes	9.685046038	5.899891204	9.499401612	6.68750914	8.430878091	6.299603175
Infinium_OncoArray	Type_2_diabetes	8.734302082	5.217217982	7.769589317	5.70533064	7.213920986	5.870594766
PsychArray	Type_2_diabetes	9.423213808	5.193963906	8.958097128	6.174088669	7.579008117	6.02225844
Axiom_GW_ASI	Type_2_diabetes	7.527447754	4.446511473	6.729103679	4.676908727	5.682896943	4.780899628

Infinium_GSA	Type_2_diabetes	9.042366927	4.948965609	8.683704963	5.698216269	6.56153161	5.596497858
Axiom_GW_CHB	Type_2_diabetes	7.950865259	5.278675182	7.864071555	5.497037655	6.587459905	5.518943689
Axiom_JAPONICA	Type_2_diabetes	7.835283724	3.851871538	5.437846435	4.776114684	5.877359161	3.982131047
Axiom_GW_EUR	Type_2_diabetes	8.091165222	4.13507296	7.36882716	4.008553055	5.452051472	4.963860544
Infinium_Chinese	Type_2_diabetes	7.463820691	4.018802581	6.805083144	4.801410227	5.792046704	4.336833113
Infinium_JSA	Type_2_diabetes	9.551383431	5.221370496	8.347899345	5.938523926	7.319306962	5.554079271
Axiom_UKB	Type_2_diabetes	8.46606137	4.092717322	7.702270723	3.871799027	5.477561569	4.951361332
CytoSNP-850K	Type_2_diabetes	6.010468712	3.143452732	5.283525447	3.412131584	4.663747642	3.772695421
Axiom_PMRA	Type_2_diabetes	7.372042085	4.501324652	6.966096624	4.711690098	6.076003362	4.832864859
Axiom_PMDA	Type_2_diabetes	7.439102263	4.335224111	6.971608088	4.607741227	5.884886731	5.161387472
Affymetrix_6.0	Type_2_diabetes	5.628477459	3.569500619	6.376763668	3.927923513	5.123347594	4.068247669
OmniZhongHua	Type_2_diabetes	5.163175952	2.588676926	4.548532376	2.692789585	3.658817084	3.090553351
Multi-Ethnic_EUR_EAS_SAS	Type_2_diabetes	6.304572222	3.305400759	5.487843285	3.593943298	4.643674123	3.877216396
Multi-Ethnic_Global	Type_2_diabetes	5.979799552	3.262214619	5.577207735	3.537028327	4.335880161	3.90034486
Infinium_GDA	Type_2_diabetes	5.878865973	3.244774062	5.606339758	3.442565284	4.453812087	3.825940098
Axiom_GW_PanAFR	Type_2_diabetes	3.950599765	2.913403483	4.911501638	2.972226284	4.045650528	3.422520629
Infinium_Omni2.5	Type_2_diabetes	4.0492446	2.283882434	3.63835349	2.246165156	3.028174021	2.74618528
Infinium_Omni5	Type_2_diabetes	3.607974897	1.987392969	3.156887755	1.899537171	2.513371891	2.408804957

Table S. 18 Mean absolute difference of percentile ranking between PGSs estimated from imputed genotyping data of 23 SNP arrays and PGS estimated from WGS in 6 different populations with PRsice p-value setting of 0.2.

Array_name	Trait	AFR	AMR	EAS	EUR	SAS	VNP
CytoSNP-12	Height	11.87926421	7.57003214	9.319491686	6.270527926	8.762927556	7.027411659
Infinium_Core	Height	10.7623575	6.421446902	9.444286974	6.121521369	8.575574709	6.9664903
Infinium_OncoArray	Height	9.486611996	5.343454393	7.867220962	4.351623855	7.263268387	6.041450145
PsychArray	Height	9.583883585	5.914840253	8.330183925	5.061480026	7.949950025	6.515731293
Axiom_GW_ASI	Height	8.836837781	5.519520966	7.398746536	5.245267955	7.49620485	6.097647392
Infinium_GSA	Height	9.160923828	5.497927896	7.140495087	4.452015541	6.741775085	5.707711325
Axiom_GW_CHB	Height	8.702946299	5.498758398	6.899959058	4.971759898	7.079261127	5.741075365
Axiom_JAPONICA	Height	8.520075712	5.146625252	5.069759385	4.280084898	6.291793694	4.163910935
Axiom_GW_EUR	Height	9.580221596	5.550249566	8.298689846	4.888363655	7.151609436	6.599977954
Infinium_Chinese	Height	7.866639507	4.806119144	6.185043462	4.279294412	6.448618064	4.914847884
Infinium_JSA	Height	9.894008299	6.238736307	7.66526518	4.758723998	6.808268617	5.811444948
Axiom_UKB	Height	8.333085386	3.886752651	5.912619678	2.782904956	5.615985213	5.125464538
CytoSNP-850K	Height	5.822562889	3.844397014	4.721356135	2.99791707	4.723131804	3.817968159
Axiom_PMRA	Height	8.204458014	5.098456095	6.652336861	3.735440241	6.557349626	5.0133653
Axiom_PMDA	Height	7.50501807	4.408308349	6.944050768	3.925156813	6.37501516	5.115721057
Affymetrix_6.0	Height	6.754310276	4.50381616	6.415737591	3.755202384	5.90245106	4.886798469
OmniZhongHua	Height	5.164549198	3.196604905	3.688744016	2.400705113	4.016794844	3.121752173
Multi-Ethnic_EUR_EAS_SAS	Height	6.118497394	3.613517262	5.38233812	2.871044113	4.669184221	4.156824767
Multi-Ethnic_Global	Height	5.984377038	3.649228878	5.303209247	2.83191507	4.864482835	4.044331853
Infinium_GDA	Height	5.928073954	3.577805646	5.276045603	2.757609413	4.470958218	3.900246441
Axiom_GW_PanAFR	Height	4.205336892	3.983921468	4.734347443	3.251662984	4.800916691	3.593474427
Infinium_Omni2.5	Height	3.848521815	2.671727196	3.141140716	1.981352442	3.019810054	2.738607017
Infinium_Omni5	Height	3.424875435	1.798868855	2.560074956	1.320901628	2.260361909	2.323377268
CytoSNP-12	BMI	12.40910828	7.937114335	11.42211514	8.645542253	9.563777334	7.652765967

Infinium_Core	BMI	10.61290256	7.684641514	10.74932288	8.575189025	9.304494377	7.559956853
Infinium_OncoArray	BMI	10.15194967	6.36248121	9.076987276	7.34045034	7.738759875	6.635605631
PsychArray	BMI	10.00844546	7.089171075	10.15841522	7.381160354	8.022716533	6.59052973
Axiom_GW_ASI	BMI	8.082467998	5.757875242	7.516455656	6.498187812	7.130281322	5.715388007
Infinium_GSA	BMI	9.841367204	6.177279107	8.575837743	6.696599726	7.337707688	5.922953672
Axiom_GW_CHB	BMI	8.958141174	5.710536588	7.839269967	7.076428111	7.114807984	5.948345773
Axiom_JAPONICA	BMI	7.760899568	5.568520626	5.670115268	5.867775455	6.436490312	4.417044596
Axiom_GW_EUR	BMI	8.722629491	5.757875242	7.964852608	5.903347312	6.886889901	6.497523778
Infinium_Chinese	BMI	8.044703734	5.280336187	7.073570169	6.348786012	6.666081189	5.126448728
Infinium_JSA	BMI	10.07687889	6.655648664	8.780942933	7.327407325	8.03568068	6.105619331
Axiom_UKB	BMI	8.865447072	4.610120506	7.196003401	4.284037327	5.546146093	5.122610387
CytoSNP-850K	BMI	6.211420371	3.935752311	5.490599017	4.484030212	5.010434048	4.237626764
Axiom_PMRA	BMI	8.57660767	5.177353852	8.056579113	5.78279824	6.717519582	5.527899817
Axiom_PMDA	BMI	7.730001533	4.741339933	7.918005165	5.401784126	6.324413163	5.527309303
Affymetrix_6.0	BMI	6.681985988	4.776221047	6.796028597	5.529052326	6.011182623	5.3019298
OmniZhongHua	BMI	5.506258568	3.632618824	4.404446964	3.848874941	4.059032875	3.218399628
Multi-Ethnic_EUR_EAS_SAS	BMI	6.669855649	4.071124252	5.572483623	4.319213941	5.022979998	4.344411376
Multi-Ethnic_Global	BMI	6.239800788	3.922464268	5.685468632	4.442529712	5.118747412	4.262231513
Infinium_GDA	BMI	6.206842885	3.861007068	5.74452003	4.468615741	4.866573827	4.286541005
Axiom_GW_PanAFR	BMI	3.911462255	4.060327716	5.065428949	4.443320198	4.587635549	4.158989985
Infinium_Omni2.5	BMI	3.740035384	2.79879411	3.764329806	2.725989985	3.299166531	2.804744583
Infinium_Omni5	BMI	3.285948718	2.062968715	2.968316956	1.897165713	2.455660523	2.424847254
CytoSNP-12	Type_2_diabetes	11.20179621	6.287735967	11.0981198	7.371279283	8.593139038	6.988929831
Infinium_Core	Type_2_diabetes	10.2407529	6.162330058	10.34265558	6.841653854	8.655450588	6.575668462
Infinium_OncoArray	Type_2_diabetes	8.94784183	5.333488361	8.394746788	5.471742112	7.571898746	5.941948539
PsychArray	Type_2_diabetes	9.65712337	5.463046782	9.423028471	6.11756894	7.683139498	6.168213813
Axiom_GW_ASI	Type_2_diabetes	7.654473005	4.600984976	7.070420761	4.963459798	5.782009945	4.839754189

Infinium_GSA	Type_2_diabetes	9.607457641	5.12752369	8.797083648	5.786355426	6.891071884	5.592954775
Axiom_GW_CHB	Type_2_diabetes	7.976041435	5.541944539	8.204994961	5.616400998	6.750139051	5.554079271
Axiom_JAPONICA	Type_2_diabetes	7.827501997	4.139225473	5.657123961	4.822358098	5.99696388	4.033407344
Axiom_GW_EUR	Type_2_diabetes	8.089563102	4.07693777	7.658966364	4.099458913	5.750226873	5.167981544
Infinium_Chinese	Type_2_diabetes	7.690406275	4.21812323	6.756660998	5.004565055	6.10653184	4.502570704
Infinium_JSA	Type_2_diabetes	9.731507527	5.112574641	8.350261401	6.157883712	7.63546489	5.543843695
Axiom_UKB	Type_2_diabetes	8.651449576	4.265461884	7.979024943	4.014876941	5.699624876	4.929512314
CytoSNP-850K	Type_2_diabetes	6.429308731	3.356891927	5.59728521	3.414898284	4.863646438	3.850151172
Axiom_PMRA	Type_2_diabetes	7.63089895	4.549493809	7.18340577	4.761095455	6.400107059	4.972324578
Axiom_PMDA	Type_2_diabetes	7.447570613	4.327749587	7.132227891	4.556359655	5.960580627	5.323778817
Affymetrix_6.0	Type_2_diabetes	5.852087677	3.57614464	6.335034014	3.994719555	5.42277759	4.231032691
OmniZhongHua	Type_2_diabetes	5.301873794	2.86025131	4.651281809	2.750099799	3.915172653	3.166532817
Multi-Ethnic_EUR_EAS_SAS	Type_2_diabetes	6.512390112	3.438281192	5.76420383	3.710539941	4.852773282	3.862158289
Multi-Ethnic_Global	Type_2_diabetes	6.275505183	3.527975484	5.751999874	3.560347656	4.469703623	3.966482426
Infinium_GDA	Type_2_diabetes	6.117581897	3.157571278	5.735465482	3.376164484	4.848591299	3.842572909
Axiom_GW_PanAFR	Type_2_diabetes	4.306957093	3.064554975	5.024880322	3.073013213	4.189092551	3.467990205
Infinium_Omni2.5	Type_2_diabetes	4.244016653	2.35032265	3.731261023	2.255255742	3.214272272	2.860351316
Infinium_Omni5	Type_2_diabetes	3.743926248	2.029748607	3.275384228	1.863965313	2.76972746	2.517361111

Table S. 19 Mean absolute difference of percentile ranking between PGSs estimated from imputed genotyping data of 23 SNP arrays and PGS estimated from WGS in 6 different populations with PRsice p-value setting of 0.3.

Array_name	Trait	AFR	AMR	EAS	EUR	SAS	VNP
CytoSNP-12	Height	11.92229259	7.654743416	9.312011842	6.292661526	8.833603071	7.130850025
Infinium_Core	Height	10.84955862	6.298532502	9.437594482	6.187526926	8.586029667	7.072979655
Infinium_OncoArray	Height	9.688708027	5.395776063	7.936114261	4.476915841	7.318052367	6.060149754
PsychArray	Height	9.639042298	5.906535226	8.508912824	5.153176369	8.015188963	6.597615898
Axiom_GW_ASI	Height	8.825622939	5.442284215	7.527478584	5.307321083	7.455221415	6.150301556
Infinium_GSA	Height	9.1451315	5.472182312	7.293635047	4.412096012	6.679045337	5.784084467
Axiom_GW_CHB	Height	8.952877065	5.468860301	7.178681658	5.057132355	7.188829087	5.750523589
Axiom_JAPONICA	Height	8.472469852	5.313556296	4.939452633	4.318818698	6.199790064	4.193239796
Axiom_GW_EUR	Height	9.491647231	5.446436728	8.39159738	4.831448684	7.211829994	6.632653061
Infinium_Chinese	Height	7.843065451	4.840169755	6.210238725	4.296685098	6.429799139	5.020746725
Infinium_JSA	Height	10.04323436	6.146550507	7.730221718	4.796667312	6.899017652	5.862229151
Axiom_UKB	Height	8.252521623	3.817820927	6.026785714	2.799900399	5.618912601	5.117197342
CytoSNP-850K	Height	5.911823877	3.866820587	4.876858151	2.990012213	4.722295407	3.75311004
Axiom_PMRA	Height	8.224370081	5.112574641	6.746819098	3.691173041	6.549822057	5.095348325
Axiom_PMDA	Height	7.414841585	4.360139192	6.897597002	3.889584955	6.336959113	5.164241623
Affymetrix_6.0	Height	6.832127547	4.620917041	6.377157344	3.844132027	5.879450153	4.963860544
OmniZhongHua	Height	5.148070246	3.341112375	3.781257874	2.464339213	3.928136801	3.095671139
Multi-Ethnic_EUR_EAS_SAS	Height	6.068831665	3.639262846	5.399266188	2.869067899	4.691348731	4.208789997
Multi-Ethnic_Global	Height	5.907475264	3.69905904	5.277226631	2.822824485	4.907557262	4.104170603
Infinium_GDA	Height	5.985063661	3.507212916	5.279982363	2.725989985	4.577180591	3.993842908
Axiom_GW_PanAFR	Height	4.230513068	3.98142996	4.737103175	3.224391227	4.78126137	3.640420288
Infinium_Omni2.5	Height	3.921990474	2.632693569	3.221450617	1.979376228	2.95624391	2.738114922
Infinium_Omni5	Height	3.417551457	1.780597796	2.608497103	1.320111142	2.287962998	2.32022786
CytoSNP-12	BMI	12.75676839	8.115672417	11.5933642	8.775181911	9.524048494	7.697940287

Infinium_Core	BMI	10.70468117	7.789284854	10.77924225	8.677952168	9.342132226	7.752267574
Infinium_OncoArray	BMI	10.20665063	6.574259399	9.282092467	7.424241825	7.844564049	6.751740048
PsychArray	BMI	10.31879905	7.195475421	10.1702255	7.572457897	8.258162186	6.742094986
Axiom_GW_ASI	BMI	8.04081287	5.737943177	7.791635173	6.541269283	7.470694753	5.795107395
Infinium_GSA	BMI	9.559165158	6.200533183	8.617961073	6.734147797	7.625846329	5.894215325
Axiom_GW_CHB	BMI	9.032296456	5.899060701	8.184130134	7.116742883	7.179210525	6.172741087
Axiom_JAPONICA	BMI	7.767079174	5.590944199	5.756723986	5.899790126	6.226972955	4.395392416
Axiom_GW_EUR	BMI	8.643667848	5.745417701	8.028628118	6.07606844	6.938746492	6.585904038
Infinium_Chinese	BMI	8.12069001	5.416538631	7.321586042	6.523088111	6.68071813	5.140719482
Infinium_JSA	BMI	9.884395577	6.889850426	8.80180776	7.54518614	7.935731282	6.118216963
Axiom_UKB	BMI	8.928158637	4.729712895	7.187736206	4.365062112	5.570401596	5.131369678
CytoSNP-850K	BMI	6.172053987	3.976446943	5.556736584	4.574145584	5.068145416	4.25465325
Axiom_PMRA	BMI	8.573861179	5.181506366	8.290816327	5.81876534	6.647262265	5.592856356
Axiom_PMDA	BMI	7.656761749	4.782865068	7.896352986	5.587943512	6.275902158	5.582817618
Affymetrix_6.0	BMI	6.882708773	4.953948625	6.994834971	5.431427341	5.99947307	5.372200964
OmniZhongHua	BMI	5.567596888	3.626805305	4.54617032	3.825555613	4.128035597	3.265542328
Multi-Ethnic_EUR_EAS_SAS	BMI	6.63392238	4.330241095	5.913407029	4.271389555	5.096164703	4.342738253
Multi-Ethnic_Global	BMI	6.231790186	4.077768273	5.801996725	4.474149141	5.089891728	4.29293824
Infinium_GDA	BMI	6.065627425	3.926616781	5.780738221	4.433439127	5.099092091	4.280832703
Axiom_GW_PanAFR	BMI	4.044438239	4.027107608	4.973702444	4.455572727	4.724804597	4.193633472
Infinium_Omni2.5	BMI	3.793134228	2.664252672	3.809602545	2.784485927	3.282856796	2.8737363
Infinium_Omni5	BMI	3.309522774	2.090375304	3.04547745	1.896770471	2.45398773	2.490787982
CytoSNP-12	Type_2_diabetes	11.30868052	6.619937048	11.0752866	7.360607725	8.751636201	7.159686791
Infinium_Core	Type_2_diabetes	10.38082399	6.186414637	10.63594419	6.83137754	8.837366856	6.663064531
Infinium_OncoArray	Type_2_diabetes	8.867964689	5.186489382	8.815980096	5.610472355	7.68773968	6.121956885
PsychArray	Type_2_diabetes	9.668338212	5.618350788	9.87654321	6.335347754	7.869655948	6.338577098
Axiom_GW_ASI	Type_2_diabetes	7.819033647	4.453985998	7.197971781	4.839353541	5.818393198	4.87597238

Infinium_GSA	Type_2_diabetes	9.530555867	4.977202701	9.220285336	5.59742934	6.925782345	5.876401487
Axiom_GW_CHB	Type_2_diabetes	8.278155548	5.675655474	8.492772109	5.415222383	6.848415656	5.7749315
Axiom_JAPONICA	Type_2_diabetes	7.941023663	4.316953052	6.007101915	4.969388441	6.10653184	4.234772613
Axiom_GW_EUR	Type_2_diabetes	7.90119953	4.321936068	7.737307886	4.094715998	5.74102651	5.159812768
Infinium_Chinese	Type_2_diabetes	7.768910169	4.261309371	7.201908541	4.961878826	6.284266125	4.641636747
Infinium_JSA	Type_2_diabetes	9.754623834	5.277844679	8.567964223	6.140097783	7.745869246	5.747374181
Axiom_UKB	Type_2_diabetes	8.69768219	4.4548165	8.438838498	3.879308641	5.866486005	5.040135267
CytoSNP-850K	Type_2_diabetes	6.057387949	3.33945137	5.930335097	3.412526827	4.77624299	3.934496252
Axiom_PMRA	Type_2_diabetes	7.863206392	4.80196663	7.690854119	4.597464912	6.573659361	5.059917486
Axiom_PMDA	Type_2_diabetes	7.612360129	4.462291025	7.46606513	4.570588398	5.956398643	5.405072909
Affymetrix_6.0	Type_2_diabetes	5.937228927	3.812837911	6.625960569	3.993533827	5.483416346	4.291560374
OmniZhongHua	Type_2_diabetes	5.390677033	3.015555316	4.805996473	2.740218727	3.810204875	3.345360135
Multi-Ethnic_EUR_EAS_SAS	Type_2_diabetes	6.619274423	3.315366792	5.924429957	3.629119913	4.915084832	4.066082451
Multi-Ethnic_Global	Type_2_diabetes	6.126965744	3.508873921	6.050406274	3.724768684	4.532851569	4.019923942
Infinium_GDA	Type_2_diabetes	6.007264471	3.398417062	6.053949358	3.441379556	4.785861551	3.980950019
Axiom_GW_PanAFR	Type_2_diabetes	4.204421394	3.065385478	5.300059839	3.112142256	4.102943698	3.517888637
Infinium_Omni2.5	Type_2_diabetes	4.22273134	2.464101521	4.075333837	2.275017885	3.31422167	2.922256866
Infinium_Omni5	Type_2_diabetes	3.586918459	2.204154175	3.421831696	1.886494156	2.843330364	2.531336609

Table S. 20 Mean absolute difference of percentile ranking between PGSs estimated from imputed genotyping data of 23 SNP arrays and PGS estimated from WGS in 6 different populations with PRsice p-value setting of 0.5.

Array_name	Trait	AFR	AMR	EAS	EUR	SAS	VNP
CytoSNP-12	Height	11.92275034	7.60076074	9.394290123	6.279618512	8.882532274	7.240882464
Infinium_Core	Height	10.78936467	6.162330058	9.493890149	6.121916612	8.662978158	7.137936193
Infinium_OncoArray	Height	9.779571135	5.390793047	8.072326153	4.440553498	7.365726975	6.185633976
PsychArray	Height	9.738831505	5.842586518	8.484898589	5.245267955	7.936985877	6.664836073
Axiom_GW_ASI	Height	8.842330765	5.456402761	7.481024817	5.274120683	7.494532057	6.175693657
Infinium_GSA	Height	9.120184198	5.537792026	7.479450113	4.32869977	6.609206218	5.762530707
Axiom_GW_CHB	Height	8.884672515	5.58513068	7.354654825	4.994683984	7.105607621	5.872759984
Axiom_JAPONICA	Height	8.570428064	5.240472058	5.038265306	4.311309084	6.124514367	4.186055209
Axiom_GW_EUR	Height	9.53124249	5.401589582	8.357741245	4.833820141	7.266195775	6.67497323
Infinium_Chinese	Height	7.907608012	4.830203722	6.466521794	4.302218498	6.481237532	4.99663407
Infinium_JSA	Height	10.06749504	6.135753972	7.865252583	4.850420341	6.855106829	5.924823633
Axiom_UKB	Height	8.137626711	3.819481932	6.144494835	2.800690885	5.646095491	5.145443594
CytoSNP-850K	Height	5.946155026	3.752211213	4.81898778	3.07538467	4.764115239	3.828006897
Axiom_PMRA	Height	8.149985924	5.058591966	6.814137692	3.805002984	6.631788927	5.181957042
Axiom_PMDA	Height	7.427887421	4.39003729	6.90665155	3.939385555	6.304757842	5.279391849
Affymetrix_6.0	Height	6.940385104	4.537866771	6.456286218	3.78089317	5.813374819	5.059326972
OmniZhongHua	Height	5.24190872	3.319519305	3.850151172	2.482125142	3.895935531	3.177162069
Multi-Ethnic_EUR_EAS_SAS	Height	6.148251057	3.671652451	5.467372134	2.869067899	4.635728355	4.207116875
Multi-Ethnic_Global	Height	5.955538873	3.647567873	5.31068909	2.871439356	4.817226425	4.13497575
Infinium_GDA	Height	6.083479622	3.495585878	5.298878811	2.736661542	4.601017895	3.982623142
Axiom_GW_PanAFR	Height	4.292309136	4.008006046	4.758755354	3.246524827	4.759096859	3.621327003
Infinium_Omni2.5	Height	3.992712641	2.606947986	3.213183422	1.938666213	3.011864286	2.761932319
Infinium_Omni5	Height	3.480491897	1.729937131	2.618732678	1.332363671	2.348601754	2.358119174
CytoSNP-12	BMI	12.81307147	8.2028752	11.69453893	9.183863025	9.710564944	8.038863694

Infinium_Core	BMI	10.93401324	7.933792324	10.98434744	8.783086768	9.322895103	7.89497512
Infinium_OncoArray	BMI	10.41629951	6.695512794	9.436019778	7.604867811	7.96625976	6.843466553
PsychArray	BMI	10.49343016	7.122391183	10.5489418	7.56455304	8.186650273	6.946904919
Axiom_GW_ASI	BMI	8.078806008	5.933941815	7.948711892	6.668537483	7.392073469	5.798453641
Infinium_GSA	BMI	9.720292684	6.303515518	8.808500252	6.702133126	7.757578799	6.079341459
Axiom_GW_CHB	BMI	9.173283042	6.021144599	8.177831318	7.232944283	7.306342814	6.296552186
Axiom_JAPONICA	BMI	7.921569345	5.750400718	6.041745402	5.740902498	6.35494164	4.483674257
Axiom_GW_EUR	BMI	8.962718661	5.772824291	8.178224994	6.121126126	6.836287904	6.59151392
Infinium_Chinese	BMI	8.253665995	5.582639171	7.379850088	6.534154911	6.803250237	5.266006866
Infinium_JSA	BMI	9.937723296	6.901477464	9.124622071	7.50368564	7.956223	6.325782628
Axiom_UKB	BMI	9.172825293	4.760441495	7.057823129	4.435415341	5.772391383	5.191405266
CytoSNP-850K	BMI	6.315100441	4.233072279	5.749637818	4.584421898	5.083618754	4.375118103
Axiom_PMRA	BMI	8.722858366	5.345945901	8.358922273	5.834970297	6.697027865	5.75239355
Axiom_PMDA	BMI	7.833681604	4.942321587	8.066814689	5.602172255	6.445272477	5.633897077
Affymetrix_6.0	BMI	7.09830839	5.045303922	7.220017637	5.319573612	6.195608081	5.521601002
OmniZhongHua	BMI	5.677456565	3.586110673	4.437909423	3.868637084	4.180310387	3.357760928
Multi-Ethnic_EUR_EAS_SAS	BMI	6.783377315	4.305326014	5.992929579	4.347671427	5.104946868	4.544103521
Multi-Ethnic_Global	BMI	6.42450237	4.140055976	6.008282943	4.573355098	5.196114101	4.529734347
Infinium_GDA	BMI	6.332037142	4.012989062	5.89962837	4.363481141	5.130875164	4.420194004
Axiom_GW_PanAFR	BMI	4.215178488	4.120954414	5.101253464	4.507744784	4.824335797	4.223946523
Infinium_Omni2.5	BMI	3.853328176	2.719896353	3.721025447	2.80148137	3.282856796	2.962805493
Infinium_Omni5	BMI	3.352093399	2.164290045	3.070672714	1.890051342	2.495389364	2.511751228
CytoSNP-12	Type_2_diabetes	11.41327608	6.462972037	11.30873646	7.322269168	8.802238197	7.314303036
Infinium_Core	Type_2_diabetes	10.31467931	6.409819864	10.89458932	7.079985297	8.634540672	6.760105663
Infinium_OncoArray	Type_2_diabetes	8.69081596	5.379996512	8.736457546	5.787541155	7.469440158	6.319483812
PsychArray	Type_2_diabetes	9.564887016	6.112499896	9.846230159	6.423091669	7.91398497	6.525966868
Axiom_GW_ASI	Type_2_diabetes	7.798892706	4.750475463	7.150336987	5.060689541	5.945107289	4.992008377

Infinium_GSA	Type_2_diabetes	9.335326066	4.992151749	9.340750189	5.541700098	6.996876059	5.931712963
Axiom_GW_CHB	Type_2_diabetes	8.255496989	5.84341702	8.399864575	5.615215269	6.883126116	5.879354056
Axiom_JAPONICA	Type_2_diabetes	7.879685344	4.380071257	6.04292643	5.060689541	6.276738555	4.211152053
Axiom_GW_EUR	Type_2_diabetes	8.128242863	4.333563106	8.150667674	4.231470027	5.916669803	5.307146007
Infinium_Chinese	Type_2_diabetes	7.85359367	4.423257398	7.289698287	5.095075669	6.2407735	4.71299052
Infinium_JSA	Type_2_diabetes	9.514076916	5.448097734	8.519935752	6.117964183	7.829090711	5.85051729
Axiom_UKB	Type_2_diabetes	8.956539054	4.493019625	8.295540438	3.926342541	5.871086186	5.221029384
CytoSNP-850K	Type_2_diabetes	6.187617441	3.36104444	6.050012598	3.686034884	5.012106841	4.121000252
Axiom_PMRA	Type_2_diabetes	7.832766106	5.001287279	7.601883346	4.850815584	6.760594009	5.121134102
Axiom_PMDA	Type_2_diabetes	7.68170905	4.497172138	7.46803351	4.711294855	6.082276337	5.466191106
Affymetrix_6.0	Type_2_diabetes	5.882527963	3.931599797	7.081050013	4.025153255	5.503908063	4.405431154
OmniZhongHua	Type_2_diabetes	5.426839177	2.87852237	5.008739607	2.726385227	4.076597204	3.444369646
Multi-Ethnic_EUR_EAS_SAS	Type_2_diabetes	6.58265453	3.623483294	5.957892416	3.702239841	4.782097766	4.160072594
Multi-Ethnic_Global	Type_2_diabetes	6.146877811	3.678296473	6.193310658	3.740183156	4.633219165	4.085175737
Infinium_GDA	Type_2_diabetes	6.09286347	3.54541604	6.10276518	3.593152813	4.819317417	4.084782061
Axiom_GW_PanAFR	Type_2_diabetes	4.279034425	3.29045171	5.61500063	3.167476256	4.213766252	3.634219892
Infinium_Omni2.5	Type_2_diabetes	4.248594139	2.498152131	4.217057193	2.297151485	3.427135216	3.038489701
Infinium_Omni5	Type_2_diabetes	3.68007031	2.241526796	3.514739229	1.853293756	2.893095964	2.689988032

Table S. 21 Mean absolute difference of percentile ranking between PGSs estimated from imputed genotyping data of 23 SNP arrays and PGS estimated from WGS in 6 different populations with PRsice p-value setting of 1.

Array_name	Trait	AFR	AMR	EAS	EUR	SAS	VNP
CytoSNP-12	Height	11.92091934	7.667200957	9.409643487	6.317957069	8.957389773	7.227005385
Infinium_Core	Height	10.90265746	6.287735967	9.547823759	6.079625626	8.783001075	7.105261086
Infinium_OncoArray	Height	9.851895423	5.461385777	8.180193374	4.358738227	7.462330787	6.213781809
PsychArray	Height	9.724641297	5.877467631	8.490410053	5.233015426	8.151103416	6.592793367
Axiom_GW_ASI	Height	8.789918544	5.494605885	7.505826405	5.289930398	7.501641428	6.182582987
Infinium_GSA	Height	9.156346342	5.625825312	7.495590829	4.353995312	6.661481008	5.814102261
Axiom_GW_CHB	Height	8.911679686	5.641604863	7.421973419	4.944883384	7.270377759	5.832211357
Axiom_JAPONICA	Height	8.601097224	5.204760441	4.974883472	4.300242284	6.18348033	4.201605411
Axiom_GW_EUR	Height	9.401699621	5.500419404	8.341206853	4.850815584	7.346908051	6.653025794
Infinium_Chinese	Height	7.978787927	4.83269523	6.452349458	4.302613741	6.49169249	5.012577948
Infinium_JSA	Height	10.13249535	6.160669053	7.905407533	4.896663755	6.945437665	5.894215325
Axiom_UKB	Height	8.060038314	3.888413657	6.160241875	2.79871467	5.663241622	5.109028565
CytoSNP-850K	Height	5.974077694	3.771312776	4.795760897	3.073803699	4.670857014	3.818066578
Axiom_PMRA	Height	8.141975323	5.053608949	6.784611993	3.716863827	6.709573814	5.172804075
Axiom_PMDA	Height	7.399735879	4.410799857	6.988142479	3.893932627	6.356614434	5.250259826
Affymetrix_6.0	Height	6.991195205	4.645001619	6.45904195	3.78642657	5.859376634	5.060901675
OmniZhongHua	Height	5.207806446	3.405061083	3.839128244	2.417700556	3.917681843	3.188873929
Multi-Ethnic_EUR_EAS_SAS	Height	6.111173416	3.689093008	5.457530234	2.886458584	4.723550002	4.179854812
Multi-Ethnic_Global	Height	5.949359266	3.747228197	5.344545225	2.884877613	4.886647346	4.121689185
Infinium_GDA	Height	6.052581588	3.538772019	5.263447972	2.723223285	4.651201693	4.008605757
Axiom_GW_PanAFR	Height	4.298030994	4.003853533	4.754818594	3.197909956	4.884556354	3.637074043
Infinium_Omni2.5	Height	3.98470204	2.652625634	3.244283825	1.920089799	3.060375291	2.737622827
Infinium_Omni5	Height	3.445474125	1.738242158	2.625818846	1.306672885	2.376621041	2.355265023
CytoSNP-12	BMI	12.96115316	8.140587498	11.82012157	9.072799782	9.646162403	8.093092561

Infinium_Core	BMI	10.91638992	7.902233222	11.0162352	8.657794782	9.344223218	7.950483434
Infinium_OncoArray	BMI	10.38814797	6.644852129	9.549004787	7.647949282	8.012261575	6.879192649
PsychArray	BMI	10.70857203	7.050967951	10.68476002	7.58668664	8.247289029	7.051229056
Axiom_GW_ASI	BMI	8.187521314	5.930619804	7.978631267	6.613598726	7.459403398	5.807803445
Infinium_GSA	BMI	9.664218474	6.210499215	8.842356387	6.824263169	7.870492345	6.170083774
Axiom_GW_CHB	BMI	9.104849618	6.041907166	8.243968884	7.180376983	7.341471473	6.376468411
Axiom_JAPONICA	BMI	7.891586809	5.714689101	6.112213404	5.79939844	6.385470118	4.477670698
Axiom_GW_EUR	BMI	8.931591752	5.775315799	8.267983119	6.097016312	6.880198728	6.589840797
Infinium_Chinese	BMI	8.203313643	5.678977485	7.425122827	6.487516254	6.998130654	5.371512031
Infinium_JSA	BMI	10.01691381	6.934697572	9.140762787	7.477994854	7.989678866	6.402254189
Axiom_UKB	BMI	9.147191369	4.842661263	7.088529856	4.547269069	5.837630321	5.225851915
CytoSNP-850K	BMI	6.327917404	4.176598095	5.748850466	4.652403669	5.148021295	4.396179768
Axiom_PMRA	BMI	8.759936007	5.338471377	8.446318342	5.823113012	6.861379804	5.848844167
Axiom_PMDA	BMI	7.825899877	4.891660922	7.96760834	5.644067998	6.508002225	5.683204995
Affymetrix_6.0	BMI	7.076565329	5.071049506	7.15978521	5.442889383	6.169679786	5.559197058
OmniZhongHua	BMI	5.614973874	3.576975143	4.635534769	3.84452727	4.350098904	3.329416257
Multi-Ethnic_EUR_EAS_SAS	BMI	6.878360161	4.315292046	5.980331948	4.437391555	5.173531392	4.568806689
Multi-Ethnic_Global	BMI	6.465013126	4.195699657	5.962222852	4.624341427	5.360047842	4.548827633
Infinium_GDA	BMI	6.357671066	4.061158219	5.914588057	4.496677984	5.256334659	4.446471876
Axiom_GW_PanAFR	BMI	4.196639667	4.081920787	5.18116969	4.457548941	4.992869719	4.279257999
Infinium_Omni2.5	BMI	3.883997336	2.861081813	3.83361678	2.885272856	3.308366894	2.976682571
Infinium_Omni5	BMI	3.38917104	2.186713618	3.088388133	1.943409128	2.522990453	2.505157155
CytoSNP-12	Type_2_diabetes	11.33477219	6.658140172	11.12055933	7.396574825	9.076994492	7.286352041
Infinium_Core	Type_2_diabetes	10.41607064	6.555157837	10.82294029	7.123857254	8.883786869	6.801835317
Infinium_OncoArray	Type_2_diabetes	8.843475136	5.347606906	8.773463089	5.836156026	7.683557697	6.334837176
PsychArray	Type_2_diabetes	9.532157987	6.033602139	9.809224616	6.549569383	8.000133823	6.506972002
Axiom_GW_ASI	Type_2_diabetes	7.982907665	4.953948625	7.168052406	5.108909169	5.853940055	4.997519841

Infinium_GSA	Type_2_diabetes	9.518196653	5.007100798	9.515936004	5.63062974	6.972202358	6.029246189
Axiom_GW_CHB	Type_2_diabetes	8.272433689	5.845908528	8.68213026	5.689125683	7.120244562	5.899136275
Axiom_JAPONICA	Type_2_diabetes	8.059580565	4.487206106	6.31574389	5.095075669	6.444854279	4.242744552
Axiom_GW_EUR	Type_2_diabetes	8.143348569	4.474748565	8.059728521	4.37020027	6.028746952	5.358323885
Infinium_Chinese	Type_2_diabetes	8.012203579	4.488036609	7.330246914	5.094680426	6.318976585	4.79152888
Infinium_JSA	Type_2_diabetes	9.483178881	5.704723069	8.517573696	6.194246054	7.619155156	5.903958806
Axiom_UKB	Type_2_diabetes	9.079444568	4.434884436	8.358134921	4.01882937	5.971035584	5.306063398
CytoSNP-850K	Type_2_diabetes	6.271843194	3.439111694	6.16260393	3.769035884	4.970705208	4.168536628
Axiom_PMRA	Type_2_diabetes	8.000759863	4.958931641	7.72904069	4.966621741	6.753902836	5.217879976
Axiom_PMDA	Type_2_diabetes	7.683997794	4.462291025	7.573144999	4.756352541	6.162570414	5.538529069
Affymetrix_6.0	Type_2_diabetes	5.954165627	4.0553447	7.263715671	4.087601627	5.505580857	4.425902305
OmniZhongHua	Type_2_diabetes	5.442631505	3.002267272	5.046532502	2.793576513	4.094161533	3.47734001
Multi-Ethnic_EUR_EAS_SAS	Type_2_diabetes	6.718148132	3.465687781	5.97324578	3.803422013	4.808026062	4.169225561
Multi-Ethnic_Global	Type_2_diabetes	6.216913355	3.658364408	6.24212648	3.810931627	4.754496677	4.127988001
Infinium_GDA	Type_2_diabetes	6.14046933	3.694906527	6.140951751	3.623586513	4.884556354	4.166666667
Axiom_GW_PanAFR	Type_2_diabetes	4.354105204	3.433298175	5.718537415	3.203443356	4.218784632	3.719549162
Infinium_Omni2.5	Type_2_diabetes	4.383401118	2.577049888	4.295005039	2.382523942	3.501992715	3.093998016
Infinium_Omni5	Type_2_diabetes	3.71600358	2.352814158	3.480883094	1.935109028	2.881386411	2.751795163
